# MLO Proteins from Tomato (*Solanum lycopersicum* L.) and Related Species in the Broad Phylogenetic Context

**DOI:** 10.3390/plants11121588

**Published:** 2022-06-16

**Authors:** Alexandr Pozharskiy, Valeriya Kostyukova, Gulnaz Nizamdinova, Ruslan Kalendar, Dilyara Gritsenko

**Affiliations:** 1 Laboratory of Molecular Biology, Institute of Plant Biology and Biotechnology, Almaty 050040, Kazakhstan; aspozharsky@gmail.com (A.P.); valera.kostykova.15@gmail.com (V.K.); lepestochic@mail.ru (G.N.); 2 Department of Molecular Biology and Genetics, Al-Farabi Kazakh National University, Almaty 050040, Kazakhstan; 3 Institute of Biotechnology HiLIFE, University of Helsinki, P.O. Box 65, 00014 Helsinki, Finland; ruslan.kalendar@helsinki.fi; 4 National Laboratory Astana, Nazarbayev University, Nur-Sultan 010000, Kazakhstan

**Keywords:** Mildew locus *o*, seven transmembrane proteins, Solanaceae, phylogeny

## Abstract

MLO proteins are a family of transmembrane proteins in land plants that play an important role in plant immunity and host–pathogen interactions, as well as a wide range of development processes. Understanding the evolutionary history of MLO proteins is important for understanding plant physiology and health. In the present work, we conducted a phylogenetic analysis on a large set of MLO protein sequences from publicly available databases, specifically emphasising MLOs from the tomato plant and related species. As a result, 4886 protein sequences were identified and used to construct a phylogenetic tree. In comparison to previous findings, we identified nine phylogenetic clades, revealed the internal structure of clades I and II as additional clades and showed the presence of monocotyledon species in all MLO clades. We identified a set of 19 protein motifs that allowed for the identification of particular clades. Sixteen SlMLO proteins from tomato were located in the phylogenetic tree and identified in relation to homologous sequences from other Solanaceae species. The obtained results could be useful for further work on the use of MLO proteins in the study of mildew resistance in Solanaceae and other plant families.

## 1. Introduction

MLO proteins are a large family of proteins that are present in all land plants and green algae. The term MLO originated from the first discovered member of this family: the product of the *Mlo* gene (Mildew resistance locus *o*) in barley, which confers resistance to powdery mildew [1]. The effect of mildew resistance has been identified as being the result of a recessive mutation in the gene [2]. MLO proteins have been found to be omnipresent as a series of paralogues in all land plants and potentially originated from ancient algae [3]. In general, different plant species contain 10–15 MLO homologs and the maximum of 39 proteins was discovered in soybean [4]. The association between MLO homologues and mildew susceptibility has been further identified in other plant species, including Arabidopsis thaliana [5], rice and wheat [6], tomato [7], pepper [8] and other plant species, indicating the existence of a universal mechanism of MLO-mediated host–pathogen interactions between different plants and mildew fungi [9]. 

All known MLO proteins share the same topology, consisting of seven relatively conserved hydrophobic transmembrane domains, three extracellular loops and N-terminus, and three intracellular loops and C-terminus [10]. Studies on the expression and regulation of MLO protein genes in *Arabidopsis thaliana* revealed the involvement of proteins from the MLO family in a wide range of physiological processes, including morphogenesis and responses to biotic and abiotic stress, as receptors coupled with G-proteins [11]. For example, AtMLO4 and AtMLO11 participate in the process of root morphogenesis, as shown by a study on aberrant root development in loss-of-function mutations [12]. MLO proteins include a calmodulin-binding domain at the C-terminus, indicating their involvement in the calcium signalling system of plants [13,14]. The specific regulatory roles of particular MLO homologues have strong associations with their phylogenetic history [11]. The phylogenetic groups that are referred to as clades IV and V have been identified as containing MLO homologues that are responsible for susceptibility to powdery mildew in monocotyledons and dicotyledons, respectively [4,9,15,16]. These particular proteins are known to be involved in defence responses against pathogens; however, the ascomycete fungi that causes powdery mildew (Erysiphaceae family) have developed molecular mechanisms to exploit these responses to suppress plant immunity and assist in host penetration by the fungus [17,18].

Tomato (*Solanum lycopersicum* L.) and its closely related species, such as potato (*Solanum tuberosum* L.), eggplant (*Solanum melongena* L.) and pepper (*Capsicum annuum* L.), are among the most important vegetable crops in the world. Tomato plants are susceptible to different powdery mildew fungi, the most common species of which are the hemi-endophyte *Leveillula taurica* and the epiphyte *Oidium neolycopersici* (syn. *Pseudoidium neolycopersici*) [19]. The latter predominantly occurs in warmer regions [20] whereas the former has spread worldwide and has become a global problem [21]. *O. neolycopersici* is the predominant threat to greenhouse cultivation in Europe, Asia, Africa and North and South America [22] and is not limited to tomato, but also affects a variety of species from the Solanaceae family and other families [23]. Thus, investigations into the physiological and molecular mechanisms of powdery mildew resistance in the tomato plant are important and MLO proteins attract particular attention as potential mildew susceptibility factors and targets for breeding and genetic engineering. The genotype *ol-2*, which confers powdery mildew resistance in domestic tomato cultivars [24,25], was identified as having a loss-of-function mutation in the locus that corresponds to the *Mlo* genes in barley and *Arabidopsis thaliana* with similar effects [7]. This locus, named *SlMLO1*, was the first to be identified out of the genes that encode MLO proteins in tomato. Subsequently, 15 additional SlMLO proteins have been identified, and SlMLO1 has been confirmed as being the principal factor for mildew susceptibility in tomato, as its removal induced strong resistance to *O. neolycopersici*. Two other homologs, SlMLO5 and SlMLO8, have been found to complement SlMLO1; their removal increases mildew resistance when combined with SlMLO1 silencing but does not affect susceptibility alone. All three proteins belong to clade V [26]. In addition to *O. neolycopersici*, the loss-of-function mutation of *SlMlo1* has also been shown to confer resistance to the powdery mildew caused by *L. taurica*; however, it may be incomplete without the simultaneous silencing of other *Mlo* genes [27]. *SlMlo1* is considered to be a promising target for the development of tomato varieties that are resistant to powdery mildew. For example, induced mutagenesis [28] and targeted CRISPR/Cas genome editing [29,30] were successfully used to obtain resistant tomato genotypes by inducing a loss-of-function mutation in *SlMlo1* and the homologous gene *CaMlo2* in pepper [31].

The scope of the present article lay in two main directions. First, we extended the known phylogenetic data on MLO proteins using a broad selection of protein sequence data from a variety of plant genera and species, which were available in the NCBI and UniProt databases. Unlike previous studies, we did not only focus on the fully annotated and well-curated sets of MLO proteins. We used a large sample to re-examine and clarify global phylogeny to account for broad plant diversity. Second, we aimed to identify the locations of MLO homologs in tomato (*Solanum lycopersicum* L.) and related species from the Solanaceae family within the global phylogenetic landscape and describe the groups of MLO homologs in Solanaceae species that correspond to previously identified SlMLO proteins. 

## 2. Results

### 2.1. Data Acquisition and Filtering

**Figure 1:**
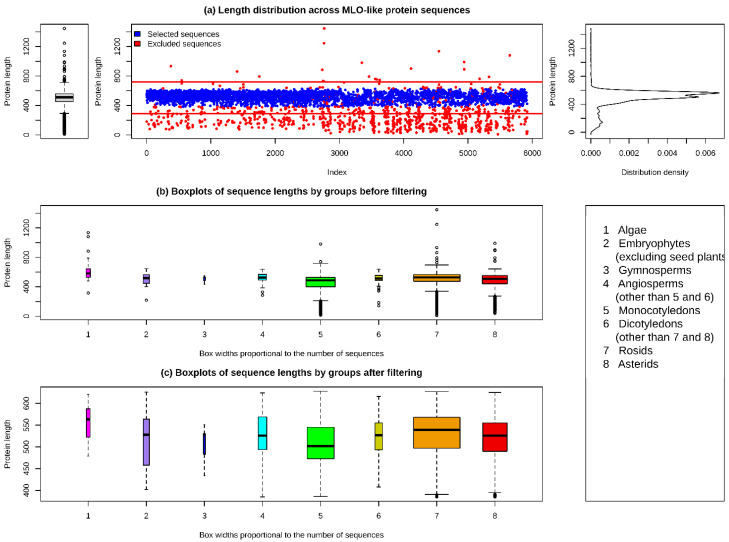
The distribution of sequence lengths in the MLO protein dataset and the results of data filtering.

The search results for MLO proteins included 4474 and 3110 sequences from the UniProt and NCBI databases, respectively. Both datasets were merged and any duplicated sequences were removed. The final dataset included 5924 protein sequences, with lengths ranging between 9 and 1446 amino acid residues, a mean and median of 476.1 and 512, respectively, and first and third quartiles of 452 and 559, respectively. A graphical summary was used to examine the distribution of the sequence lengths within the dataset (Figure 1, top panel). The sequence lengths had a bimodal distribution with a high number of short outliers. Most of these short sequences represented incomplete protein sequences. Based on the graphical summary, the 15th and 99th percentiles (384 and 628.77 amino acid residues, respectively) were selected as the thresholds for the selection of sequences for further analyses. The sequences that were explicitly indicated as incomplete in the description header were also removed from the dataset.

The final filtered dataset included 4886 sequences (Supplementary Material, Table S1), with lengths varying from 385 to 628 amino acid residues. The sequences represented 151 plant genera, predominantly belonging to the rosids and asterids groups of dicotyledons (69 and 28 genera, respectively) and monocotyledons (27 genera) (Table 1).

In total, 396 sequences were identified in relation to the data from Kusch et al. (2016) [3] using a local BLAST search. Sequences with an identity above the 99.5% threshold were considered almost exact matches. Some of the reference sequences had matches with multiple MLO proteins in our dataset because of the presence of different protein isoforms. Some accessions also had non-specific matches with related species because of the high similarity of the protein paralogs.

### 2.2. Phylogenetic Analysis

The selected protein sequences were aligned with MAFFT using the BLOSUM62 substitution matrix. After removing the positions with a gap frequency of ≥99%, the total length of the alignment was 1116 amino acid residues. The general consistency of the alignment was checked by a manual inspection of the positions of seven conserved transmembrane (TM) domains, which were identified according to previous studies [3]. As no shifts in TM domains were found, the resulting alignment was considered to be suitable for phylogenetic analysis. After the manual examination, 370 relatively conserved positions were selected to build a neighbour-joining tree (NJ).

**Figure 2:**
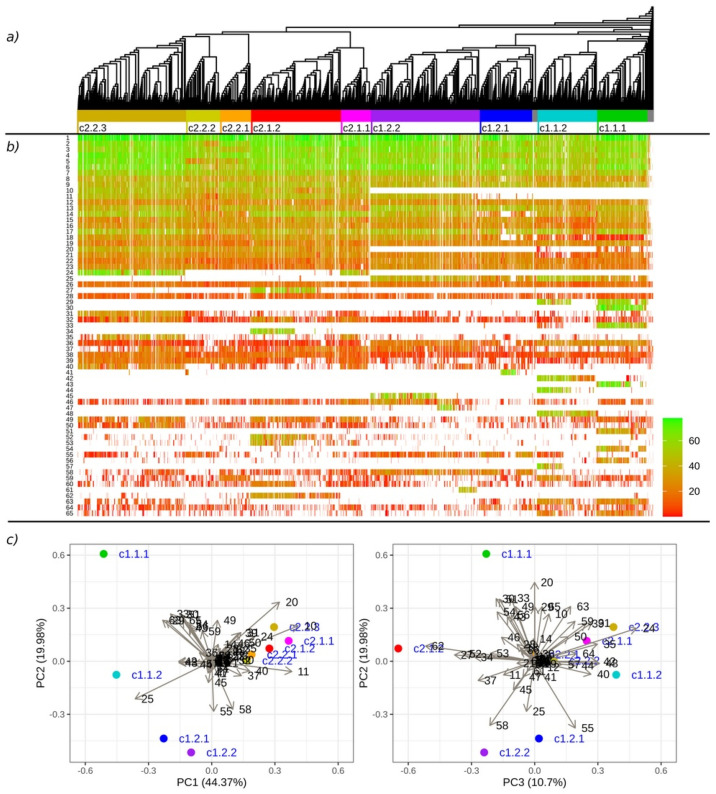
The results of the phylogenetic analysis and MEME motif search of the 4886 MLO proteins: (**a**) The neighbour-joining tree of MLO proteins; (**b**) the occurrence of the selected motifs across the MLO proteins, with colours indicating the motif matching scores; (**c**) a principal component analysis of the frequency of the motif occurrences in the defined phylogenetic clades. The motif numeration is in accordance with the MEME results in the Supplementary Material (File S2).

The MLO-like protein from *Chlorella sorokiniana* (UniProt accession A0A2P6U4B6_CHLSO) was arbitrarily selected to be the root of the tree as it was the most distant sequence. Based on the general topology of the tree, nine clades were identified (Figure 2A). The basal part of the tree included MLO proteins from various algae species and a mixed set of sequences from various angiosperm species (Supplementary Material, Figure S1A). The multiple sequence alignment showed that these proteins were more diverse and had a poor relationship with the majority of the other sequences (Supplementary Material, Figure S2). Regardless of the probable reasons for this deviation, such as the individual diversification of the protein homologues or low data quality, we considered this basal group as an outgroup and made no phylogenetic inferences on its content.

We distinguished the main clades of the tree on a hierarchical basis (two high-level superclades, four level two clades and nine level three clades), with the levels designated in the clade identifiers as separate numbers (e.g., c1.2.1). Although the classification of the sequences was limited to three levels, the final clades had their own substructures. Detailed plots of the identified clades are presented in the Supplementary Material (Figure S1). Using data from Kusch et al. (2016), we identified conformity between our clustering pattern and the previously described clades. We further referred to clades according to Kusch et al. by using their original numbering with Roman numerals followed by “K.” (e.g., I K., IV K., etc.).

Superclade c1 included two level two clades (c1.1 and c1.2) and four level three clades (c1.1.1, c1.1.2, c1.2.1 and c1.2.2). Clades 1.1 and 1.2 included proteins from the Embryophyta group (liverwort (*Marchantia*), lycophyte (*Selaginella*), moss (*Physcomitrium*), etc.) that were at the base of the downstream clades. Clade c1.1.1 included these species as well. Clade c1.1.2 included two well-separated subclades, both consisting of monocotyledons, dicotyledons and basal angiosperms. When combined, both clades corresponded to clade I K.

Clade c1.2 contained embryophyte proteins and was a separate small clade that was at the base of the downstream clades. Clade c1.2.1 began with proteins from two gymnosperm species (*Picea sitchensis* and *Araucaria cunninghamii*) and included three distinct subclades: monocotyledons with basal angiosperms, dicotyledons and a separate subclade of rosids (dicotyledons). Clade 1.2.2 consisted of two distinct subclades of monocotyledons and three subclades of dicotyledons; additionally, a small subclade of MLO proteins from species of the Myrtaceae family was located at the base of the clade. Both clades corresponded to clade II K.

Superclade c2 consisted of two level two clades (c2.1 and c2.2) and five level three clades (c2.1.1, c2.1.2, c2.2.1, c2.2.2 and c2.2.3). All clades predominantly consisted of angiosperm species. Clade c2.1.1 included a distinct subclade of monocotyledons and a mixed subclade of dicotyledons with the addition of monocotyledons and basal angiosperms. Clade c2.1.2 began with the accession from *Araucaria cunninghamii* and included MLO proteins from all groups of angiosperms. These two clades corresponded to clades IV K. and III K., respectively.

Clades c2.2.1 and c2.2.2 (clades VI K. and VII K., respectively) were compact groups that mainly consisted of proteins from dicotyledons. Clade c2.2.3 had a complex substructure and consisted of two subclades of dicotyledons with basal angiosperms and a small subclade of monocotyledons at the base of the clade. This clade corresponded to clade V K.

**Figure 3:**
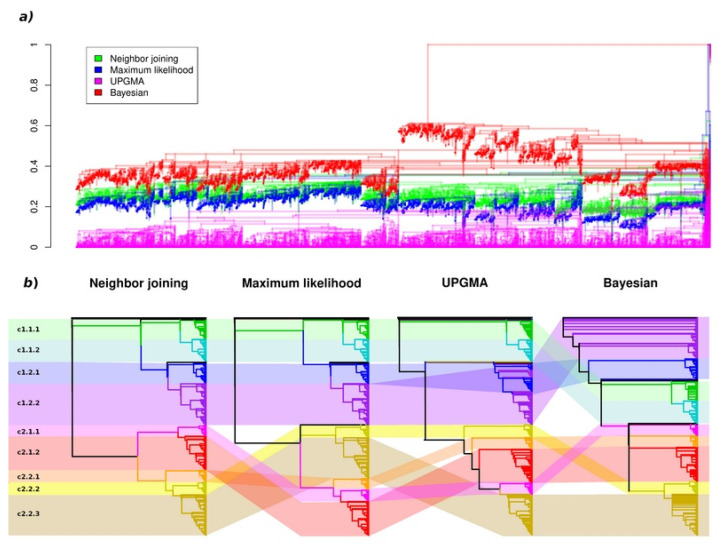
A comparison of the phylogenetic trees obtained using four different methods: (**a**) a superposition of the four aligned trees; (**b**) the consistency of the global topologies of the four trees.

In order to verify the observed phylogenetic structure, we used three additional tree-building methods: UPGMA, maximum likelihood (ML) and Bayesian trees (Figure 3 and Supplementary Material, File S2). For consistency, all methods were applied using the same JTT amino acid substitution model (“Jones” in MrBayes software). The nine clades described above were clearly identified in all trees, with minor deviations in their composition; however, they had different positions relatively to each other. Superclades c1 and c2 were observed in all four trees. Superclade c1 had an identical structure in the NJ and ML trees. The level two clade c1.1 (consisting of clades c1.1.1 and c1.2.2) was observed in all trees; however, it was parallel to clade c1.2 in the NJ and ML trees, at the base of c1.2 in the UPGMA tree and descended from c1.2 in the Bayesian tree. Clades 1.2.1 and 1.2.2 were clearly resolved from each other in all trees except for the UPGMA tree, in which subclusters from within the two clades were mixed into clade c1.2. In the Bayesian tree, clade c1.2.2 appeared at the base part of the whole tree, with clades c1.2.1, c1.1 and superclade c2 descending from it. The composition of superclade c2 was less consistent across the four trees, with varying positions of the five clades. The most similar representation of superclade c2 was observed in the NJ and ML trees, where two level two clades were separated; however, clade c2.2.1 was transferred from clade c2.2 to c2.1 in the ML tree. In general, the nine initially defined clades were shown to be stable phylogenetic units, as supported by the four independent methods of phylogenetic tree construction. For clarity, the following discussion is based on the NJ tree, with accounts of support from the other methods.

A thorough examination of the revealed clades demonstrated that the internal phylogenetic structures of these clusters contained groups that were consistent with known plant divisions. For example, the monocotyledon subclade in clade c2.1.1 (Supplementary Material, Figure S1B) had a distinct separation of the Poaceae family, which consisted of subgroups that corresponded to the subfamilies of Pooideae (genera *Triticum, Hordeum* and *Aegilops*), Oryzoideae (genus *Oryza*) and Panicoideae (genera *Panicum* and *Zea*). These notable clusters were identified in comparison to known taxonomy across all identified clades; however, a detailed discussion of all systematic groups was beyond the scope of the present study.

The MLO accessions from the NCBI database were provided with homologue identifiers that were inherited from the *Arabidopsis thaliana* MLO proteins (AtMLO) as part of the automatic annotation process. For convenience, we referred to these groups of MLO proteins without specific prefixes: MLO1, MLO2, etc. These homologues demonstrated clear distribution patterns across the described clades (Table 2).

### 2.3. Motif Search

The MEME motif search was set for run parameters and resulted in 200 motifs with lengths of between 5 and 20 amino acid residues. The general MEME report indicated that the E-value of the identified motifs exceeded 0.05 before the middle point of the search. Thus, the obtained set of 200 motifs included all significant motifs under the specified search conditions.

The obtained motifs were matched against the whole MLO dataset using the “universalmotif” package. We selected 65 of the most frequent motifs (≥100 occurrences) to check the specificity of the phylogenetic clades (Table 3 and Figure 2B,C). A principal component analysis was applied to the matrix of the frequencies of the motif occurrences in the clades (Supplementary Material, Table S2) in addition to an examination of the heatmap of the motif-matching scores for each sequence with respect to phylogeny. Motifs 10 (global consensus YQFSNDPERFRFTR), 20 (ETSFGRRHLSFW) and 25 (FIKHHFSGPWKRSAILGWLL) strongly indicated a separation between superclades c1 and c2, with motif 10 only occurring in most of the c2 sequences, motif 20 only occurring in c2 with a partial occurrence in clade c1.1 and motif 25 almost solely present in superclade c1. Clade c1.1.1 had the highest number of distinct motifs: motifs 30 (DDSTIHTETSTVMSLEEDDH), 33 (MDRHDSLTEITRELTMRRQS) and 43 (ANETSSRVGTPLLRPSASIS) appeared in the clade with high matching scores and motifs 51 (AARRKRRLGIFT) and 54 (ETDAGTYTEIELQPPSTVTS) were in the clade with lower scores. These motifs, along with motifs 42 (SSLFSSRFYJCSEEDY) and 48 (SLWGIKERSCFMKNH), distinguished clades c1.1.1 and c1.1.2, further supporting their identification as separate clades. Motifs 29 (LENAGITGPFSGTKLKPRDD), 44 (HTTRSVCSLESTIDERDEI) and 57 (DBEGBGEEEKVETLFDLFQK) supported the separation of clade 1.1.2 into two subclades. Some of the other motifs showed less specificity to particular clades, e.g., motif 24 (SSRPTTPSHGMSPVHLLHNY) for c2.1.1 and c2.2.3, motif 27 (EEEHRRRLLWYERRFLAGGS) for c2.1.2 and motif 45 (KNYDPEZVLKPKVTHVQQHD) for clade c1.2.2.

An overview of the identified motifs in the NCBI GenBank database (data not shown) using the protein BLAST search revealed that these motifs either belonged to MLO proteins or unidentified plant proteins. The BLAST search of the arbitrarily selected unidentified matches showed that these proteins were the most similar to MLO proteins. The same results were observed for motifs 1–9 and others belonging to all MLO sequences, regardless of the clade. Thus, the identified motifs were found to be strictly specific to the MLO protein family.

**Figure 4:**
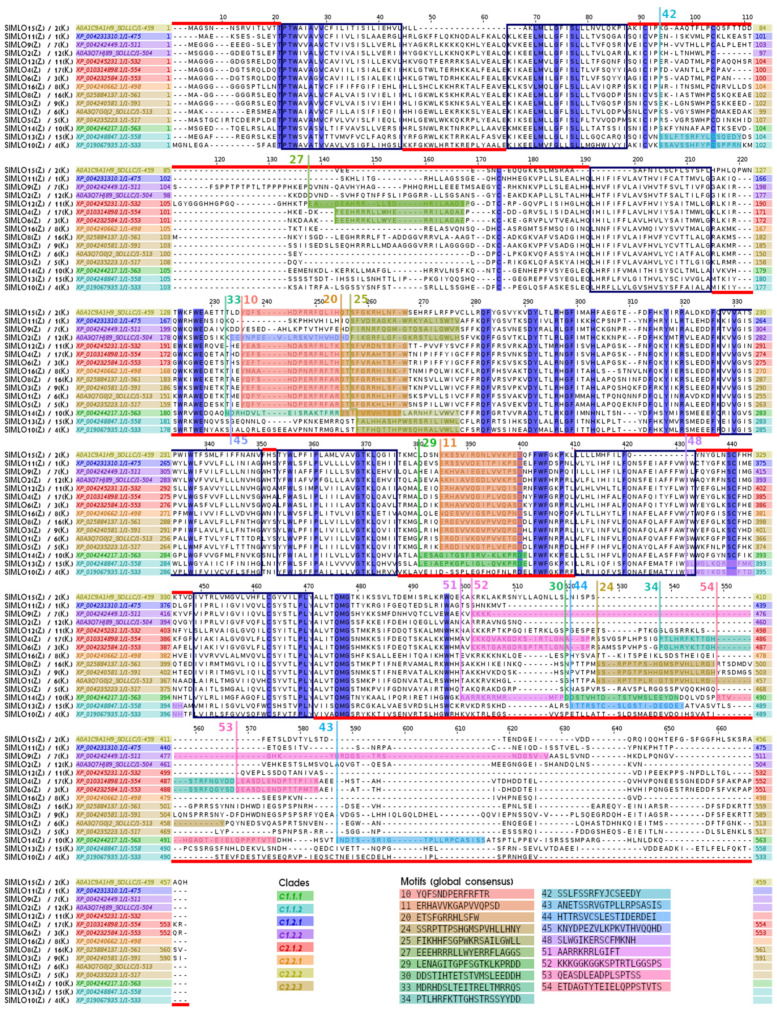
Multiple sequence alignment of the 15 MLO proteins from *Solanum lycopersicum* L. with the clade-specific motifs. The seven transmembrane domains are shown as boxes, according to Kusch et al. (2016). The red lines indicate the extracellular (above) or intracellular (below) localisation of the domain. The motifs shown in the alignment were enumerated in accordance with Table 3 and the corresponding sequences are listed below. The fully conserved positions are shown in blue. The SlMLO homologue names are listed on the left, according to Zheng et al. (2016) (Z.) and Kusch et al. (2016) (K.).

An examination of the identified clade-specific motifs from the selection of MLO sequences from *S. lycopersicum* demonstrated their occurrences with respect to the general protein structure (Figure 4). Most clade-specific motifs belonged to the intracellular C-terminus: motifs 24, 30, 34, 43, 44, 51, 52, 53 and 54. Motifs 42 (characterising clade c1.1.2) and 27 (characterising clade c2.2.3) were found in the first extracellular loop (alignment positions 88–191). Motifs 11 (all clades except c1.1) and 29 (clade c1.1.1 and subclade 2 of clade c1.1.2) were located in the third intracellular loop (377–410) and represented variations in the same protein regions. Motifs 10, 20, 25, 33 and 45 represented variable regions in the second intracellular loop (215–327) and had overlaps.

### 2.4. Clade-Specific Examination of MLO Proteins from Solanum lycopersicum and Related Species

A total of 219 MLO sequences belonging to species of the Solanaceae family were identified in nine clades (Table 4). The family was represented by six *Solanum* spp. (*S. chacoense, S. chilense, S. lycopersicum, S. melongena* and *S. tuberosum*), four *Nicotiana* spp. (*N. attenuata, N. sylvestris, N. tabacum* and *N. tomentosiformis*), three *Capsicum* spp. (*C. annuum, C. baccatum* and *C. chinense*) and *Petunia hybrida.*

We identified the tomato MLO sequences from our dataset using a BLAST search against the whole tomato genome assembly that was created by the Tomato Genome Consortium and compared our results to data from previous studies (Table 5; see the extended version in the Supplementary Material, Table S3). Sixteen MLO homologues were independently identified by Zheng et al. (2016) [26] and Kusch et al. (2016) [3]. The same names for the tomato MLOs that were presented by Kusch et al. and Zheng et al. referred to different genomic features, except for *SlMlo5* (Solyc03g095650.3.1); thus, when appropriate, we mention both names. We considered the classification by Zheng et al. as the primary classification because of its consistent use in preceding and subsequent studies.

Consistent with previous studies, no MLO proteins from tomato or other Solanaceae species were present in clade c2.1.1 (IV K.), suggesting that the corresponding homologues were lost by a common ancestor of the family. All other clades contained Solanaceae sequences, which formed compact groups within distinct subclusters corresponding to the asterids species (Supplementary Material, Figure S1). The Solanaceae sequences usually appeared in close neighbourhoods with MLO proteins from *Ipomoea* and *Cuscuta*, representing the Convolvulaceae family of the order Solanales and other species of the order Lamiales (e.g., *Salvia*, *Dorcoceras*, etc.).

Clade c1.1.1 (I K. A) (Supplementary Material, Figure S1B) included Solanaceae sequences as a compact subcluster among the other dicotyledons. Three genera (*Nicotiana*, *Solanum* and *Capsicum*) were well distinguished. The protein sequences had a high similarity (Figure S3A) and the most variable between-genera domains were the first extracellular loop (alignment positions 82–151) and the C-terminus (428–573). The transmembrane domains were fully conserved between the genera, except for TM domain 5 (304–326; 313 I > V in *Capsicum*). Three MLO protein isoforms belonged to tomato: the primary sequence XP_004244217.1; XP_025887927.1 with a deletion of 59 amino acids before the second TM domain (59–81); and A0A3Q7HEQ0_SOLLC, with the motif VFTAPFL absent in the other species following the sixth TM domain (360–382). This protein was identified as Solyc07g063260.4.1 in the Sol4.0 genome, corresponding to SlMLO14 from Zheng et al. and SlMLO10 from Kusch et al. This protein showed a high similarity to the sequences from wild and cultivated potato (*S. chacoense* and *S. tuberosum*, respectively) with only amino acid changes, mainly in the C-terminus.

Clade c1.1.2 (I K. B) (Supplementary Material, Figure S1C) contained two groups of Solanaceae MLO sequences, which were separated into two subclades. Both groups contained notable changes between the genera and variations in the TM domains (Supplementary Material, Figure S3B). The most variable domains in both groups were the first extracellular loop (alignment positions 81–161) and the C-terminus (461–633). Both groups included multiple isoforms from MLO proteins in different species, but with significant changes. The tomato MLOs were present in group 1 as four isoforms. The corresponding genomic feature of tomato was Solyc02g083720.4.1, named SlMLO10 by Zheng et al. and SlMLO4 by Kusch et al. The potato accession M0ZVS4_SOLLTU had significant protein alterations, which affected the second intracellular loop and TM domains 4 and 5, separating it from the *Solanum* genus and the whole Solanaceae family in group 1. Similarly, two MLO isoforms from *S. tuberosum* (M1BMX7_SOLLTU and M1BMX9_SOLLTU) and the MLO accessions from *C. chinense* (A0A2G3BE56_CAPCH) and *C. baccatum* (A0A2G2VT84_CAPBA) were formally classified as belonging to group 2 (highlighted green), which varied from the other sequences, separating it from the Solanaceae and placing it outside of the asterids species subclusters (M1BMX7_SOLLTU). The tomato MLOs in group 2 included four isoforms. This protein was identified as Solyc10g044510.2.1 (named SlMLO by Zheng et al. and SlMLO15 by Kusch et al.).

Clade c1.2.1 (II K. A) consisted of seven *Solanum* accessions, five sequences from *Nicotiana* spp. and a single *C. annuum* sequence (Supplementary Material, Figure S1D). The genera formed distinct subgroups within the Solanaceae cluster; however, the *Nicotiana* subcluster extended to species of the Lamiales order (*Olea europaea* and *Cuscuta australis*). The *Nicotiana* sequences were clearly distinct from the *Solanum* and *Capsicum* MLOs (Supplementary Material, Figure S3C). The tomato isoforms only differed by single amino acid changes (305 V > A in A0A1C9A1H3_SOLLC and 359 N > D in A0A1C9A1G7_SOLLC) and minor changes in the wild tomato species *S. pennellii* and *S. chilense*. The corresponding genomic feature of *S. lycopersicum* was Solyc01g102520.4.1 (named SlMLO11 by Zheng et al. and SlMLO1 by Kusch et al.).

Clade c1.2.2 (II K. B) contained three groups of Solanaceae MLOs, which were distributed into subclades of dicotyledon sequences (Supplementary Material, Figure S1E). Group 1, with relatively high between-species variation, formed a mixed cluster of *Nicotiana*, *Solanum* (wild and domestic potato) and *Capsicum* sequences. The tomato sequences were distinct and were represented by four isoforms. These proteins were highly similar to the sequence from *S. chilense* (A0A6N2B5C6_SOLCI) and differed notably from *S. tuberosum* and *S. chacoense* (Supplementary Material, Figure S3D). This tomato protein corresponded to the Solyc08g015870.3.1 genomic feature, or SlMLO2 and SlMLO12 according to Zheng et al. and Kusch et al., respectively. Group 2 also had a high level of variation and consisted of three distinct subgroups, which corresponded to the genera *Nicotiana*, *Capsicum* and *Solanum*. The most notable member of this group was the *N. tabacum* MLO isoform (XP_016445575.1), which had a prolonged insertion (391–464) that disrupted TM domain 5. The tomato MLOs were represented by three isoforms. The corresponding genomic feature was Solyc06g082820.4.1, identified as SlMLO9 by Zheng et al. and SlMLO7 by Kusch et al. Group 3 contained relatively similar sequences from the three genera. Although the genus *Solanum* was represented by two proteins from potato (*S. tuberosum*) and wild tomato (*S. pennellii*)*,* no identified sequences from *S. lycopersicum* were included.

Clade c2.1.2 (III K.) included three groups of Solanaceae sequences (Supplementary Material, Figures S1G and S3E). Two of the groups (highlighted blue and green) originated from one branching point and internal structure, reflecting the separation of the three genera. These groups had a relatively high similarity between the species and genera. Group 1 (blue) included three isoforms of the tomato protein. The identification of this protein in the tomato genome provided the feature ID of Solyc02g038806.1.1, whereas the corresponding accessions from the data from Kusch et al. (SlMLO17) referred to the unplaced feature Solyc00g007200.2.1 (or SlMLO4 according to Zheng et al.). A comparison of the corresponding protein sequences (Sol4.0) showed the full identity, except for the absence of 64 amino acids at the beginning of Solyc02g038806.1.1. Additionally, the accession A0A6N2BEN5_SOLCI from *S. chilense* was only present in positions that differed from the primary tomato protein isoform (337 A > B and 491 V > E). Group 2 contained four tomato protein isoforms. Group 3 (highlighted yellow) was located in a separate subclade within c2.1.2. Compared to the other two groups, these proteins included inserted regions in the first extracellular loop (82–164), demonstrating the differences between the genera. There was also a series of deletions that shortened the intracellular C-terminus (435–580). The tomato protein was represented by two isoforms: XP_004245231.1 (primary) and A0A1C9A1H0_SOLLC, with minor amino acid changes. The corresponding feature was Solyc08g067760.4.1 (named SlMLO12 by Zheng et al. and SlMLO11 by Kusch et al.). The sequences from the wild tomato species (*S. pennellii* and *S. chilense*) were highly similar. The sequences from *S. tuberosum* and *S. chacoense* (XP_006356610.1 and A0A0V0IKU6_SOLCH, respectively) differed in several amino acid positions and A0A0V0IKU6_SOLCH had a deletion of 64 amino acids at the beginning.

Clades c2.2.1 (VI K.) and c2.2.2 (VII K.) contained compact groups of MLO sequences from two Solanaceae genera: *Solanum* and *Nicotiana* in c2.2.1 and *Solanum* and *Capsicum* in c2.2.2 (Supplementary Material, Figures S1H,I and S3F). The group in c.2.2.1 corresponded to the tomato protein XP_004240662.1 (named SlMLO16 by Zheng et al. and SlMLO8 by Kusch et al.). Two SlMLO16 isoforms from *S. lycopersicum* were present and K4C428_SOLLC had a deletion of 20 amino acids (28–48) in the intermediate region between the first TM domain and the first intracellular loop. The same deletion appeared in the sequence from *S. chilense* (A0A6N2C9Z0_SOLCI). The sequences from two *Nicotiana* spp. Were presented in two isoforms, one of which had a long deletion that removed the C-terminus. The group in c2.2.2 only included single sequences from *S. lycopersicum* and *S. tuberosum* and five sequences from the *Capsicum* spp. (Supplementary Material, Figure S3G). The protein accessions from the *Capsicum* spp. had a high similarity and two of the three isoforms from *C. annuum* contained an initial deletion of 64 amino acids. The sequence from *S. lycopersicum* A0A1C9A1H9_SOLLC was identified by the BLAST search as Solyc02g077570.3.1, with an identity of 98.45%; thus, it was not matched against the same sequence from the Kusch et al. dataset (SlMLO2) with the selected threshold of 99.5%. According to Zheng et al., the corresponding SlMLO protein was SlMLO15. The number of variations in this sequence compared to *S. tuberosum,* including a full alteration of the region of TM domain 3, demonstrated the low quality of the original data.

Clade c2.2.3 (V K.) contained four groups of Solanaceae MLOs, which were located in different subclades (Supplementary Material, Figure S1J). SlMLO1 (according to Zheng et al.), the primary factor of powdery mildew susceptibility in tomato, was located in the biggest group, along with a diverse set of sequences from the genera *Solanum*, *Capsicum* and *Nicotiana* and *Petunia hybrida*. This group of homologues showed notable variations between the genera (Supplementary Material, Figure S3H). SlMLO1 (genomic feature Solyc04g049090.3.1; identified as SlMLO6 by Kusch et al.) had three isoforms that only differed by minor amino acid changes (A0A3Q7G0J2_SOLLC, Q56BA6_SOLLC and A0A1C9A1D2_SOLLC). Interestingly, a frameshift mutation was detected in the third intracellular loop of the MLO protein from *N. sylvestris*, which disrupted the subsequent sequences. This mutation could indicate a variant conferring powdery mildew resistance; however, the present data were not sufficient to confirm this. The second group containing SlMLO5 (according to Zheng et al. and Kusch et al.; Solyc03g095650.3.1) was located in proximity to the group of SlMLO1 and only consisted of sequences from the *Solanum* spp. Among the five isoforms from *S. lycopersicum*, the most notable were XP_010318227.1, with an initial deletion of about 70 amino acids (the same isoform was present for *S. pennellii*), and A0A1C9A1G2_SOLLC, with an insertion of 37 amino acids (307–344) that replaced and extended TM domain 4. The groups including SlMLO3 (Solyc06g010030.4.1; named SlMLO9 by Kusch et al.) and SlMLO8 (Solyc11g069220.2.1; named SlMLO16 by Kusch et al.) were closely related to each other and separated from the first two groups in the phylogenetic tree. Both included MLO sequences from the three genera, *Solanum*, *Capsicum* and *Nicotiana*.

## 3. Discussion

### 3.1. General Phylogenetic Landscape of MLO Proteins in Land Plants

As MLO proteins belong to a very old and diversified protein family, the importance of large-scale phylogenetic studies across a wide range of plant taxa to understand the evolution of the protein structures and functions is undoubted. Previous studies have focused primarily on thoroughly curated sets of MLO proteins from a relatively small selection of plant species. Here, we presented a significant expansion of the data for phylogenetic analysis. Our dataset included protein sequences representing 151 plant genera that were annotated automatically by the NCBI and UniProt databases. While this approach helped to extend the phylogenetic data, it also had some obvious disadvantages. First, the large sample size of analysed sequences limited the applicability of the bootstrap method to the verification of tree topology as this method has low reliability for larger datasets [32,33]. The alternative approach for verifying the observed phylogenetic structures that was used in the present work was a comparison of tree topologies that resulted from several different algorithms. Second, the detailed inspection of the tree topology showed that the results of the branching were uncertain at the individual sequence level. The presence of protein isoforms, variations within the same species and close homologues from other closely related species (from the same genus) also added uncertainty to the structure of the terminal nodes of the trees. Another problem was the incomplete representation of MLO orthologues from particular plant species, which inevitably limited the possible conclusions on MLO phylogeny at the genera and species level. Finally, a notable amount of negative branch length artefacts, which were caused by the neighbour-joining algorithm, was observed. However, our analysis resulted in a clear structure on a larger scale. The nine identified clades were supported by the four independent phylogenetic methods, except for clades c1.2.1 and 1.2.2, which were only resolved by three methods (however, the UPGMA method that failed to separate these clades is known to be less reliable than the more specialised phylogenetic algorithms [34]). Thus, we considered the revealed phylogenetic structure to be suitable as a basis for further detailed analyses. To put our work into the context of previous findings, we followed the work of Kusch et al., who presented the most comprehensive phylogenetic analysis of MLO proteins to date [3]. The data from Kusch et al. were used as a reference set of sequences for the identification and comparison of global clustering patterns. Although not all of the sequences from the mentioned study were retained in our dataset after data filtering, the comparison showed a strong correlation between the two clade structures. We revealed the internal heterogeneity of clades I K. and II K. They corresponded to the combined clades c1.1.1–c1.1.2 and c1.2.1–c1.2.2, respectively. Clade c1.1.2 consisted of two distinct subclades, which could potentially be considered as separate clades. Similar to clade I, we identified clade c1.1 as the oldest because of the inclusion of non-seed land plants.

Based on the comparison to the mentioned study, we considered our results to be consistent with the known phylogeny of MLO proteins and extend it further to cover a larger diversity of plant species. Unlike previous studies, we identified MLO proteins from all of the main groups of seed plants (basal angiosperms, monocotyledons and dicotyledons) in each clade (Table 1). Clades V, VI and VII K. were previously described as not including monocotyledons because the selection was limited by the Poaceae species [3]. Furthermore, the corresponding clades of c2.2.3, c2.2.1 and c2.2.2 did not contain Poales but included species from other monocot orders. Thus, the absence of MLO proteins in these clades was a feature of the Poaceae family or, more likely, the Poales order rather than monocotyledons in general and these clades were not specific to eudicot plants, as was supposed by the aforementioned authors. In contrast, our dataset lacked MLO sequences from gymnosperms: only four proteins belonged to *Araucaria cunninghamii* and only a single sequence from *Picea sitchensis* was present.

The results of the present study and the previous phylogenetic classification, each of which had its own limitations, complemented each other in terms of phylogenetic inferences as there was consistency between the clades. Clade c1.1.1 (I K. A) at least originated from as long ago as the common ancestor of land plants, which was supported by the inclusion of non-seed plants in both the present and previous results. As MLO homologues of this clade could be traced in both gymnosperms (based on Kusch et al.) and angiosperms, these data potentially indicated their high evolutionary importance. Moreover, this clade contained the highest number of specific motifs that were not retained by the other clades, indicating a certain level of functional conservation. Clade c1.1.2 (II K. B) was probably the result of the duplication and diversification of the *Mlo* gene in the common ancestor of angiosperms as no gymnosperm accessions were present in either our study or the work of Kusch et al. Similarly, clade c1.2.2 diverged from clade c1.2.1, which included gymnosperms, and appeared to share the common ancestor of seed plants. The divergence between the level two clades of c1.1 and c1.2 most probably occurred before the separation of seed plants. Clades c2.1.1 (IV K.) and c2.1.2 (III K.) contained gymnosperms; however, while the former was strongly supported by multiple sequences from Kusch et al., the latter only included a single MLO protein from *A. cunninghamii* in our study. Finally, clades c2.2.1 (VI K.), c2.2.2 (VII K.) and c2.2.3 (V K.) seemed to be the most recently diversified as they included only angiosperm species. However, the comparison of the four alternative trees showed that the relative positions of the clades within superclade c2 was ambiguous, so the described level two clades c2.1 and c2.2 should only be considered as provisional.

The low diversity of protein sequences representing gymnosperms, basal angiosperms and non-seed land plants significantly limited the reliability of these global phylogenetic inferences. Similarly, the absence of sequences from monocotyledons in the previous studies was due to the selection of species being limited to one family; the absence of gymnosperms or non-seed plants in particular clades does not necessarily imply their later origin when the selection of corresponding species lacks diversity.

The MLO sequences that were retrieved from the NCBI database had their own identifiers of MLO homologues, based on their homology with MLO proteins from *A. thaliana*. The distribution of these homologues among the clades (Table 2) showed certain patterns. Clades c1.1.1, c1.1.2, c1.2.1 and c1.2.2 demonstrated the predominant inclusion of particular homologous series (MLO11, MLO4, MLO13 and MLO1, respectively), whereas other clades, such as c2.1.2, c2.2.2 and c2.2.3, included an admixture of several identifiers without correlations to the internal structures of the clades or plant taxonomy. We suggest that the presence of MLO homologues in clades represents their evolutionary history. For example, clade c1.2.1 likely represents a continuous line of paralogues that were inherited from the ancestral protein, which retains a sufficiently high level of similarity to allow their exact identification across a wide range of taxonomic groups. In contrast, clade c2.2.3 may be the result of multiple independent duplication events with subsequent diversifications within the taxonomic groups at the different levels. As this clade corresponded to clade V (Kusch et al.), which was previously described as being associated with the processes of plant immunity, the frequent occurrence of these independent events could be a sign of the coevolution of plants and pathogens. The redundancy of MLO homologues that have defensive functions could be a mechanism to reduce the consequences of interactions between MLO proteins and fungal pathogens (powdery mildew). Further studies on the effects of different MLO homologues could shed light on the evolution of the host–pathogen interactions that involve this protein family.

Although we used the most diverse MLO protein set to date, non-seed land plants, gymnosperms, basal angiosperms and basal dicotyledons remained underrepresented, in addition to many orders of mono- and dicotyledons, which consist of species of low economic interest despite their importance in global plant diversity. A deeper understanding of the evolution of MLO proteins greatly depends on the expansion of the available genomic data to a wider range of plant species, particularly taxa with an ancient history (“living fossils”). The development of technologies that could lower the costs of genomic analyses and the growing interest in genome-wide diversity studies provide a basis for future progress in broad-scale phylogenetic research, including a deeper understanding of the long evolutionary history of the MLO protein family in land plants.

### 3.2. Tomato and Related Species (Solanaceae family) in the Phylogenetic Landscape of MLO Proteins

This study focused on the global phylogenetic landscape of MLO proteins from tomato (*Solanum lycopersicum* L.) and its relatives from the Solanaceae family. Studies on the impact of MLO proteins on plant physiology and health, including responses to pathogens, need to consider the consistency nomenclature of homologues within the same species. The similarity between paralogues of species with different degrees of relatedness is also important to extend the applicability of discovered phenomena to a wider range of species and predict the consequences of gene and protein structure variations, based on phylogenetic and taxonomic relationships. In the case of the tomato plant, the 16 basic SlMLO homologues were identified [26] based on previous discoveries [7] and laid the foundation for further studies, including the targeted modification of MLO genes to achieve powdery mildew resistance [29]. An alternative name for the SlMLOs was proposed in the most comprehensive investigation of MLO evolution to date because of the independent identification of proteins [3]. We used both classifications in our study, in addition to Sol4.0 genomic features, thereby assuring an unambiguous reference to particular SlMLO proteins (Table 5).

The three most important Solanaceae genera, *Solanum*, *Capsicum* and *Nicotiana*, were well represented in the phylogenetic trees, although the data on MLO sequences from some species were limited. The three main species representing their genera, *S. lycopersicum*, *C. annuum* and *N. tabacum*, contained multiple sequences of MLO isoforms, which provided some information on the existence of alternative MLO forms. Although our dataset could not clarify whether the identified variations were of a biological nature or were the results of errors in data generation, processing and annotation, similar patterns in isoform variations were identified across the plant species and MLO paralogues: long (about 60–70 amino acids) deletions at the beginning of the sequence and the removal of the N-terminus, first TM domain and first intracellular loop and the partial removal of the second TM domain. These sequences included (names according Zheng et al.): SlMLO14 (XP_025887927.1; Solyc07g063260.4.1), SlMLO9 (XP_019069984.1; Solyc06g082820.4.1), SlMLO4 (A0A3Q7E877_SOLLC; Solyc02g038806.1.1), SlMLO6 (XP_019067917.1; Solyc02g082430.4.1) and SlMLO5 (XP_010318227.1; Solyc03g095650.3.1) in *S. lycopersicum*; a paralogue of SlMLO13 (XP_033511957.1) in N. tormentosiformis; and the paralogues of SlMLO13 (XP_016545636.1), SlMLO15 (A0A2G3A723_CAPAN and G9BBX3_CAPAN) and SlMLO8 (XP_016548525.1) in *C. annuum*. Additionally, initial deletions were present in the unique proteins of other species; for example, the homologues of SlMLO4 (A0A0V0IJD9_SOLCH) and SlMLO6 (A0A0V0IKK2_SOLCH) in *S. chacoense* (Supplementary Material, Figure S3E). The correspondence features Solyc02g038806.1.1 and Solyc00g007200.2.1 were placed in the same line of observation. Although the protein sequence data were not sufficient for definite conclusions, considering that the primary origin of our data was the automatic annotation of the genomic data from the NCBI and UniProt databases, we presumed the presence of the signal for alternative splicing, which is persistent in Mlo genes across Solanaceae species and orthologous series. Indeed, the splicing variants of MLO proteins were identified in tomato by transcription analysis; SlMLO9 and SlMLO15 transcripts, without the initial 60 amino acids, were identified in leaves and fruits, respectively, among other transcript variations [26]. Further clarification of this matter requires a detailed investigation of *Mlo* genes and transcripts.

Clade c2.2.3 (V K.) contained SlMLO proteins that were previously found to be associated with susceptibility to *Oidium neolycopersici* in *A. thaliana* and other dicot species [4]. Four of the groups of Solanaceae MLO homologues likely differentiated from two ancestral MLO proteins. In terms of tomato homologues, SlMLO3 and SlMLO8 resulted from gene duplication in the common ancestor of the Solanaceae family and SlMLO5 originated from the SlMLO1 protein in the common ancestor of the *Solanum* genus. The SlMLO1 protein is known to play a central role in the powdery mildew susceptibility of tomato [7], while SlMLO5 and SlMLO8 have minor impacts on the infection [26]. Although these homologues were related to the AtMLO2, AtMLO6 and AtMLO12 proteins of *A. thaliana*, as well as the other sequences belonging to this clade, a homology with species outside of the family could not be determined between the complex mixed structure of the clade. This could be the result of independent duplication events across the families and orders of angiosperms. To illustrate this, SlMLO5 and its paralogues were specific to the *Solanum* genus and thus, their diversification from SlMLO1 was the most recent. This protein was shown to have only a minor impact on powdery mildew susceptibility [26]; thus, the role of SlMLO5 as a “supplementary” MLO protein that is unaffected by mildew fungi during infection could be speculated. Considering the attribution of clade c2.2.3 (V K.) to plant–pathogen interactions in dicot plants [4], as confirmed by the positions of the known tomato homologues, the corresponding neighbouring sequences could be considered as likely candidates for mildew resistance targets in the Solanaceae family.

## 4. Materials and Methods

### 4.1. Taxonomic Grouping Convention

To put our work into a systemic context, we assigned all protein sequences to eight groups according to their species of origin. For convenience, we defined some high-level taxonomic groups to exclude downstream taxa when they were also present. Therefore, we used the following groups: algae, embryophytes, gymnosperms, angiosperms, monocotyledons, dicotyledons, rosids and asterids. The embryophyte group included all land plants except seed plants. The angiosperm group included all flowering plants that do not belong to mono- or dicotyledons, i.e., basal angiosperms. The dicotyledon group excluded plant orders that are classified as rosids or asterids.

### 4.2. Data Acquisition and Filtering

The amino acid sequences of the MLO proteins were retrieved from the UniProt [35] and NCBI [36] databases in FASTA format. A data search on UniProt was performed using the keywords “MLO” in the “Name” field and “Viridiplantae” in the “Taxonomy” field. The data from NCBI were retrieved using the search term “MLO” and were then filtered to exclude incorrect matches. A custom R script was used to unify the format of the sequence headers, merge the datasets and perform the preliminary data examination and filtering. Based on the sequence length, outliers were discarded using the threshold percentile lengths, which were selected based on the graphical data summary. Moreover, all sequences containing the words “fragment” or “partial” in their description were excluded.

The accession numbers of all used sequences are provided in the Supplementary Material, Table S1.

### 4.3. Identification of Previously Reported Plant-Specific MLO Proteins

To make our investigation consistent with previous studies, we compared our dataset to the previously discovered plant-specific MLO proteins. We used the MLO protein sequences from a previous study [3] as the reference dataset for the comparison (Table 2). First, we built a local BLAST database from our combined MLO protein sequence dataset. Second, we performed a protein BLAST search of the reference dataset against our local database. Third, the BLAST results were matched to the MLO protein data using a general R script. For each complete, or almost complete, match (threshold of 99%), the protein name of the query (e.g., SlMLO1, AtMLO2, etc.) was attached to the FASTA header line of the respective subject sequence.

### 4.4. Phylogenetic Analysis and Motif Search

A multiple sequence alignment of the selected proteins was performed using MAFFT [37] on the Galaxy web platform [38], with the BLOSUM62 substitution matrix. The resulting alignment was inspected, refined and filtered manually using UGENE [39] and R/Bioconductor [40,41]. Positions with a percentage of gaps of 99% or higher were filtered out and relatively conserved parts of the alignment were selected and merged together for further phylogenetic analysis.

The phylogenetic analysis was conducted using R with the packages “phangorn” [42] and “ape” [43]. The neighbour-joining tree was calculated using a distance matrix based on the JTT amino acid substitution model [44] (functions “phangorn::dist.ml()”, “phangorn::NJ()” and “ape::plot.phylo()”). After the inspection of the resulting tree, the most distant algae sequence was arbitrarily selected for the re-rooting tree.

The additional phylogenetic trees were calculated using different methods to validate the observed phylogenetic structure. All methods were used with the JTT model. The UPGMA tree was calculated using the same distance matrix as for the neighbour-joining tree and the built-in R function “hclust()”. The maximum likelihood tree was calculated using FastTree software [45] on the Galaxy web platform. The Bayesian phylogenetic tree was calculated using MrBayes software [46] with the default parameters for the selected amino acid model and the calculations were conducted for 65,000,000 MCMC generations with a relative burn-in fraction of 50%. All trees were examined and compared with respect to the structure of the initial NJ tree.

A motif search was performed using MEME 5.3.3 [47]. The motif search results were loaded into R using the “universalmotif” package. Motifs with more than 100 occurrences were matched with our MLO dataset and were accounted for in the phylogenetic clades. The matrix of motif occurrence frequencies in the clades was plotted as a heatmap and was then subjected to a principal component analysis to identify the motifs that contributed to clade separation.

The MLO proteins from the Solanaceae family were additionally selected according to the results of the phylogenetic analysis. Full-length sequences were realigned with MAFFT and examined in accordance with their clade assignment.

## 5. Conclusions

In the present study, we attempted to use a wide selection of the available data on MLO protein sequences from land plants to identify new details about the known phylogeny of this protein family. Compared to previous studies, we identified the internal structures of the clades that are traditionally referred to as I and II and suggested their separation as two pairs of distinct clades: c1.1.1–c1.1.2 and c1.2.1–c1.2.2. We showed that all nine identified clades actually contained mono- and dicotyledons and basal angiosperm species, in contrast to previous findings. The MLO sequences from tomato and the related species of the Solanaceae family were identified in homologous groups. This information could be further used to study natural and artificial mildew resistance in the Solanaceae family.

## Figures and Tables

**Table 1:** A representation of the high-level taxonomic groups in the MLO dataset.

Group	Number of Sequences	Number of Genera	Number of Species	Number of Sequences per Clade								
				c1.1.1	c1.1.2	c1.2.1	c1.2.2	c2.1.1	c2.1.2	c2.2.1	c2.2.2	c2.2.3
Algae	19	13	14	-	-	-	-	-	-	-	-	-
Embryophytes	49	3	4	15	-	-	-	-	-	-	-	-
Gymnosperms	5	2	2	-	-	4	-	-	1	-	-	-
Angiosperms	99	4	4	5	18	9	16	3	14	4	8	22
Monocotyledons	966	27	54	83	68	171	328	87	160	9	24	26
Dicotyledons	76	5	5	10	10	8	17	1	14	5	7	4
Rosids	2888	69	129	261	296	216	429	130	423	195	221	708
Asterids	784	28	42	67	113	49	135	34	151	50	29	153

**Table 2:** The distribution of the MLO homologue identifiers provided by the NCBI database within the identified phylogenetic clades.

Clade (Size)	MLO1	MLO2	MLO3	MLO4	MLO5	MLO6	MLO7	MLO8	MLO9	MLO10	MLO11	MLO12	MLO13	MLO14	MLO15	MLO17
c1.1.1 (441)	0	0	0	0	0	0	0	0	0	0	175	0	0	59	0	0
c1.1.2 (505)	0	0	0	282	0	0	0	0	0	0	0	0	0	0	0	0
c1.2.1 (457)	5	0	0	0	0	0	0	0	0	0	0	0	201	0	1	0
c1.2.2 (925)	426	0	0	0	0	0	0	0	0	0	0	0	4	0	34	0
c2.1.1 (255)	40	0	0	0	0	0	0	0	0	0	0	0	0	0	0	0
c2.1.2 (763)	0	0	0	0	25	0	15	78	140	117	0	0	0	0	0	0
c2.2.1 (263)	0	1	144	0	0	1	0	0	0	0	0	1	0	0	0	0
c2.2.2 (289)	0	12	0	0	0	31	0	0	8	0	0	113	0	0	0	0
c2.2.3 (913)	0	65	1	0	0	292	0	0	0	0	0	101	1	0	0	1

**Table 3:** Protein motifs with specific distribution in the found phylogenetic clades.

Motif No.	Consensus Sequence	Regular Expression	MEME ID	Number of Occurrences	E-Value	Specific Clades
10	YQFSNDPERFRFTR	Y[QE]F[SA][NH]DP[ES]RFR[FL][TA][RH]	MEME-10	2339	7.4 × 10^−851^	c2
11	ERHAVVKGAPVVQPSD	[ED][RK][HG]A[VA][VI][KQE]GX[PL][VL]VQP[SG]D	MEME-11	3330	6.9 × 10^−1030^	c2 and c1.2.2
20	ETSFGRRHLSFW	[EQD]T[ST]F[GV]RRHL[SN]FW	MEME-20	2329	3.3 × 10^−597^	c2
24	SSRPTTPSHGMSPVHLLHNY	SSRP[TA]TP[ST]HG[MS]SP[VI]HLL[HR]N[YH]	MEME-24	1005	3.7 × 10^−433^	c2.1.1 and c2.2.3
25	FIKHHFSGPWKRSAILGWLL	F[IV][KR]H[HR][FTA]S[GH]P[WG][KS][RK][SN][AR][IV]L[GSI]W[LMV][LH]	MEME-25	1978	8.6 × 10^−304^	c1
27	EEEHRRRLLWYERRFLAGGS	E[EG]EH[RH]R[RK]LL[WS][YF]E[RH]RFL[AS][GA][GAD]S	MEME-27	456	1 × 10^−213^	c2.1.2
29	LENAGITGPFSGTKLKPRDD	LE[NIS]A[GE]ITG[PY]F[ST]G[TA][KQ][LV][KR]PRD[DE]	MEME-29	548	3.6 × 10^−165^	c1.1
30	DDSTIHTETSTVMSLEEDDH	DDST[IV][HR]T[ED]TSTV[MC]S[LI]E[ED]DDH	MEME-30	362	8 × 10^−134^	c1.1.1
33	MDRHDSLTEITRELTMRRQS	MDRHDSL[TS]EI[TA]RE[LK]T[ML]RRQ[ST]	MEME-33	400	9.5 × 10^−113^	c1.1.1
34	PTLHRFKTTGHSTRSSYYDD	[PH]TLHRFKTTGHSTRSSYY[DE][DE]	MEME-34	305	4.5 × 10^−106^	c2.1.2 *
42	SSLFSSRFYJCSEEDY	SSLF[ST]S[RK]FY[IL]CSEEDY	MEME-42	324	3 × 10^−47^	c1.1.2
43	ANETSSRVGTPLLRPSASIS	ANETSSR[VA]GTPLLRP[SC]AS[IV]S	MEME-43	198	1.7 × 10^−48^	c1.1.1
44	HTTRSVCSLESTIDERDEI	H[TA][TA]RS[VT]CSL[ED][ST]TID[ED][RE][DR][ED][IE]	MEME-44	243	1.7 × 10^−35^	c1.1.2 *
45	KNYDPEZVLKPKVTHVQQHD	[KE][NE]YD[PT]E[QE]VLK[PKT]K[VF]THV[QH][QDE]H[DA]	MEME-46	304	8.3 × 10^−33^	c1.2.2
48	SLWGIKERSCFMKNH	SLW[GE][IFL]K[EQ]RSCFMKNH	MEME-51	226	6.9 × 10^−28^	c1.1.2
51	AARRKRRLGIFT	AARR[KR]RR[LH]G[IM][FY]T	MEME-56	276	2 × 10^−24^	c1.1.1
52	KKKGGKGGKSPTRTLGGSPS	KKK[GK]GKGGKSPTRTLGGS[PS]S	MEME-57	283	1.6 × 10^−23^	c2.1.2 *
53	QEASDLEADPLSPTSS	Q[ED]ASDLEA[DE]PL[ST]PT[SP][ST]	MEME-60	268	8.2 × 10^−17^	c2.1.2 *
54	ETDAGTYTEIELQPPSTVTS	ETDAGT[YG][TN]E[IV]ELQPPST[VI]T[ST]	MEME-62	228	7.7 × 10^−10^	c1.1.1

* Partial presence of the motif in the clade.

**Table 4:** The occurrences of the MLO sequences from species of the Solanaceae family in the nine phylogenetic clades.

Species	Total	c1.1.1	c1.1.2	c1.2.1	c1.2.2	c2.1.1	c2.1.2	c2.2.1	c2.2.2	c2.2.3
***Solanum***	103	5	18	7	19	0	22	5	2	25
*chacoense*	6	1	0	0	2	0	3	0	0	0
*chilense*	10	0	2	1	2	0	3	1	0	1
*lucopersicum*	49	3	8	3	7	0	10	2	1	15
*melongena*	1	0	0	0	0	0	0	0	0	1
*pennellii*	12	0	2	1	2	0	2	1	0	4
*tuberosum*	25	1	6	2	6	0	4	1	1	4
***Capsicum***	50	4	11	1	12	0	9	0	5	8
*annuum*	30	2	7	1	6	0	5	0	3	6
*baccatum*	10	1	2	0	3	0	2	0	1	1
*chinense*	10	1	2	0	3	0	2	0	1	1
***Nicotiana***	65	3	12	5	13	0	11	4	0	17
*attenuata*	16	1	2	1	3	0	4	2	0	3
*sylvestris*	6	0	2	1	0	0	1	0	0	2
*tabacum*	33	2	4	2	9	0	4	2	0	10
*tomentosiformis*	10	0	4	1	1	0	2	0	0	2
***Petunia hybrida***	1	0	0	0	0	0	0	0	0	0
Total	219	12	41	13	44	0	42	9	7	50

**Table 5:** The tomato MLO proteins.

Accession *	Source	Protein Name in NCBI	No. of Isoforms	Genomic Feature ID **	Clade	Zheng 2016	Kusch 2016	Clade (K.)
XP_004231310.1	NCBI	MLO13	3	Solyc01g102520.4.1	c1.2.1	SlMLO11	SlMLO1	II
XP_004242449.1	NCBI	MLO1	3	Solyc06g082820.4.1	c1.2.2	SlMLO9	SlMLO7	II
A0A3Q7HJ89_SOLLC	UniProt		4	Solyc08g015870.3.1	c1.2.2	SlMLO2	SlMLO12	II
A0A1C9A1H9_SOLLC	UniProt		1	Solyc02g077570.3.1	c2.2.2	SlMLO15	SlMLO2	VII
XP_004245231.1	NCBI	MLO9	2	Solyc08g067760.4.1	c2.1.2	SlMLO12	SlMLO11	III
XP_010314898.1	NCBI	MLO9	2	Solyc02g038806.1.1 ***	c2.1.2	SlMLO4	SlMLO17	III
XP_004232584.1	NCBI	MLO8	4	Solyc02g082430.4.1	c2.1.2	SlMLO6	SlMLO3	III
XP_004240662.1	NCBI	MLO3	2	Solyc06g010010.3.1	c2.2.1	SlMLO16	SlMLO8	VI
XP_025884137.1	NCBI	MLO6	2	Solyc11g069220.2.1	c2.2.3	SlMLO8	SlMLO16	V
XP_004240581.1	NCBI	MLO6	4	Solyc06g010030.4.1	c2.2.3	SlMLO3	SlMLO9	V
A0A3Q7G0J2_SOLLC	UniProt		3	Solyc04g049090.3.1	c2.2.3	SlMLO1	SlMLO6	V
XP_004235223.1	NCBI	MLO2	6	Solyc03g095650.3.1	c2.2.3	SlMLO5	SlMLO5	V
XP_004244217.1	NCBI	MLO11	3	Solyc07g063260.4.1	c1.1.1	SlMLO14	SlMLO10	I
XP_004248847.1	NCBI	MLO4	4	Solyc10g044510.2.1	c1.1.2	SlMLO13	SlMLO15	I
XP_019067935.1	NCBI	MLO4	4	Solyc02g083720.4.1	c1.1.2	SlMLO10	SlMLO4	I

* The most complete isoform found (see extended table in the Supplementary Material for all present isoform accessions); ** identified by BLAST using the whole tomato genome assembly SL4.0 and annotation ITAG4.0 as a reference database (https://solgenomics.net/organism/Solanum_lycopersicum/genome; accessed on 9 July 2021); *** Solyc00g007200 according to Zheng et al. (2016).

## Data Availability

No novel data were generated in the present study. A list of the publicly available data accessions that were used in the study is provided in the Supplementary Materials, Table S1. All scripts that were used in the study are appended as a part of the Supplementary Materials, File S1.

## Supplementary Materials

The following are available online at https://www.mdpi.com/article/10.3390/plants11121588/s1, Table S1: A list of the analysed accessions from the NCBI and UniProt databases and their assignment to clades, Table S2: The frequencies of occurrence of the selected MEME motifs in the nine phylogenetic clades of the MLO proteins, Table S3: The MLO homologues and their isoforms from *Solanum lycopersicum,* File S1: The R scripts and utility files used in this work, File S2: The full results of the MEME search of the 4886 MLO protein sequences, Figure S1: The neighbour-joining tree of the 4886 sequences of MLO proteins with extended clades, Figure S2: Multiple sequence alignment of the 4886 MLO proteins and the neighbour-joining tree, Figure S3: Multiple sequence alignments of the MLO protein sequences from the Solanaceae species by clade.
