# Supplementary figures and images for: MLO Proteins from Tomato (Solanum lycopersicum L.) and Related Species in the Broad Phylogenetic Context (part 2 of 2)
Source: Plants (Basel). 2022 Jun 16;11(12):1588. doi: 10.3390/plants11121588 (PMC9229925; doi:10.3390/plants11121588)

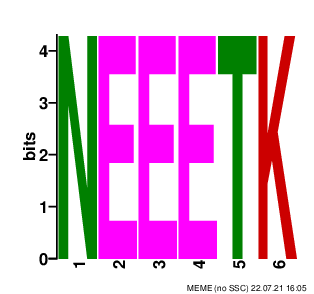

Supplement: Supplementary file 1 [file plants-11-01588-s001.zip › Supplementary File S2/meme200/logo181.png]

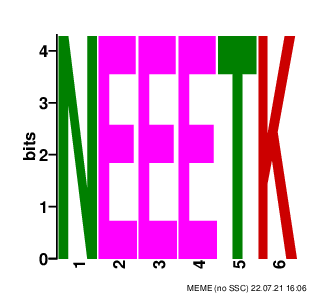

Supplement: Supplementary file 1 [file plants-11-01588-s001.zip › Supplementary File S2/meme200/logo182.png]

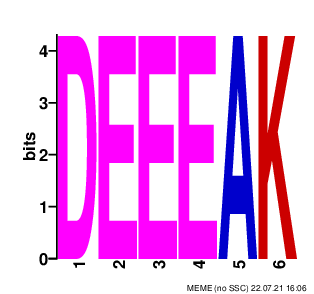

Supplement: Supplementary file 1 [file plants-11-01588-s001.zip › Supplementary File S2/meme200/logo183.png]

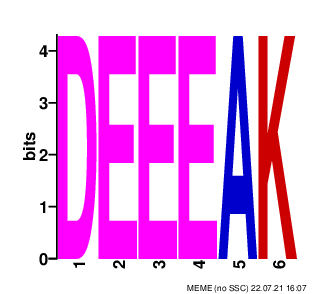

Supplement: Supplementary file 1 [file plants-11-01588-s001.zip › Supplementary File S2/meme200/logo184.png]

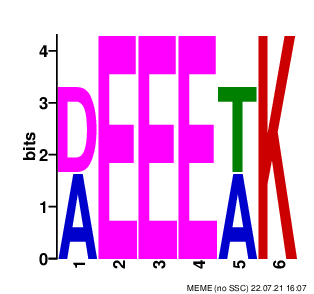

Supplement: Supplementary file 1 [file plants-11-01588-s001.zip › Supplementary File S2/meme200/logo185.png]

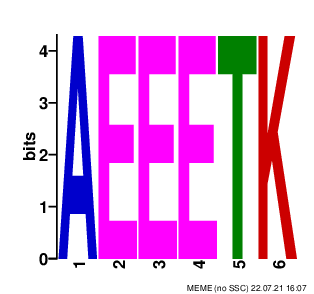

Supplement: Supplementary file 1 [file plants-11-01588-s001.zip › Supplementary File S2/meme200/logo186.png]

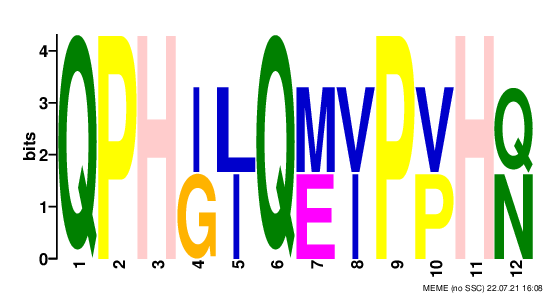

Supplement: Supplementary file 1 [file plants-11-01588-s001.zip › Supplementary File S2/meme200/logo187.png]

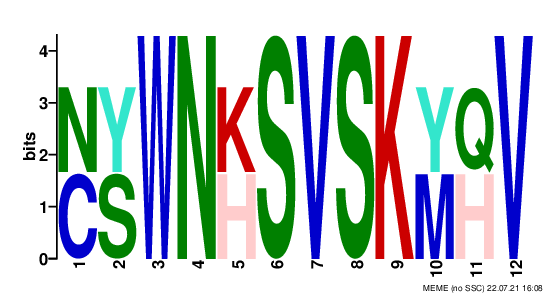

Supplement: Supplementary file 1 [file plants-11-01588-s001.zip › Supplementary File S2/meme200/logo188.png]

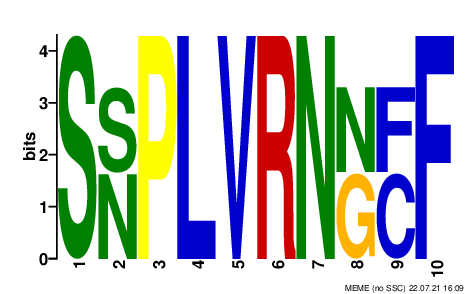

Supplement: Supplementary file 1 [file plants-11-01588-s001.zip › Supplementary File S2/meme200/logo189.png]

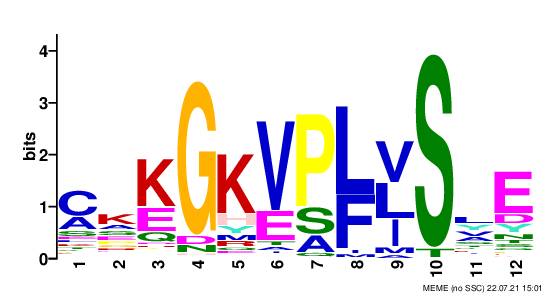

Supplement: Supplementary file 1 [file plants-11-01588-s001.zip › Supplementary File S2/meme200/logo19.png]

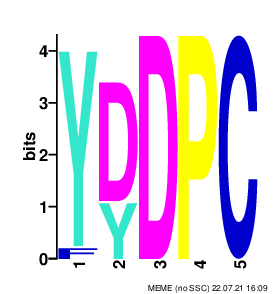

Supplement: Supplementary file 1 [file plants-11-01588-s001.zip › Supplementary File S2/meme200/logo190.png]

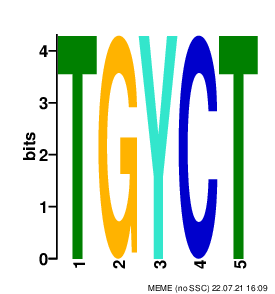

Supplement: Supplementary file 1 [file plants-11-01588-s001.zip › Supplementary File S2/meme200/logo191.png]

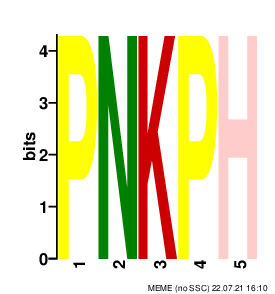

Supplement: Supplementary file 1 [file plants-11-01588-s001.zip › Supplementary File S2/meme200/logo192.png]

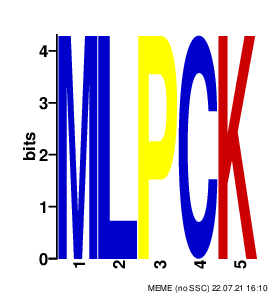

Supplement: Supplementary file 1 [file plants-11-01588-s001.zip › Supplementary File S2/meme200/logo193.png]

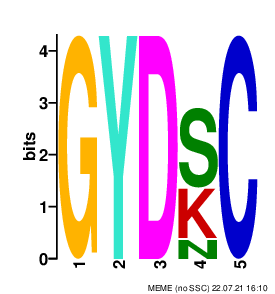

Supplement: Supplementary file 1 [file plants-11-01588-s001.zip › Supplementary File S2/meme200/logo194.png]

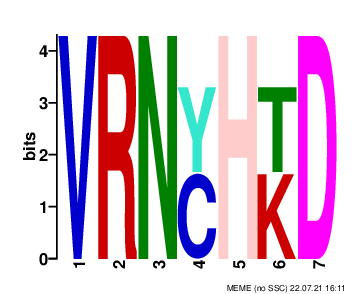

Supplement: Supplementary file 1 [file plants-11-01588-s001.zip › Supplementary File S2/meme200/logo195.png]

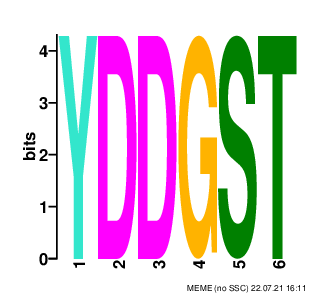

Supplement: Supplementary file 1 [file plants-11-01588-s001.zip › Supplementary File S2/meme200/logo196.png]

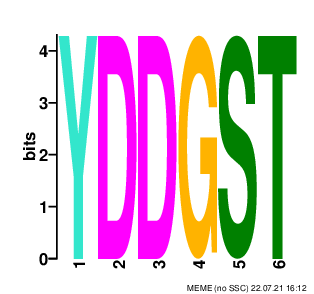

Supplement: Supplementary file 1 [file plants-11-01588-s001.zip › Supplementary File S2/meme200/logo197.png]

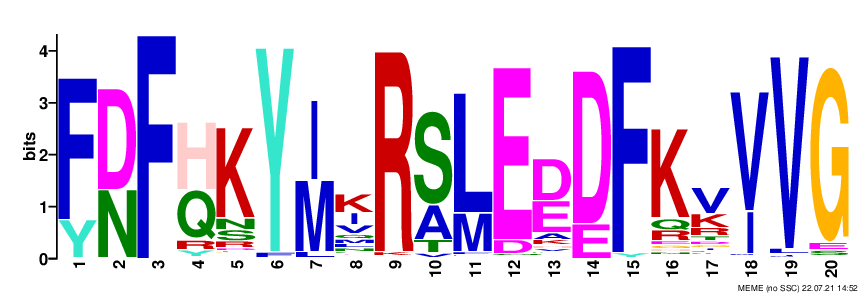

Supplement: Supplementary file 1 [file plants-11-01588-s001.zip › Supplementary File S2/meme200/logo2.png]

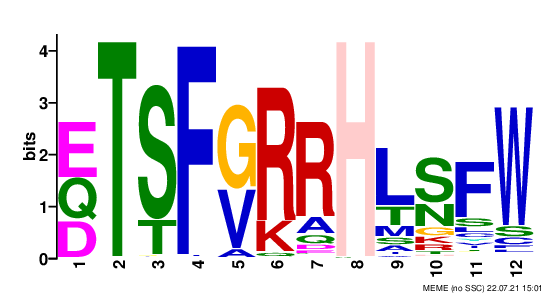

Supplement: Supplementary file 1 [file plants-11-01588-s001.zip › Supplementary File S2/meme200/logo20.png]

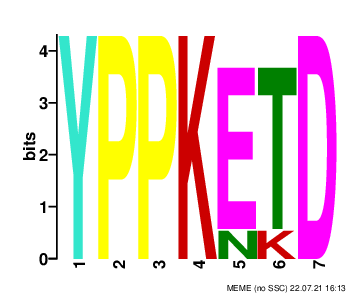

Supplement: Supplementary file 1 [file plants-11-01588-s001.zip › Supplementary File S2/meme200/logo200.png]

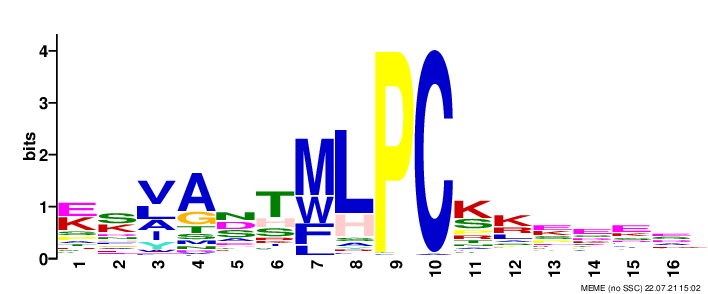

Supplement: Supplementary file 1 [file plants-11-01588-s001.zip › Supplementary File S2/meme200/logo21.png]

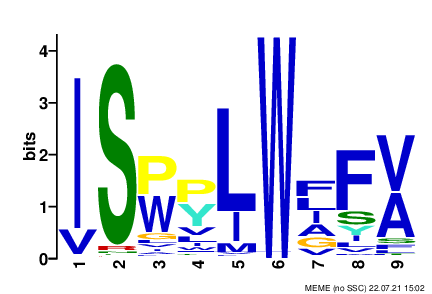

Supplement: Supplementary file 1 [file plants-11-01588-s001.zip › Supplementary File S2/meme200/logo22.png]

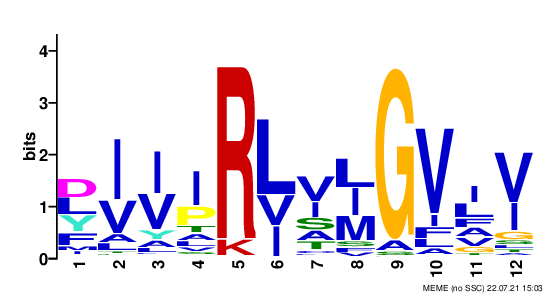

Supplement: Supplementary file 1 [file plants-11-01588-s001.zip › Supplementary File S2/meme200/logo23.png]

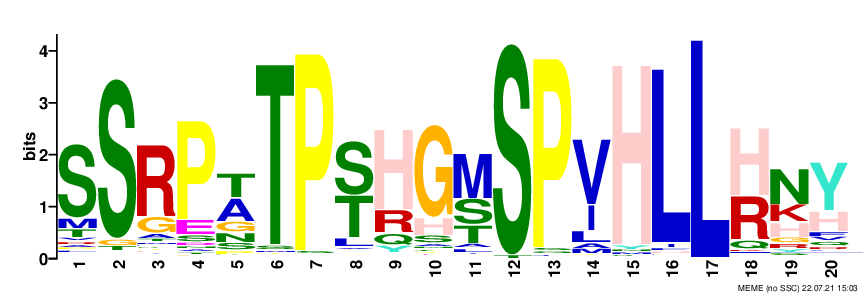

Supplement: Supplementary file 1 [file plants-11-01588-s001.zip › Supplementary File S2/meme200/logo24.png]

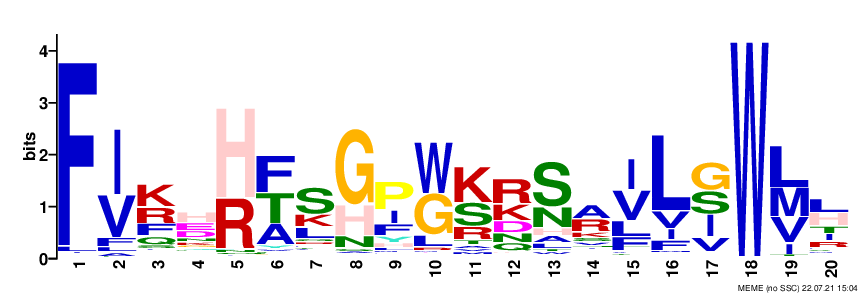

Supplement: Supplementary file 1 [file plants-11-01588-s001.zip › Supplementary File S2/meme200/logo25.png]

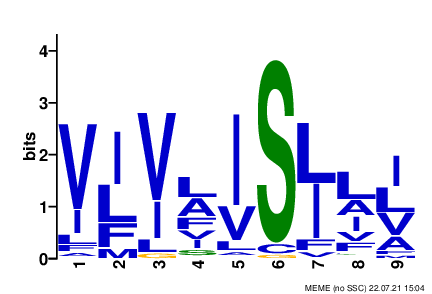

Supplement: Supplementary file 1 [file plants-11-01588-s001.zip › Supplementary File S2/meme200/logo26.png]

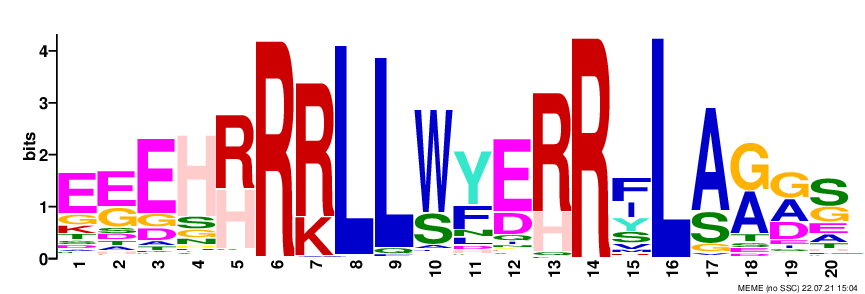

Supplement: Supplementary file 1 [file plants-11-01588-s001.zip › Supplementary File S2/meme200/logo27.png]

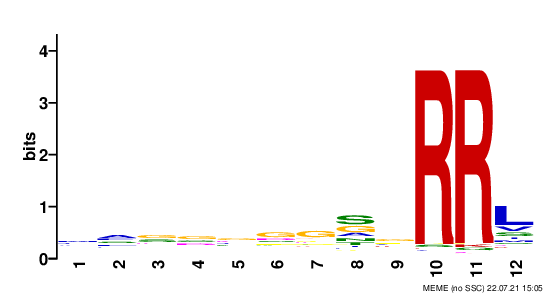

Supplement: Supplementary file 1 [file plants-11-01588-s001.zip › Supplementary File S2/meme200/logo28.png]

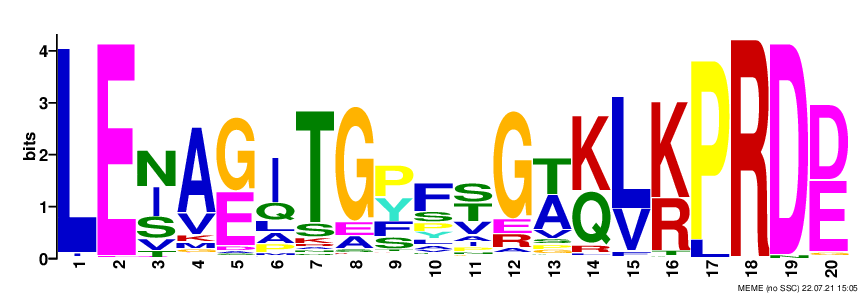

Supplement: Supplementary file 1 [file plants-11-01588-s001.zip › Supplementary File S2/meme200/logo29.png]

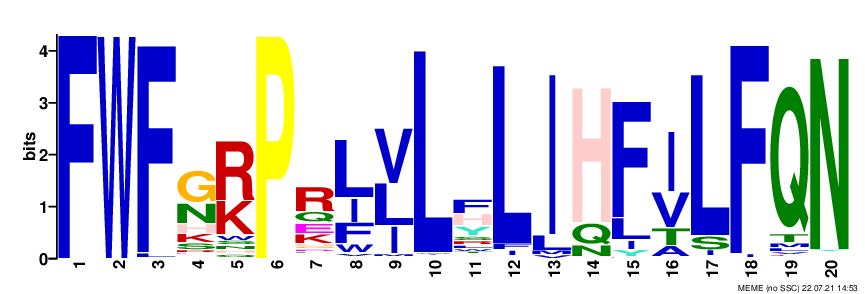

Supplement: Supplementary file 1 [file plants-11-01588-s001.zip › Supplementary File S2/meme200/logo3.png]

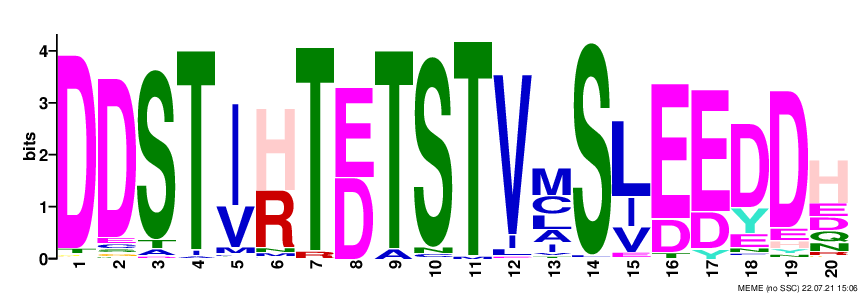

Supplement: Supplementary file 1 [file plants-11-01588-s001.zip › Supplementary File S2/meme200/logo30.png]

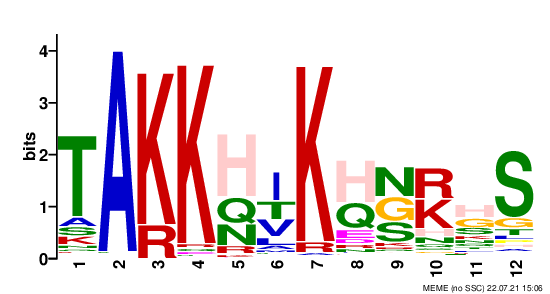

Supplement: Supplementary file 1 [file plants-11-01588-s001.zip › Supplementary File S2/meme200/logo31.png]

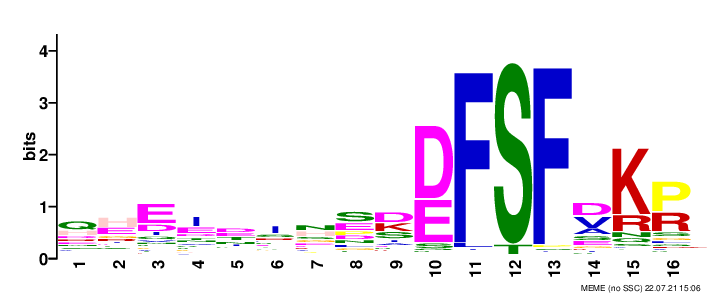

Supplement: Supplementary file 1 [file plants-11-01588-s001.zip › Supplementary File S2/meme200/logo32.png]

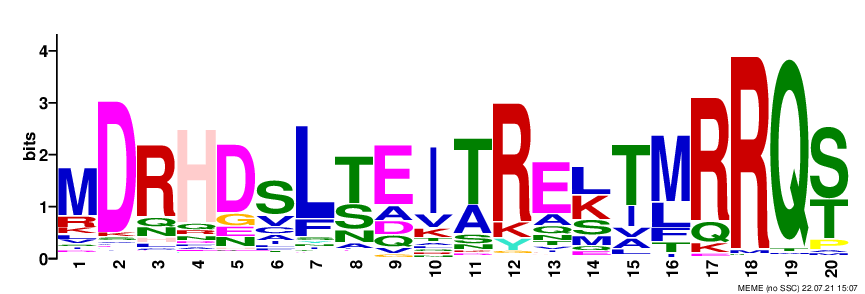

Supplement: Supplementary file 1 [file plants-11-01588-s001.zip › Supplementary File S2/meme200/logo33.png]

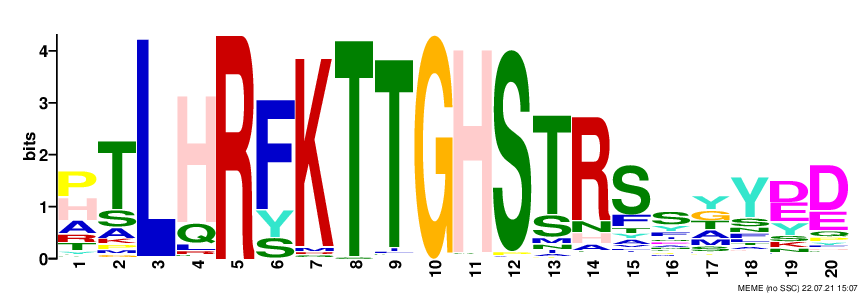

Supplement: Supplementary file 1 [file plants-11-01588-s001.zip › Supplementary File S2/meme200/logo34.png]

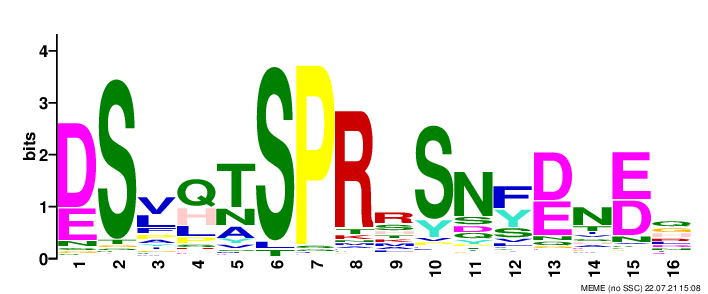

Supplement: Supplementary file 1 [file plants-11-01588-s001.zip › Supplementary File S2/meme200/logo35.png]

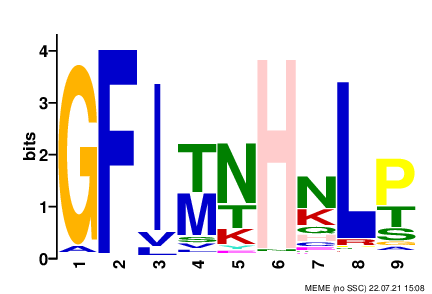

Supplement: Supplementary file 1 [file plants-11-01588-s001.zip › Supplementary File S2/meme200/logo36.png]

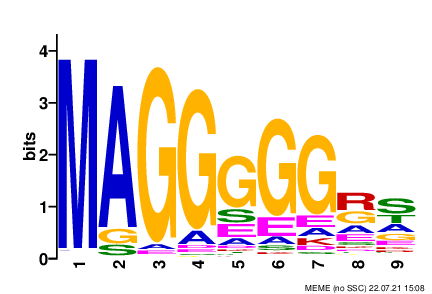

Supplement: Supplementary file 1 [file plants-11-01588-s001.zip › Supplementary File S2/meme200/logo37.png]

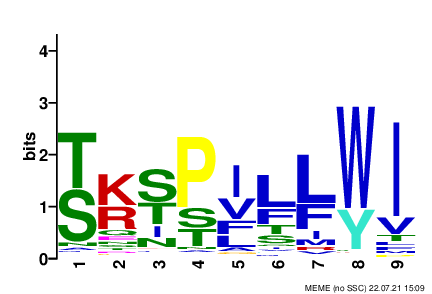

Supplement: Supplementary file 1 [file plants-11-01588-s001.zip › Supplementary File S2/meme200/logo38.png]

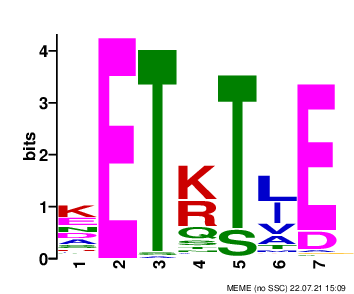

Supplement: Supplementary file 1 [file plants-11-01588-s001.zip › Supplementary File S2/meme200/logo39.png]

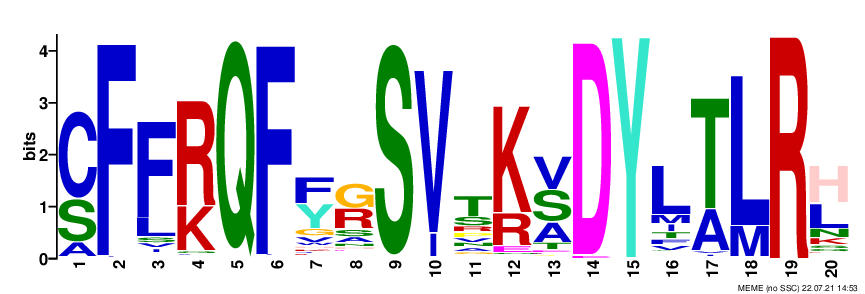

Supplement: Supplementary file 1 [file plants-11-01588-s001.zip › Supplementary File S2/meme200/logo4.png]

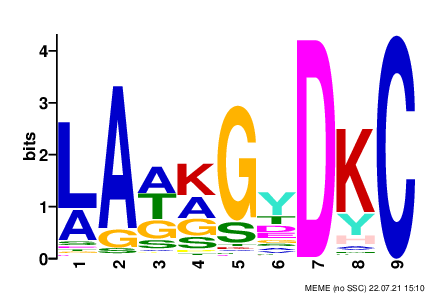

Supplement: Supplementary file 1 [file plants-11-01588-s001.zip › Supplementary File S2/meme200/logo40.png]

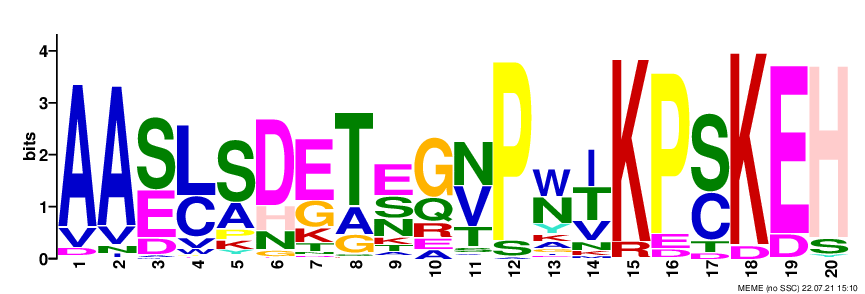

Supplement: Supplementary file 1 [file plants-11-01588-s001.zip › Supplementary File S2/meme200/logo41.png]

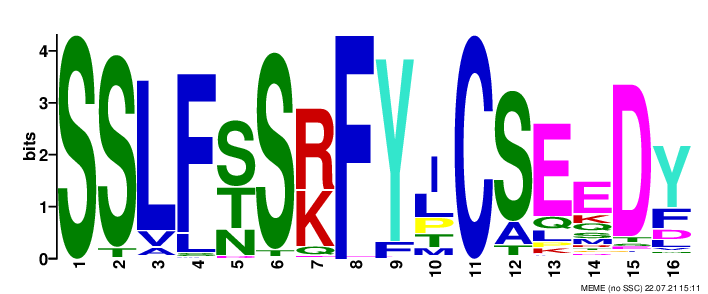

Supplement: Supplementary file 1 [file plants-11-01588-s001.zip › Supplementary File S2/meme200/logo42.png]

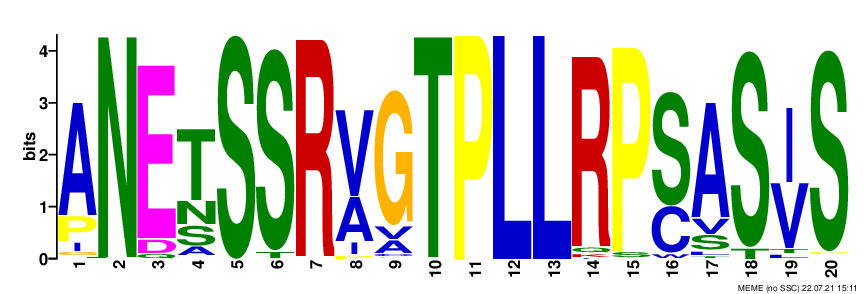

Supplement: Supplementary file 1 [file plants-11-01588-s001.zip › Supplementary File S2/meme200/logo43.png]

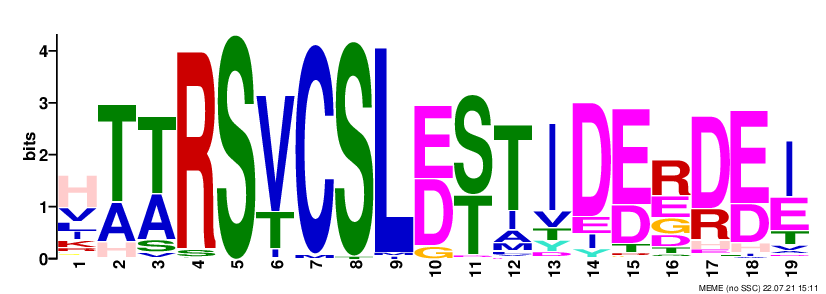

Supplement: Supplementary file 1 [file plants-11-01588-s001.zip › Supplementary File S2/meme200/logo44.png]

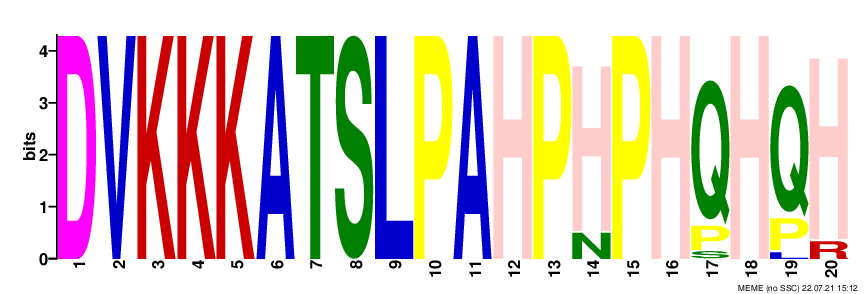

Supplement: Supplementary file 1 [file plants-11-01588-s001.zip › Supplementary File S2/meme200/logo45.png]

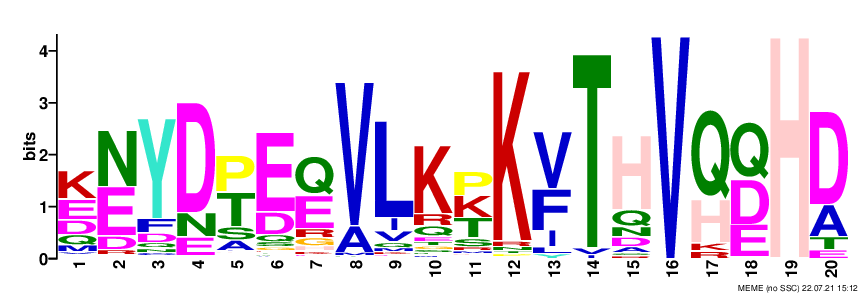

Supplement: Supplementary file 1 [file plants-11-01588-s001.zip › Supplementary File S2/meme200/logo46.png]

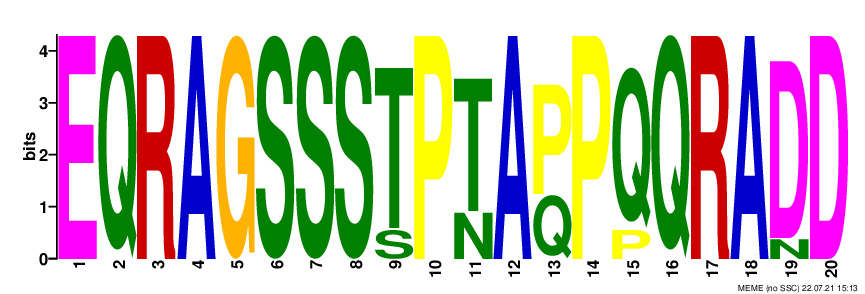

Supplement: Supplementary file 1 [file plants-11-01588-s001.zip › Supplementary File S2/meme200/logo47.png]

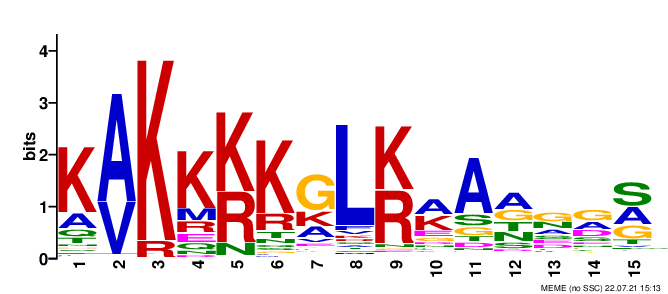

Supplement: Supplementary file 1 [file plants-11-01588-s001.zip › Supplementary File S2/meme200/logo48.png]

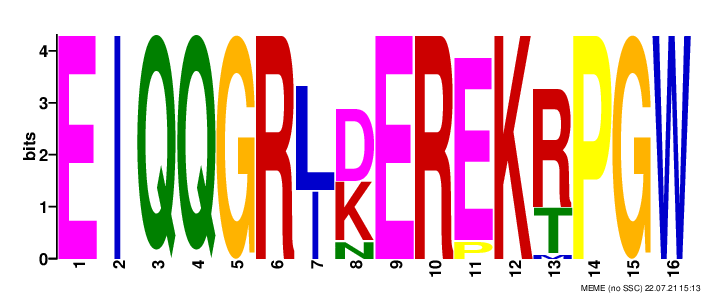

Supplement: Supplementary file 1 [file plants-11-01588-s001.zip › Supplementary File S2/meme200/logo49.png]

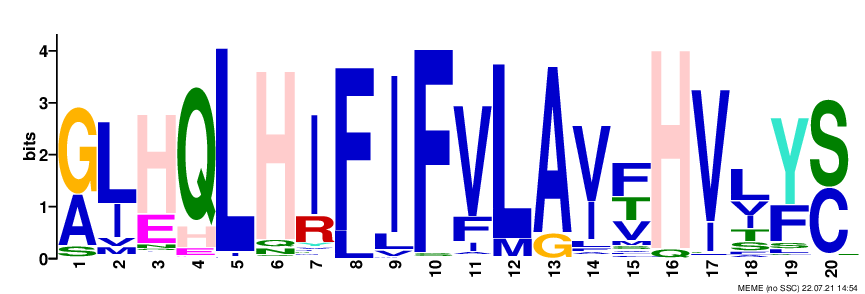

Supplement: Supplementary file 1 [file plants-11-01588-s001.zip › Supplementary File S2/meme200/logo5.png]

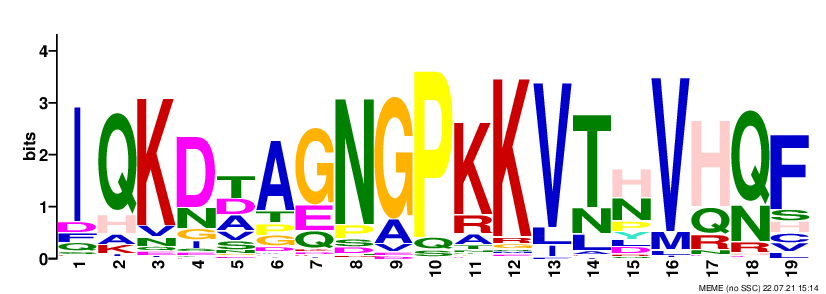

Supplement: Supplementary file 1 [file plants-11-01588-s001.zip › Supplementary File S2/meme200/logo50.png]

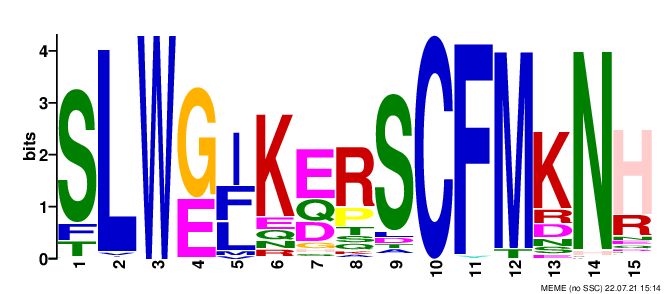

Supplement: Supplementary file 1 [file plants-11-01588-s001.zip › Supplementary File S2/meme200/logo51.png]

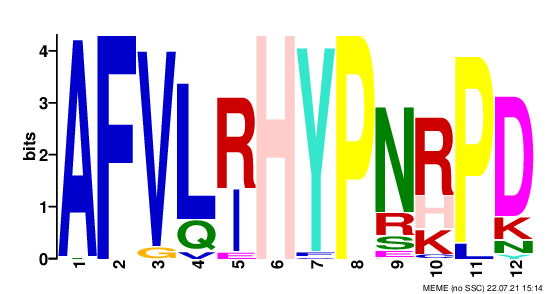

Supplement: Supplementary file 1 [file plants-11-01588-s001.zip › Supplementary File S2/meme200/logo52.png]

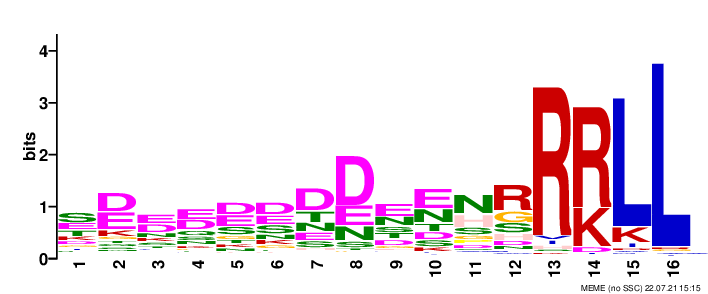

Supplement: Supplementary file 1 [file plants-11-01588-s001.zip › Supplementary File S2/meme200/logo53.png]

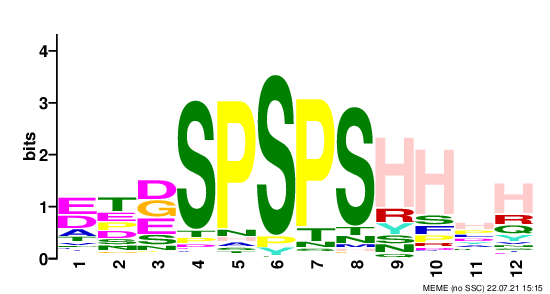

Supplement: Supplementary file 1 [file plants-11-01588-s001.zip › Supplementary File S2/meme200/logo54.png]

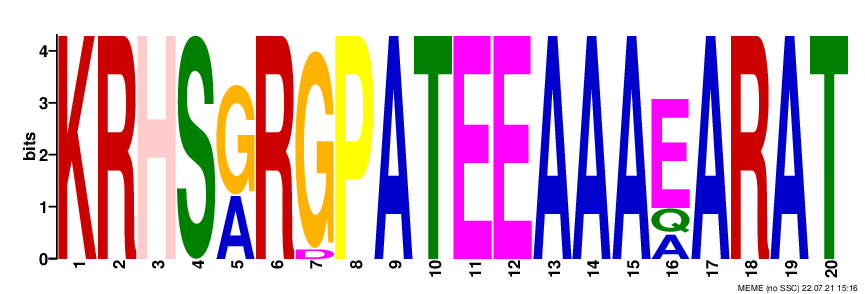

Supplement: Supplementary file 1 [file plants-11-01588-s001.zip › Supplementary File S2/meme200/logo55.png]

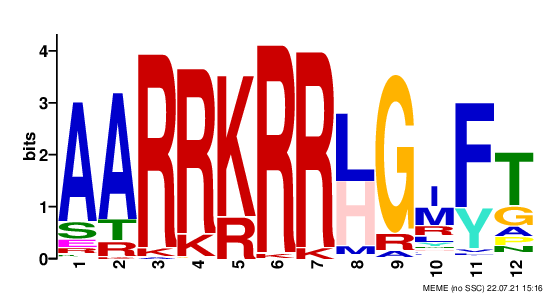

Supplement: Supplementary file 1 [file plants-11-01588-s001.zip › Supplementary File S2/meme200/logo56.png]

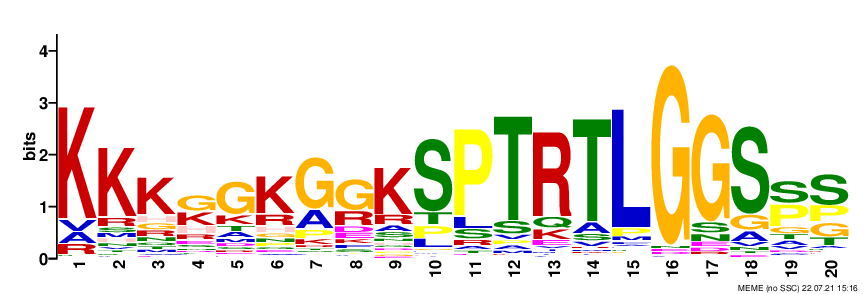

Supplement: Supplementary file 1 [file plants-11-01588-s001.zip › Supplementary File S2/meme200/logo57.png]

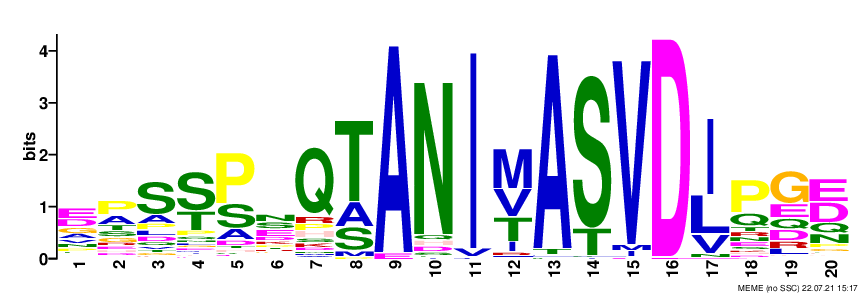

Supplement: Supplementary file 1 [file plants-11-01588-s001.zip › Supplementary File S2/meme200/logo58.png]

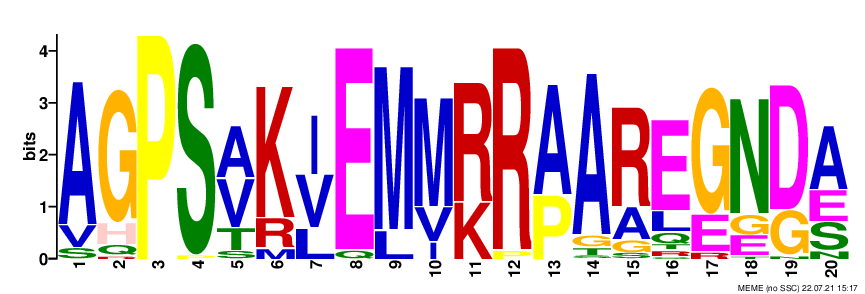

Supplement: Supplementary file 1 [file plants-11-01588-s001.zip › Supplementary File S2/meme200/logo59.png]

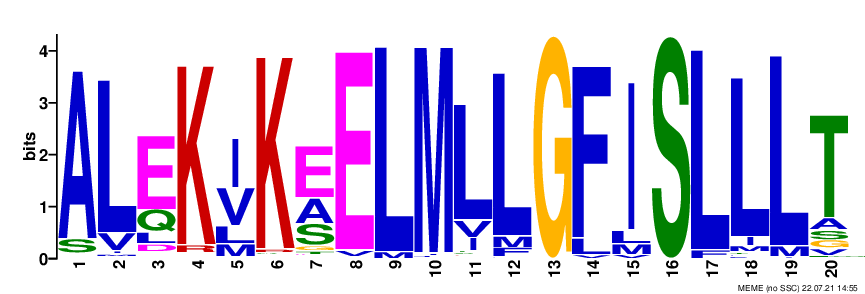

Supplement: Supplementary file 1 [file plants-11-01588-s001.zip › Supplementary File S2/meme200/logo6.png]

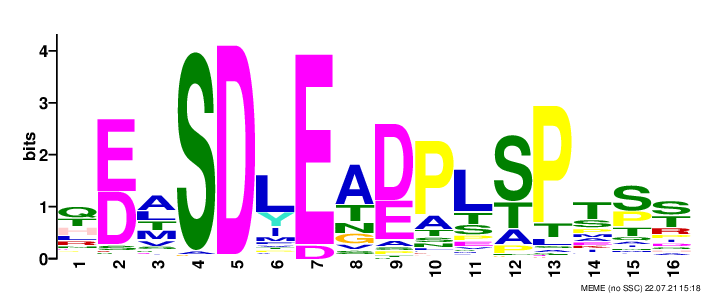

Supplement: Supplementary file 1 [file plants-11-01588-s001.zip › Supplementary File S2/meme200/logo60.png]

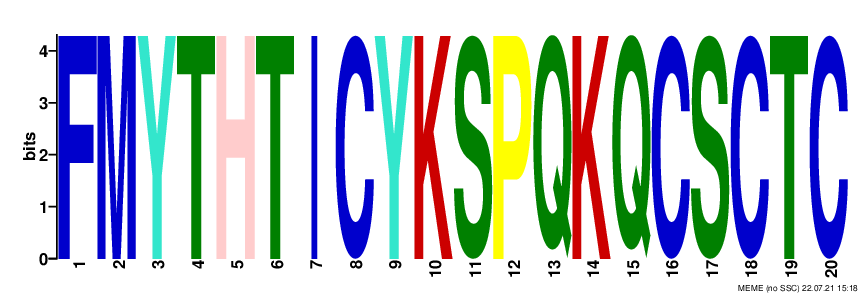

Supplement: Supplementary file 1 [file plants-11-01588-s001.zip › Supplementary File S2/meme200/logo61.png]
