# Supplementary figures and images for: MLO Proteins from Tomato (Solanum lycopersicum L.) and Related Species in the Broad Phylogenetic Context (part 1 of 2)
Source: Plants (Basel). 2022 Jun 16;11(12):1588. doi: 10.3390/plants11121588 (PMC9229925; doi:10.3390/plants11121588)

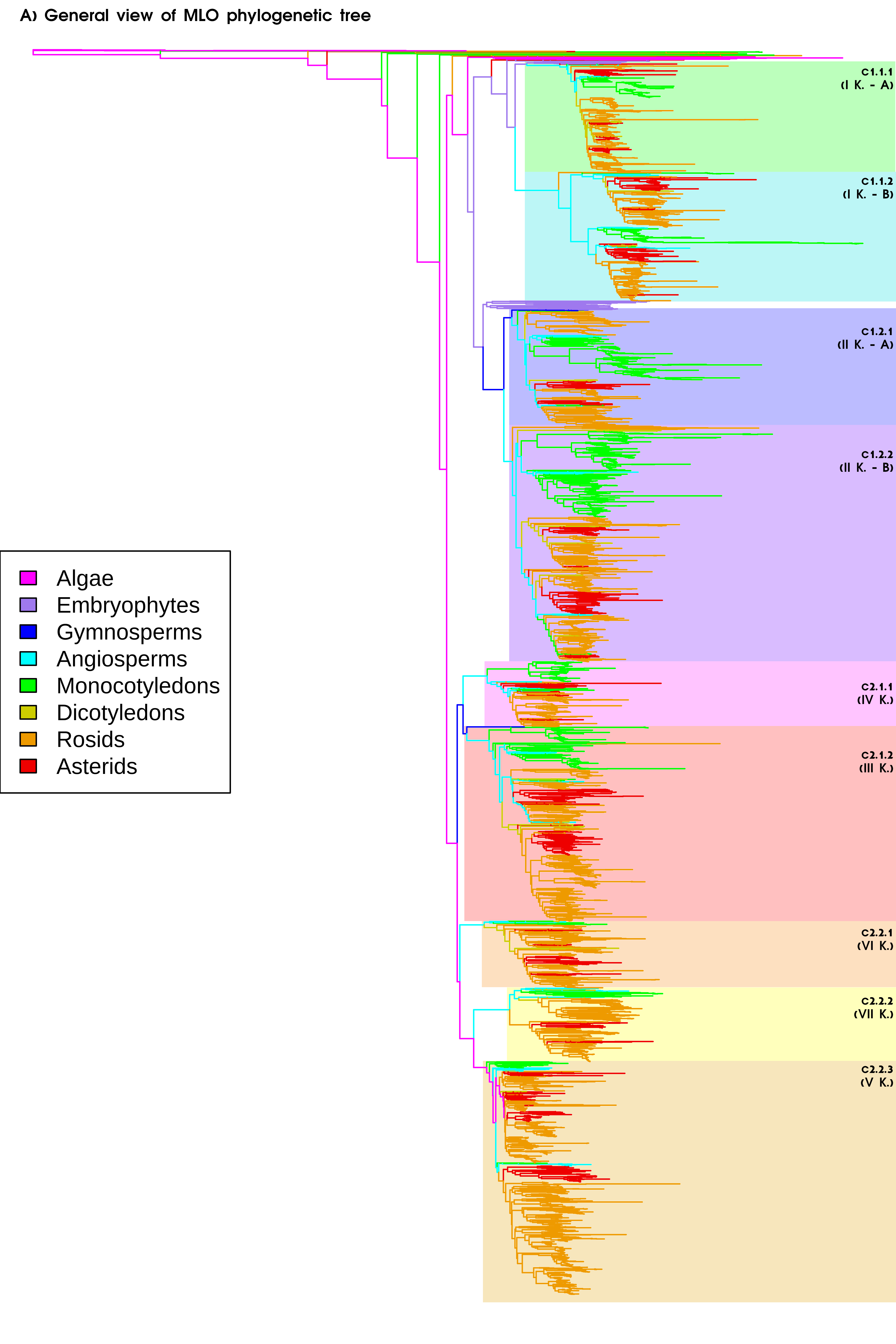

Supplement: Supplementary file 1 [file plants-11-01588-s001.zip › Supplementary Figure S1/Figure S1A.png]

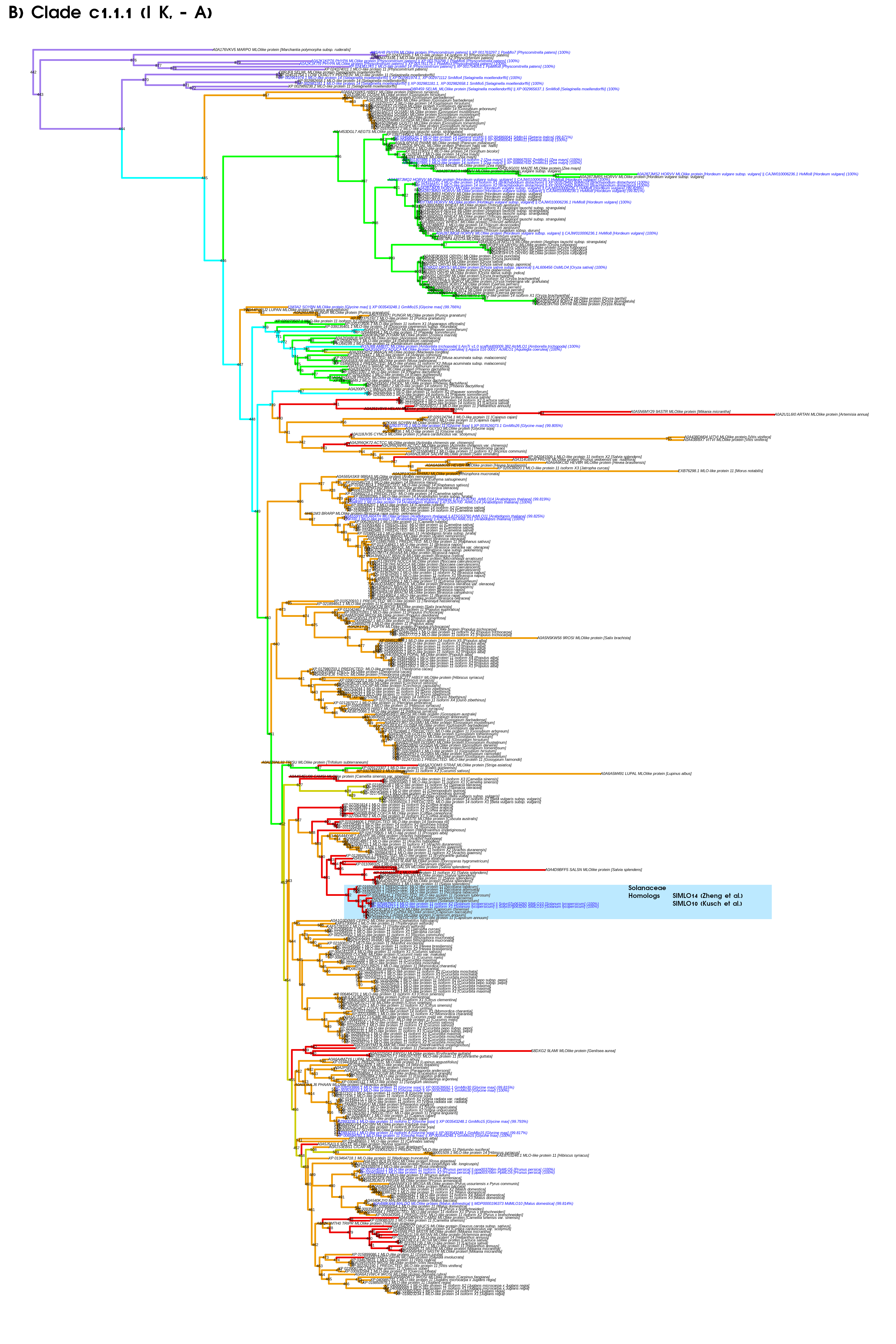

Supplement: Supplementary file 1 [file plants-11-01588-s001.zip › Supplementary Figure S1/Figure S1B.png]

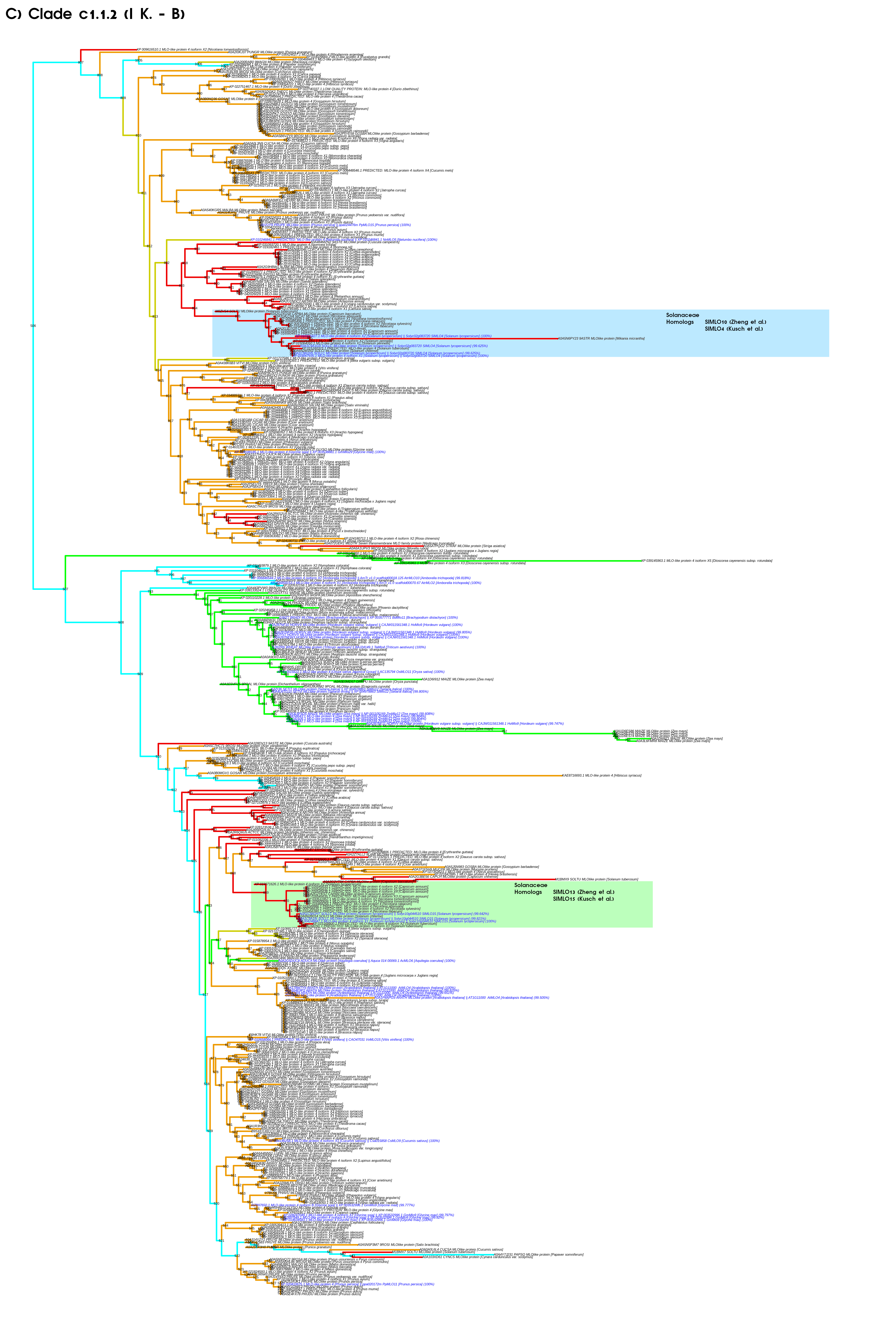

Supplement: Supplementary file 1 [file plants-11-01588-s001.zip › Supplementary Figure S1/Figure S1C.png]

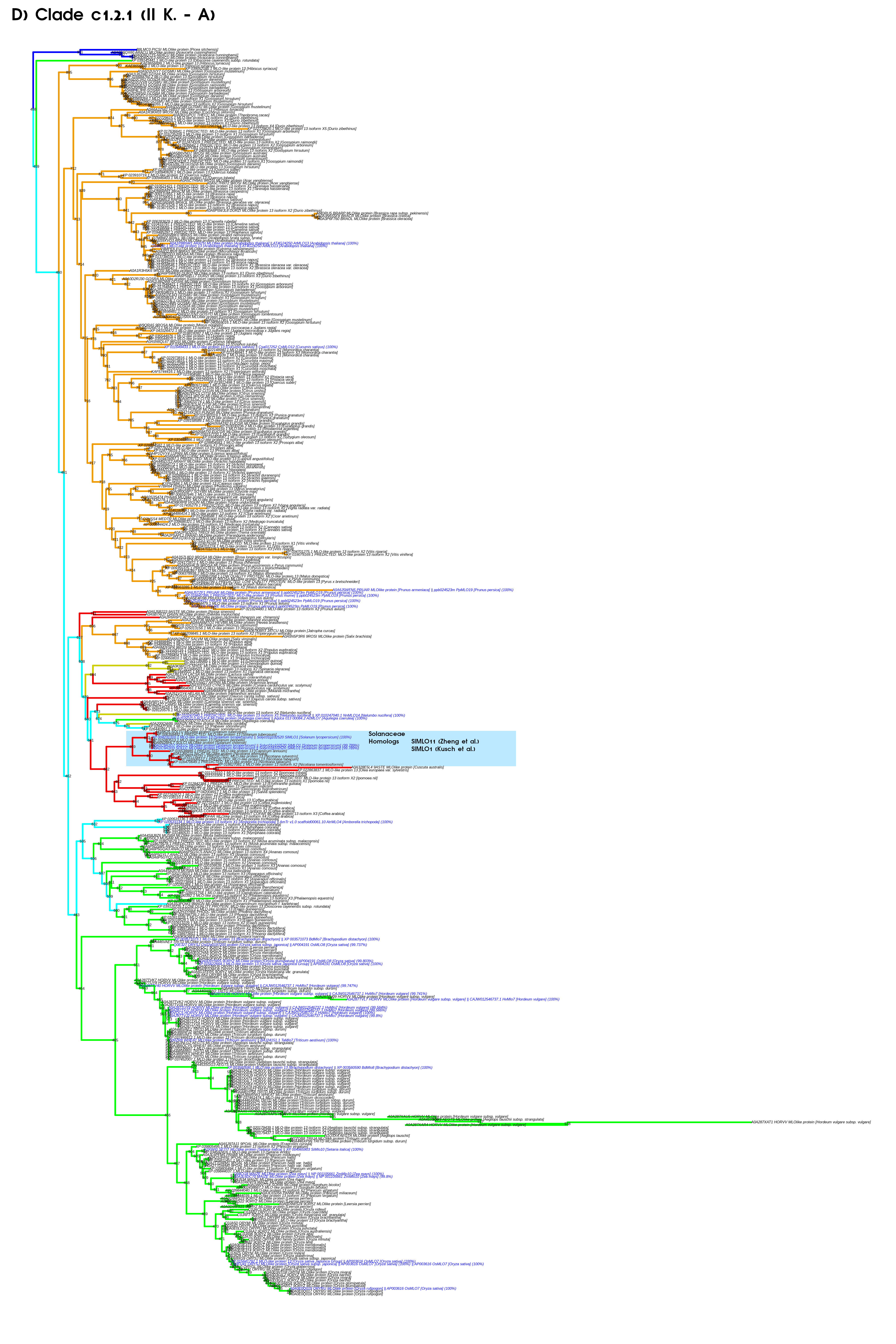

Supplement: Supplementary file 1 [file plants-11-01588-s001.zip › Supplementary Figure S1/Figure S1D.png]

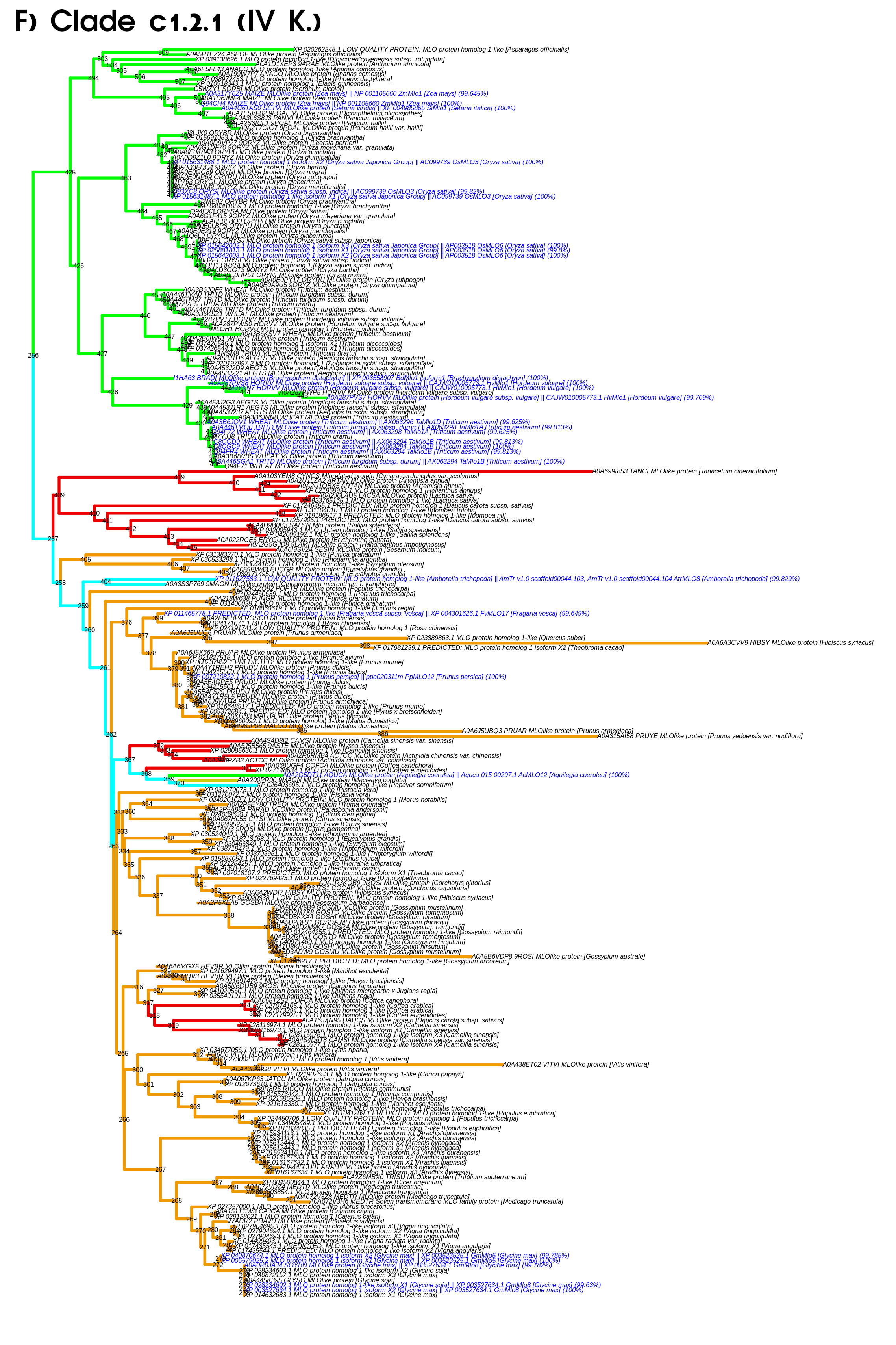

Supplement: Supplementary file 1 [file plants-11-01588-s001.zip › Supplementary Figure S1/Figure S1F.png]

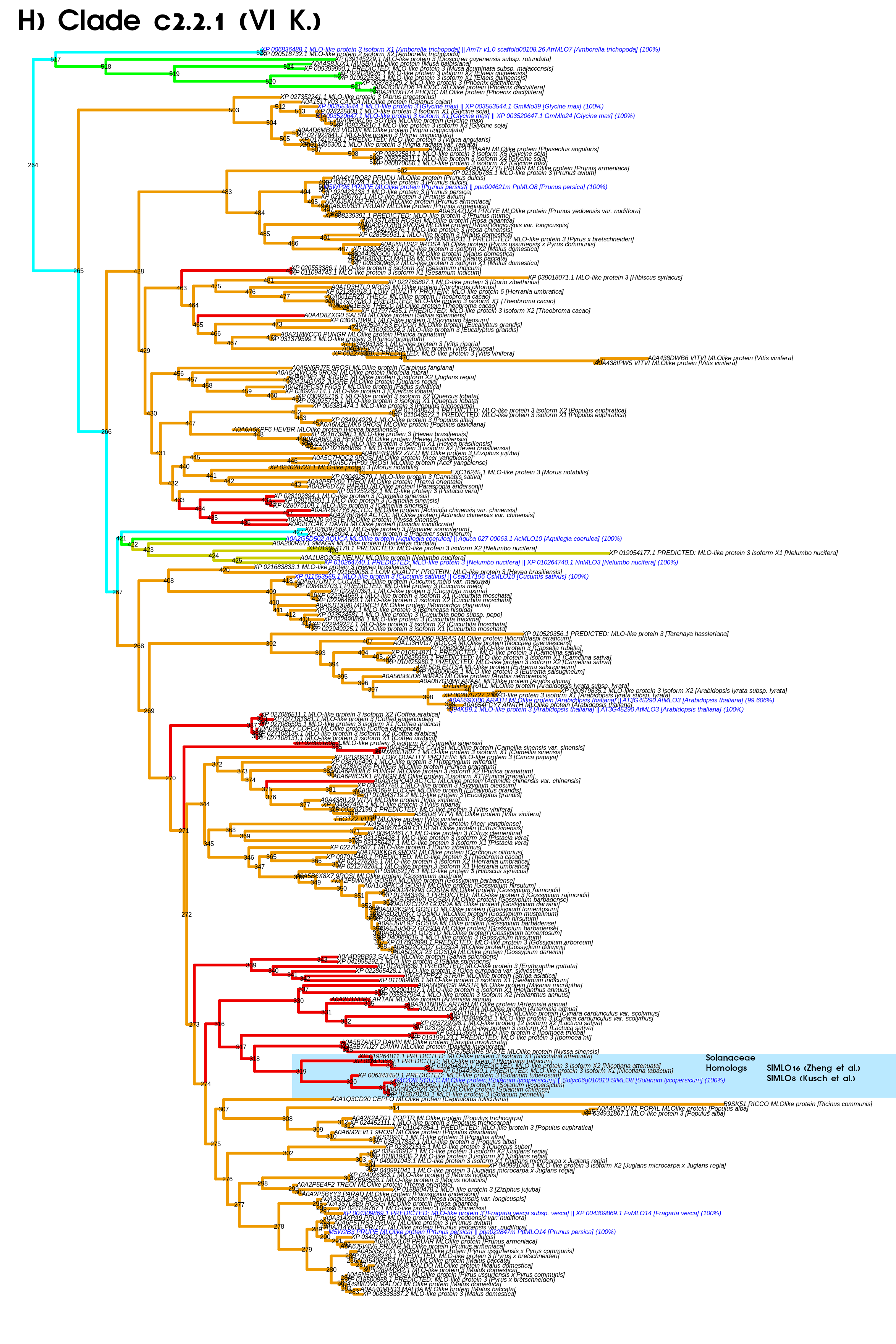

Supplement: Supplementary file 1 [file plants-11-01588-s001.zip › Supplementary Figure S1/Figure S1H.png]

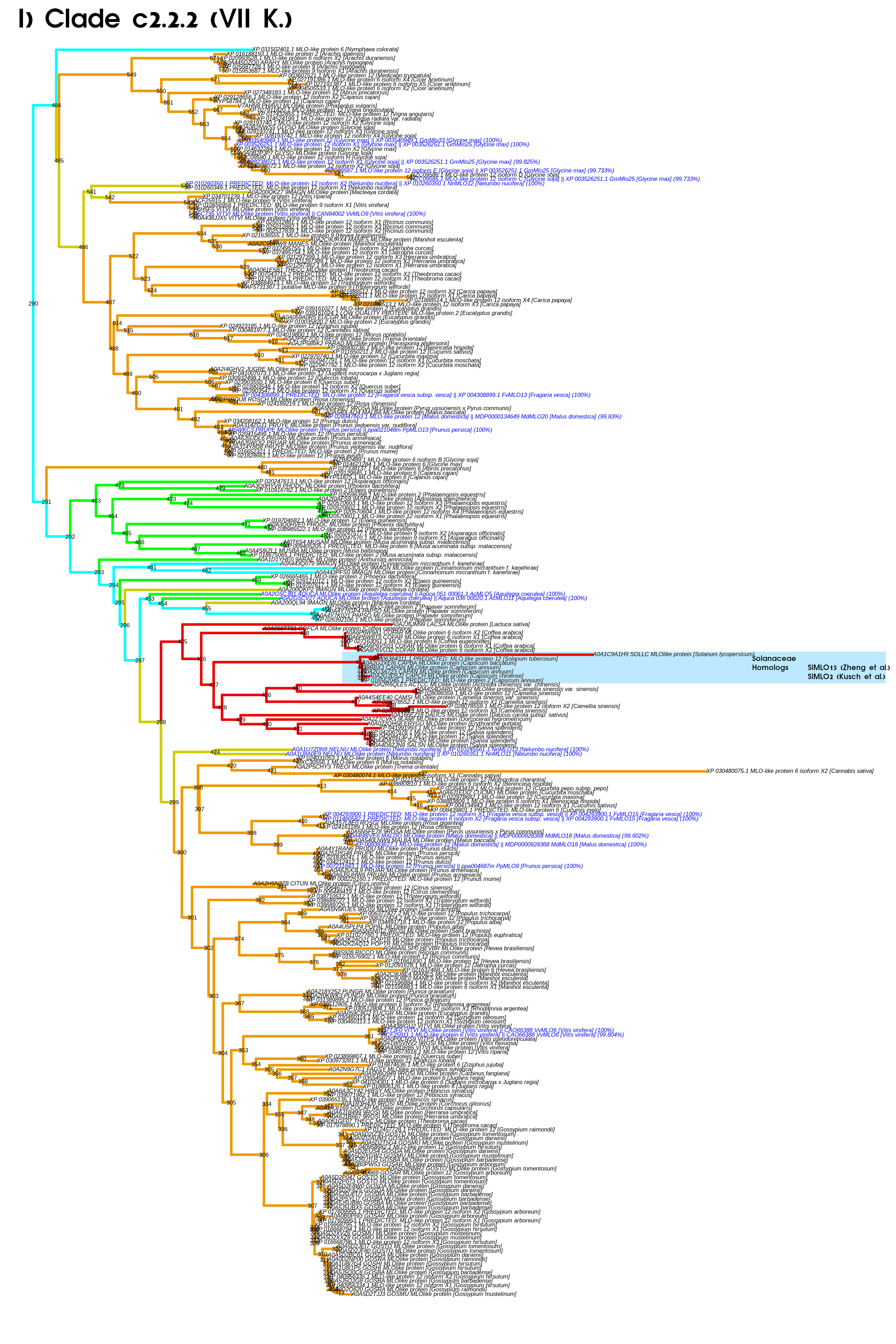

Supplement: Supplementary file 1 [file plants-11-01588-s001.zip › Supplementary Figure S1/Figure S1I.png]

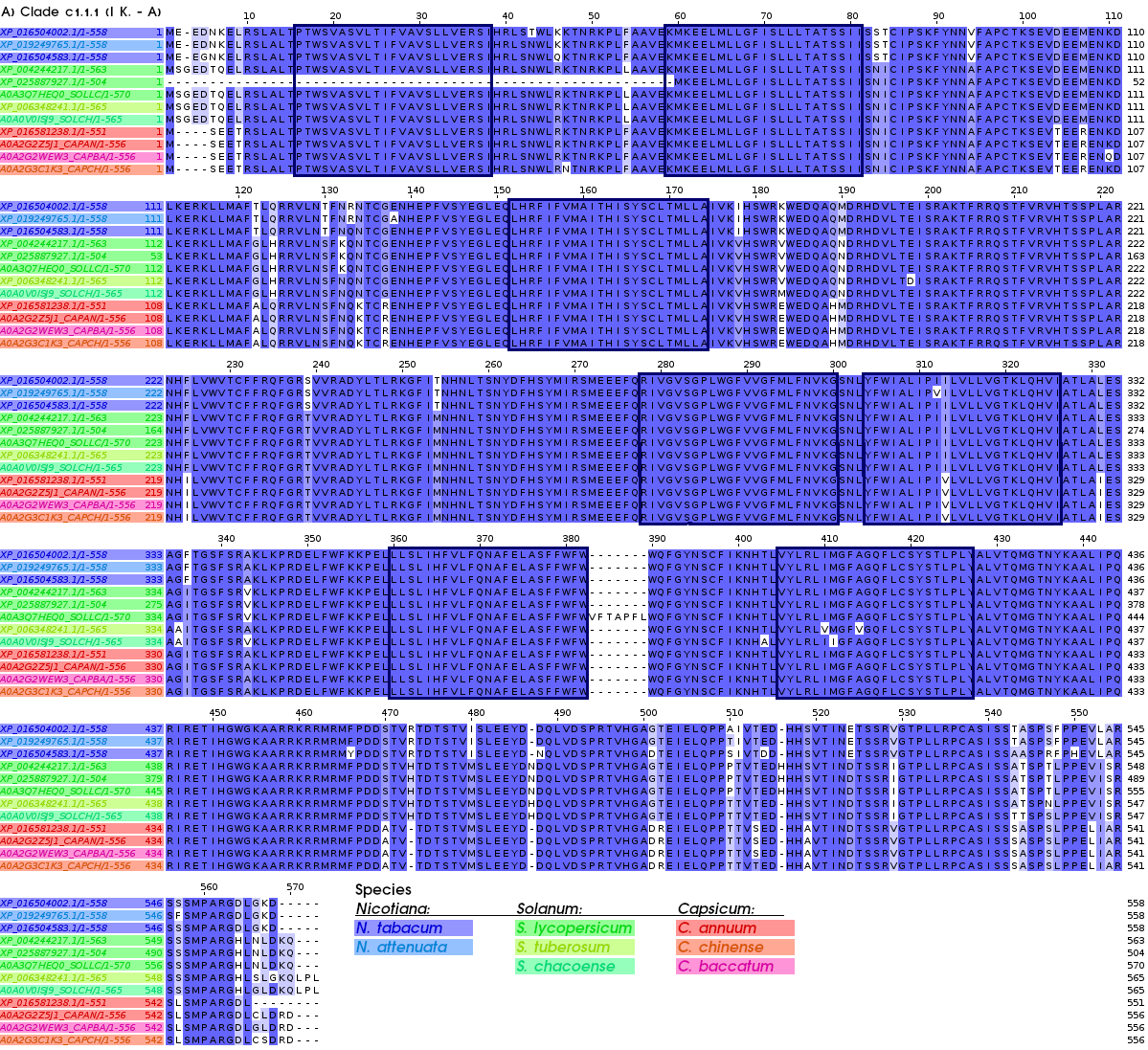

Supplement: Supplementary file 1 [file plants-11-01588-s001.zip › Supplementary Figure S3/Figure S3A.png]

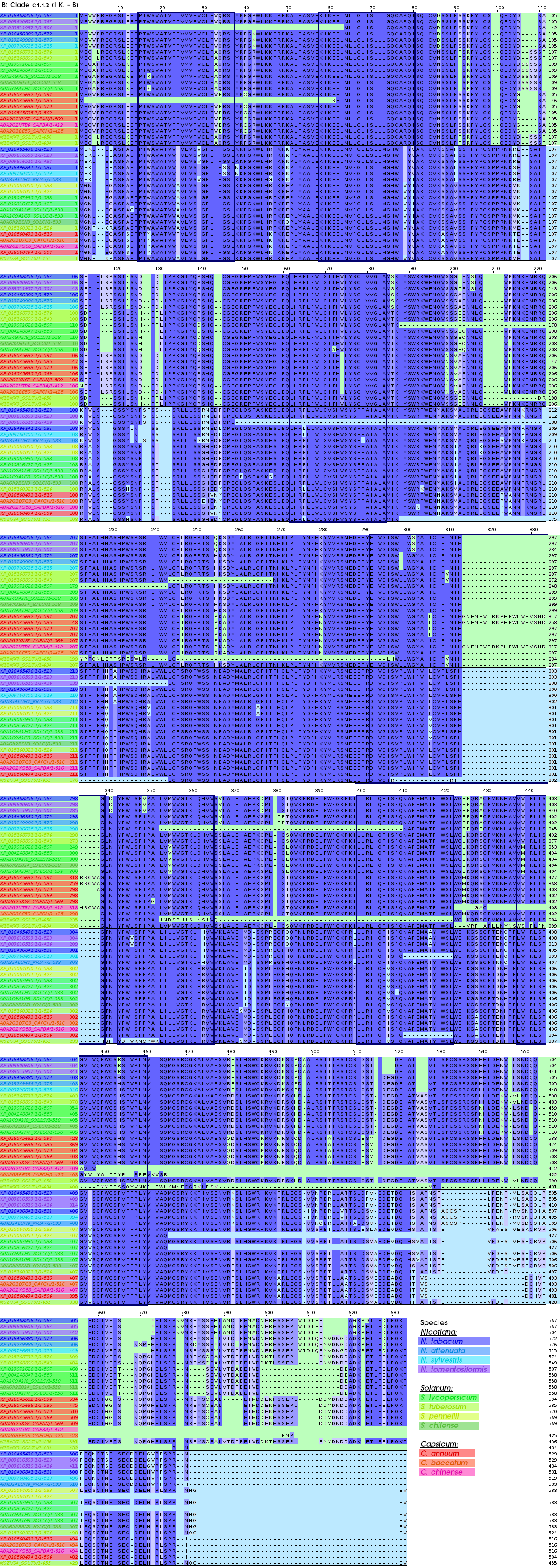

Supplement: Supplementary file 1 [file plants-11-01588-s001.zip › Supplementary Figure S3/Figure S3B.png]

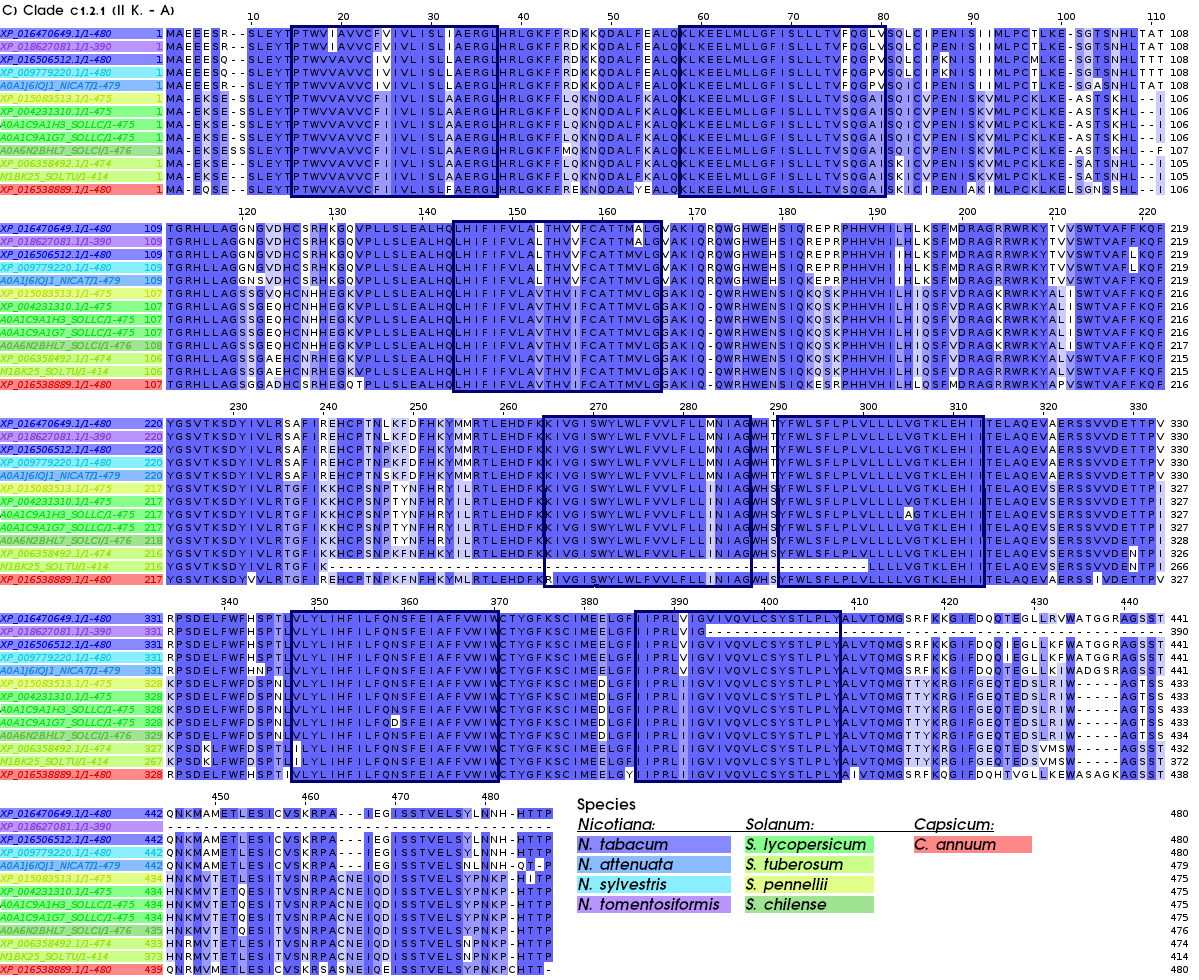

Supplement: Supplementary file 1 [file plants-11-01588-s001.zip › Supplementary Figure S3/Figure S3C.png]

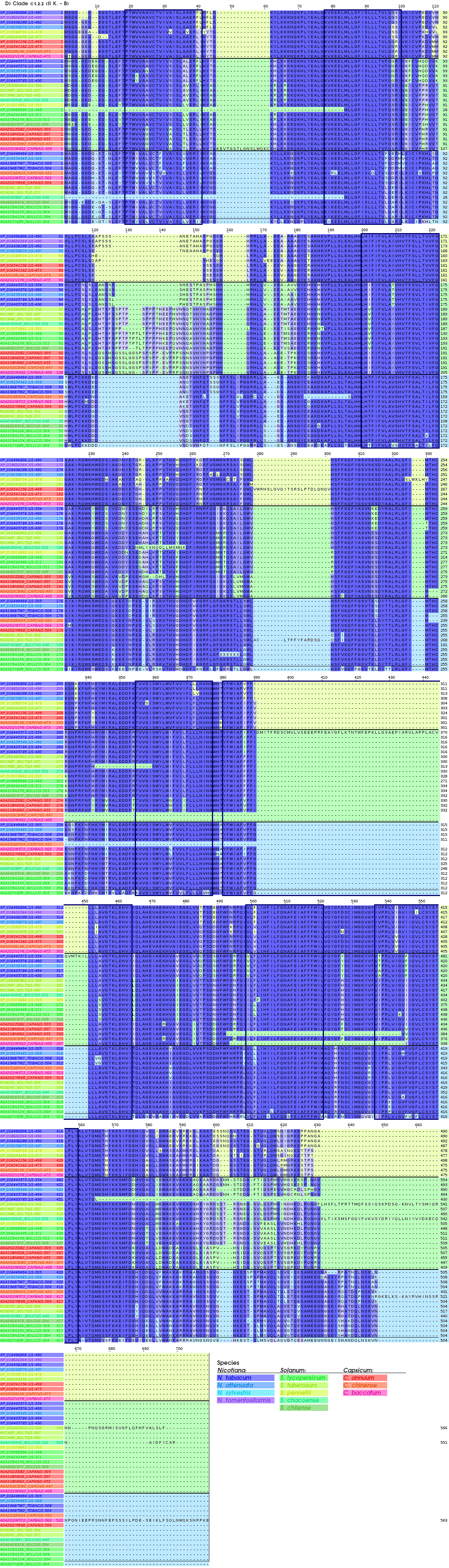

Supplement: Supplementary file 1 [file plants-11-01588-s001.zip › Supplementary Figure S3/Figure S3D.png]

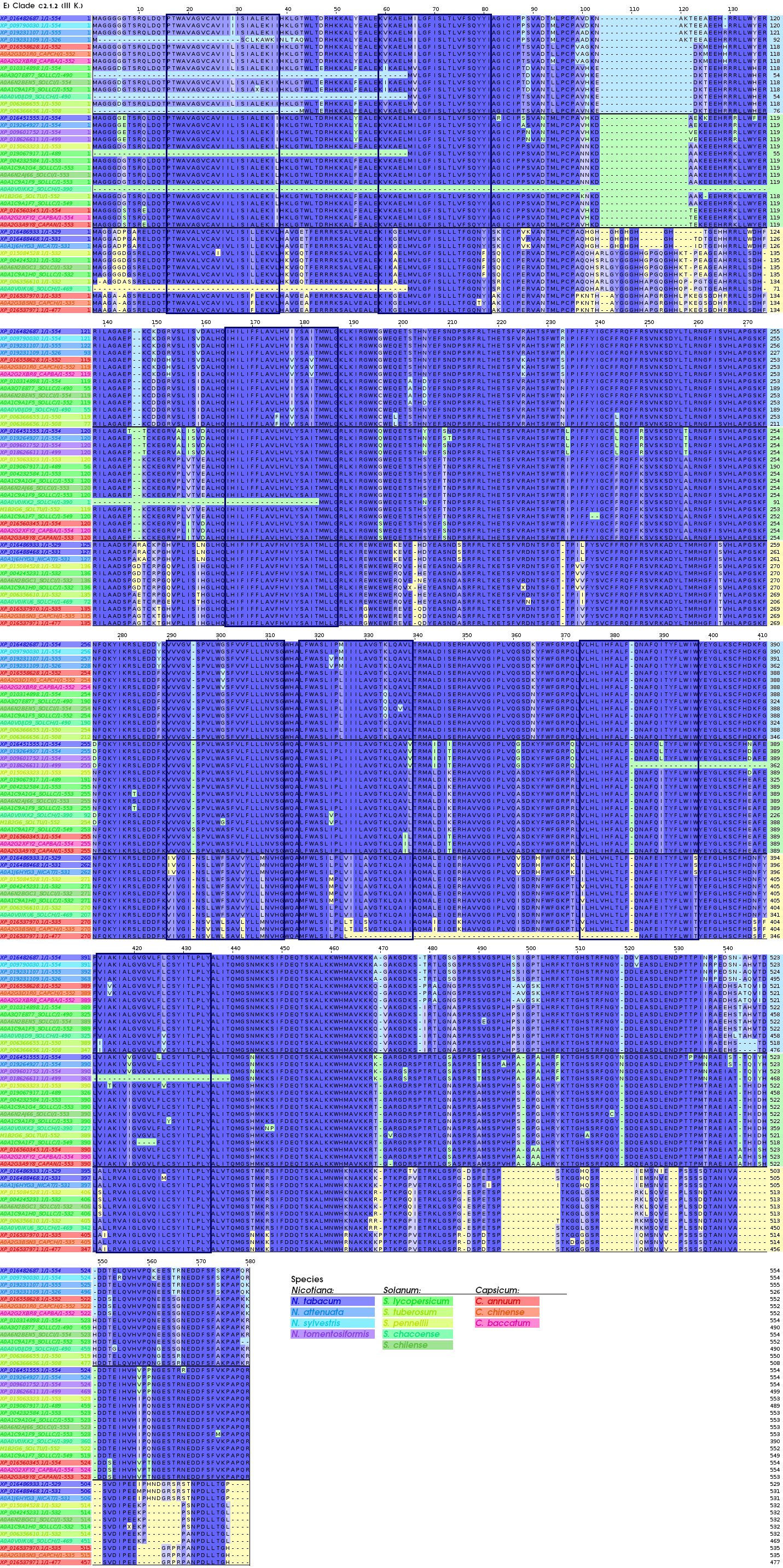

Supplement: Supplementary file 1 [file plants-11-01588-s001.zip › Supplementary Figure S3/Figure S3E.png]

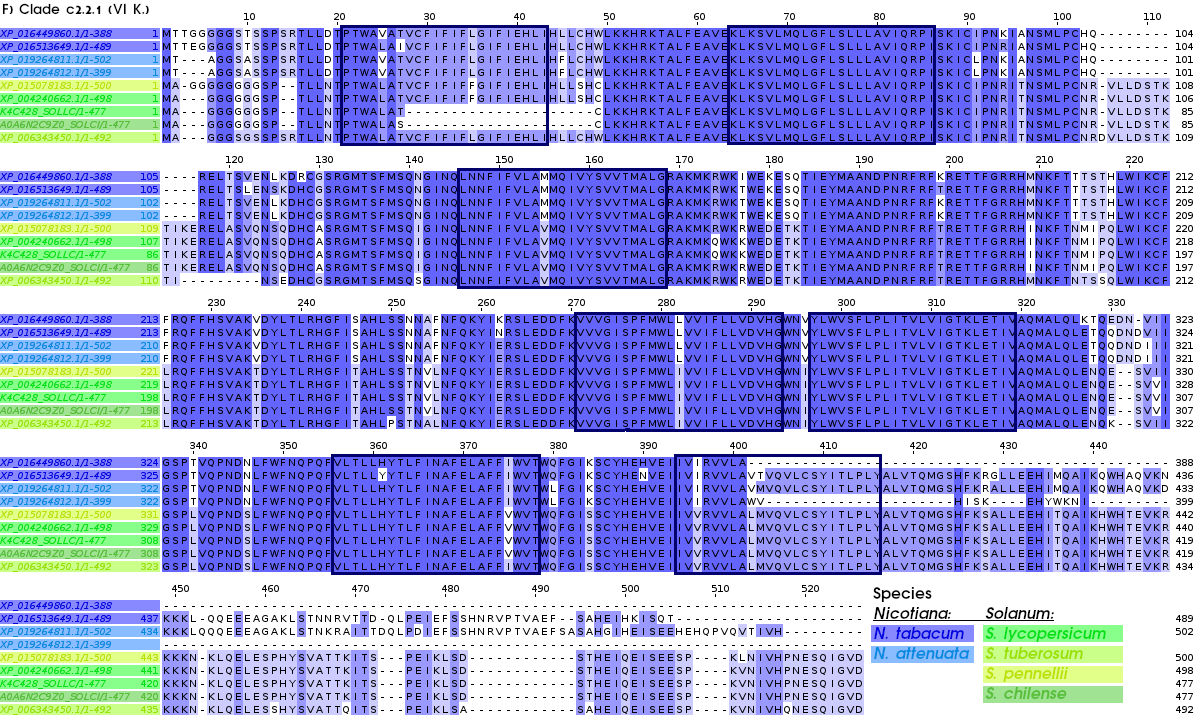

Supplement: Supplementary file 1 [file plants-11-01588-s001.zip › Supplementary Figure S3/Figure S3F.png]

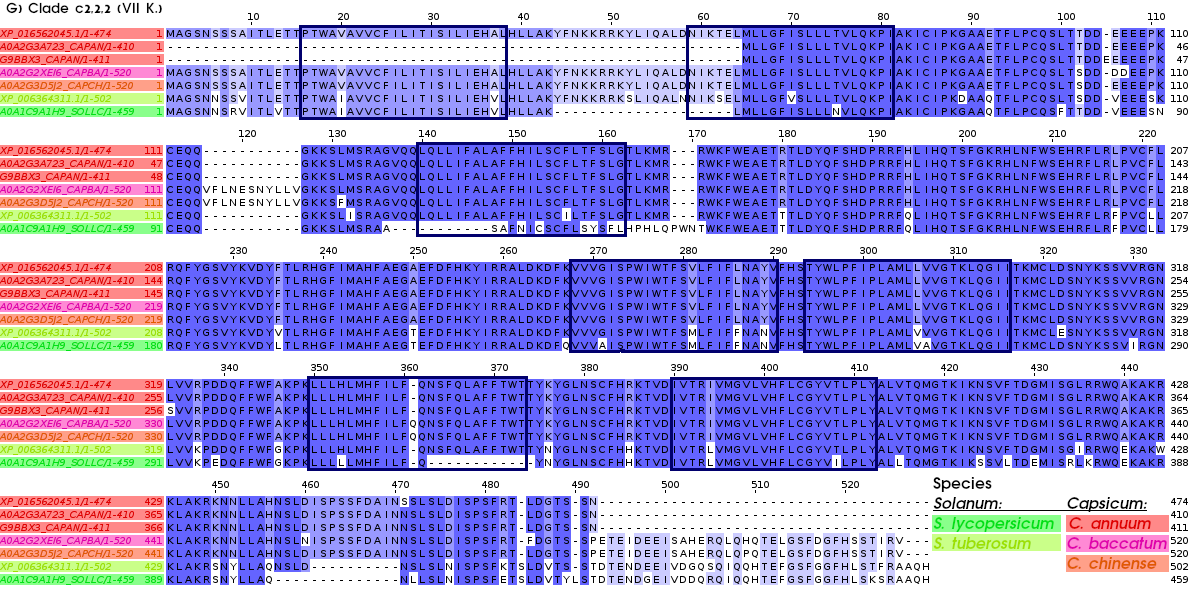

Supplement: Supplementary file 1 [file plants-11-01588-s001.zip › Supplementary Figure S3/Figure S3G.png]

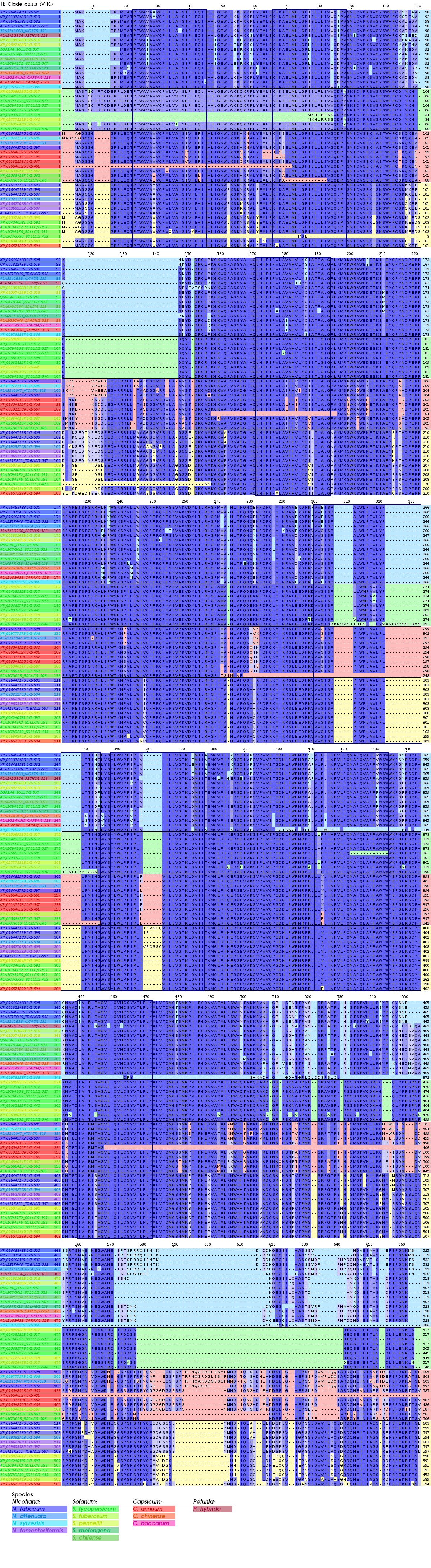

Supplement: Supplementary file 1 [file plants-11-01588-s001.zip › Supplementary Figure S3/Figure S3H.png]

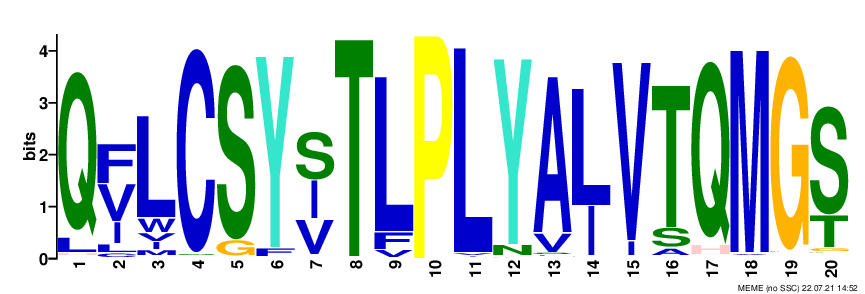

Supplement: Supplementary file 1 [file plants-11-01588-s001.zip › Supplementary File S2/meme200/logo1.png]

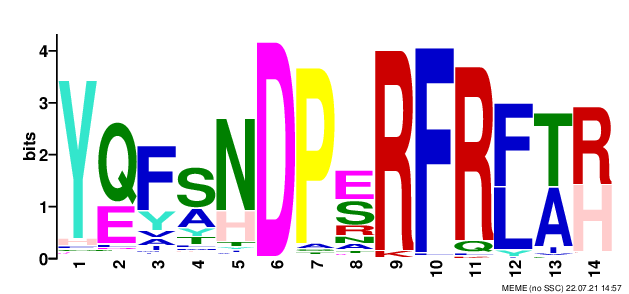

Supplement: Supplementary file 1 [file plants-11-01588-s001.zip › Supplementary File S2/meme200/logo10.png]

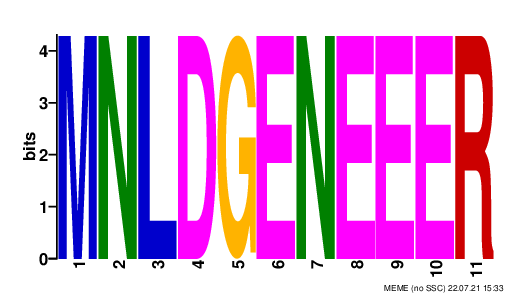

Supplement: Supplementary file 1 [file plants-11-01588-s001.zip › Supplementary File S2/meme200/logo100.png]

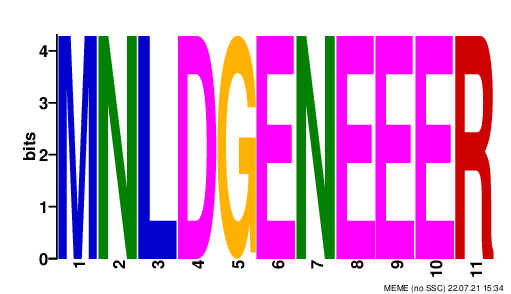

Supplement: Supplementary file 1 [file plants-11-01588-s001.zip › Supplementary File S2/meme200/logo101.png]

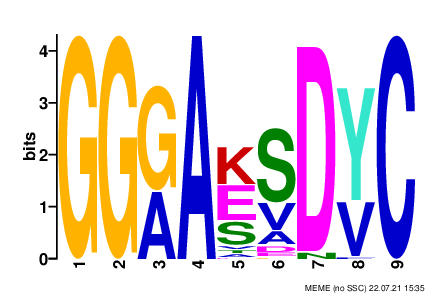

Supplement: Supplementary file 1 [file plants-11-01588-s001.zip › Supplementary File S2/meme200/logo103.png]

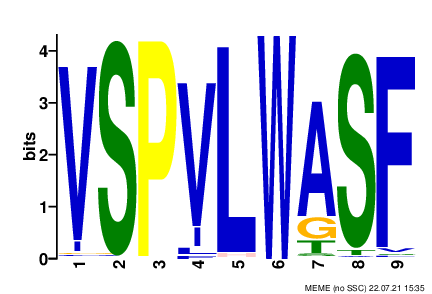

Supplement: Supplementary file 1 [file plants-11-01588-s001.zip › Supplementary File S2/meme200/logo104.png]

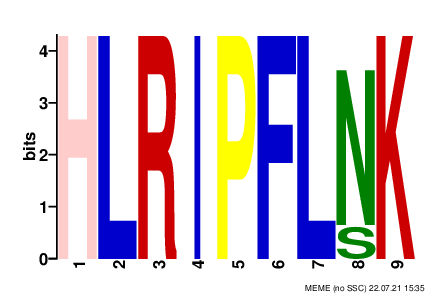

Supplement: Supplementary file 1 [file plants-11-01588-s001.zip › Supplementary File S2/meme200/logo105.png]

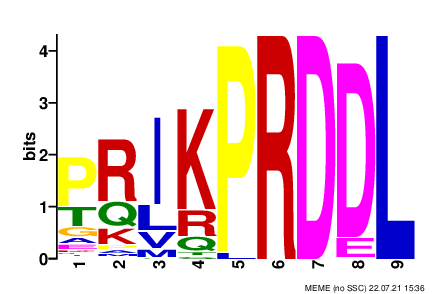

Supplement: Supplementary file 1 [file plants-11-01588-s001.zip › Supplementary File S2/meme200/logo106.png]

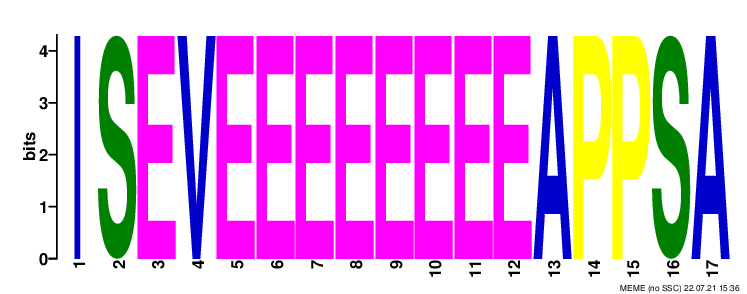

Supplement: Supplementary file 1 [file plants-11-01588-s001.zip › Supplementary File S2/meme200/logo107.png]

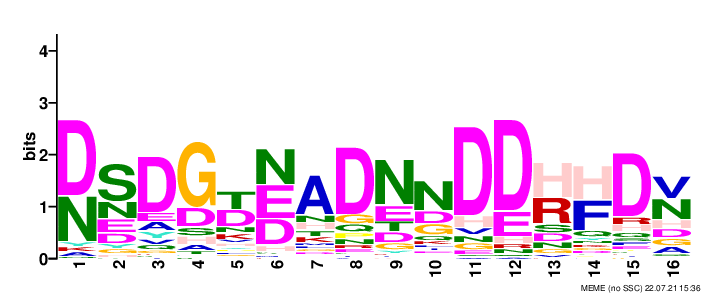

Supplement: Supplementary file 1 [file plants-11-01588-s001.zip › Supplementary File S2/meme200/logo108.png]

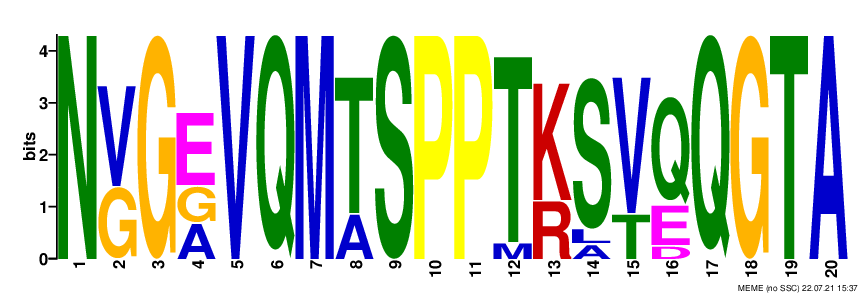

Supplement: Supplementary file 1 [file plants-11-01588-s001.zip › Supplementary File S2/meme200/logo109.png]

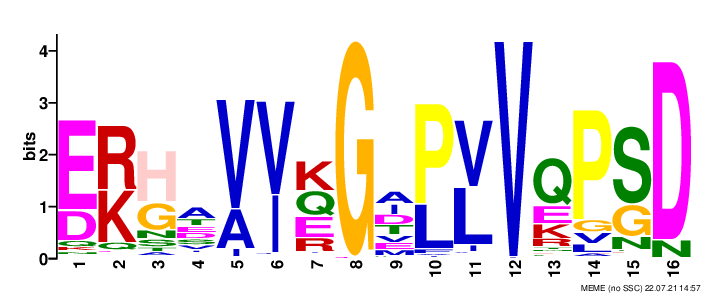

Supplement: Supplementary file 1 [file plants-11-01588-s001.zip › Supplementary File S2/meme200/logo11.png]

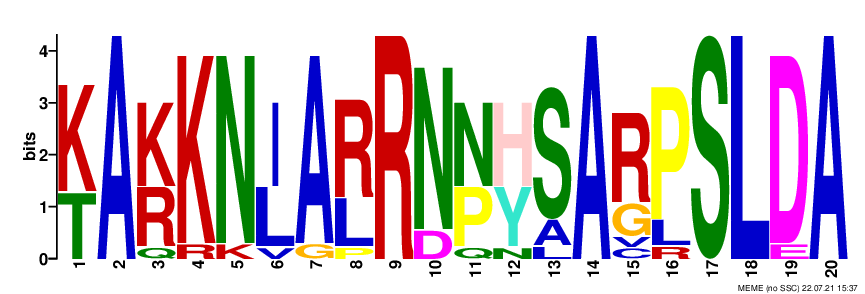

Supplement: Supplementary file 1 [file plants-11-01588-s001.zip › Supplementary File S2/meme200/logo110.png]

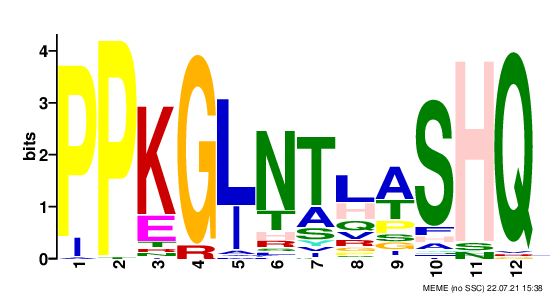

Supplement: Supplementary file 1 [file plants-11-01588-s001.zip › Supplementary File S2/meme200/logo111.png]

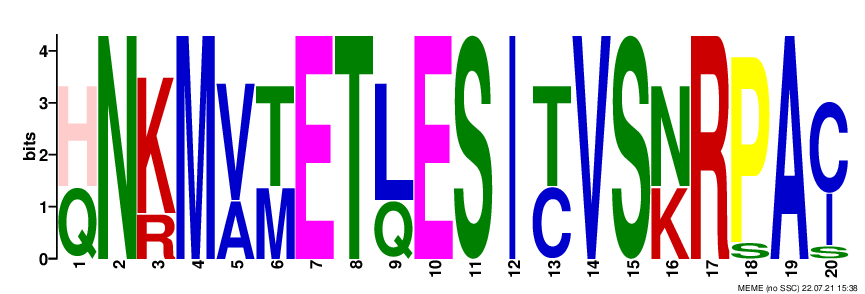

Supplement: Supplementary file 1 [file plants-11-01588-s001.zip › Supplementary File S2/meme200/logo112.png]

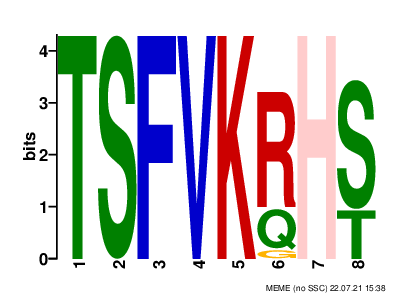

Supplement: Supplementary file 1 [file plants-11-01588-s001.zip › Supplementary File S2/meme200/logo113.png]

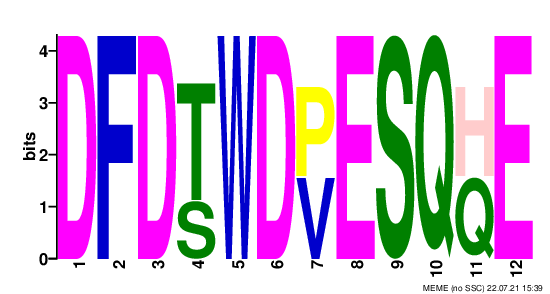

Supplement: Supplementary file 1 [file plants-11-01588-s001.zip › Supplementary File S2/meme200/logo114.png]

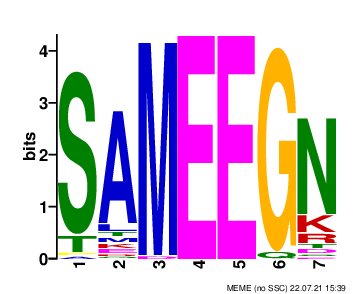

Supplement: Supplementary file 1 [file plants-11-01588-s001.zip › Supplementary File S2/meme200/logo115.png]

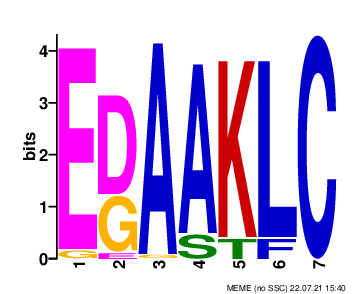

Supplement: Supplementary file 1 [file plants-11-01588-s001.zip › Supplementary File S2/meme200/logo116.png]

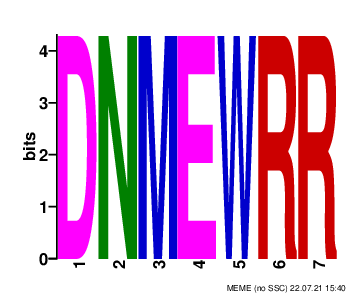

Supplement: Supplementary file 1 [file plants-11-01588-s001.zip › Supplementary File S2/meme200/logo117.png]

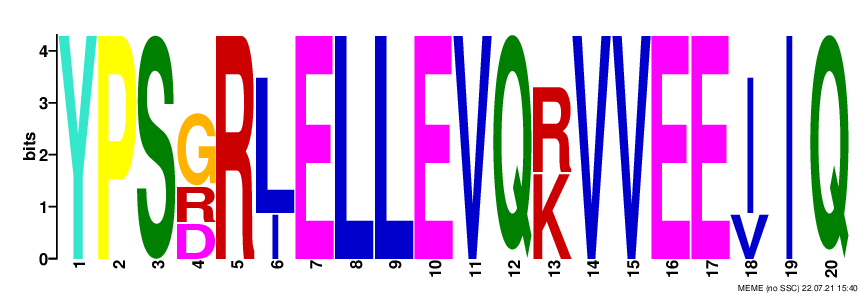

Supplement: Supplementary file 1 [file plants-11-01588-s001.zip › Supplementary File S2/meme200/logo118.png]

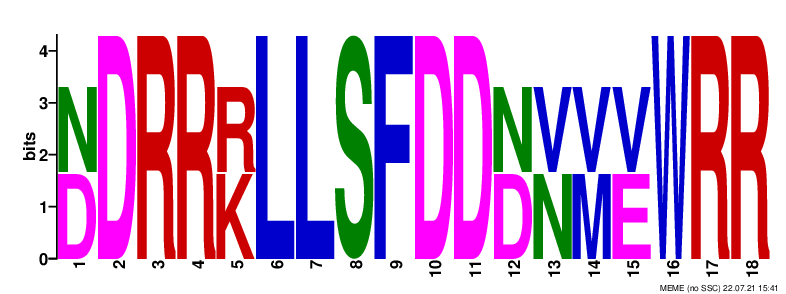

Supplement: Supplementary file 1 [file plants-11-01588-s001.zip › Supplementary File S2/meme200/logo119.png]

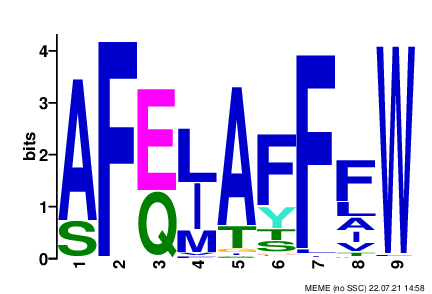

Supplement: Supplementary file 1 [file plants-11-01588-s001.zip › Supplementary File S2/meme200/logo12.png]

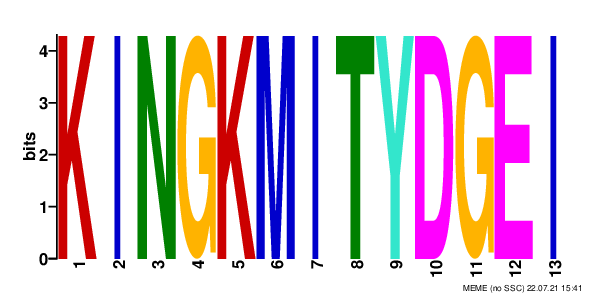

Supplement: Supplementary file 1 [file plants-11-01588-s001.zip › Supplementary File S2/meme200/logo120.png]

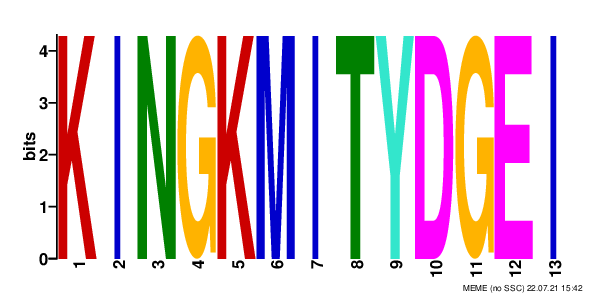

Supplement: Supplementary file 1 [file plants-11-01588-s001.zip › Supplementary File S2/meme200/logo121.png]

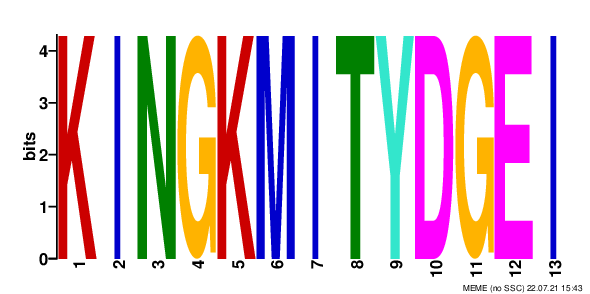

Supplement: Supplementary file 1 [file plants-11-01588-s001.zip › Supplementary File S2/meme200/logo122.png]

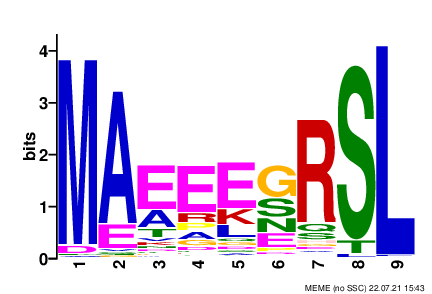

Supplement: Supplementary file 1 [file plants-11-01588-s001.zip › Supplementary File S2/meme200/logo123.png]

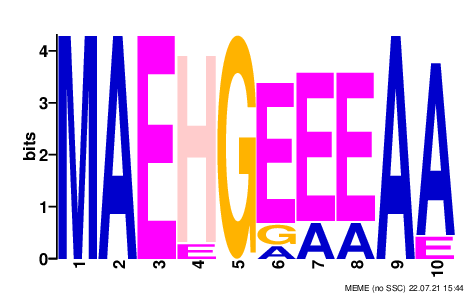

Supplement: Supplementary file 1 [file plants-11-01588-s001.zip › Supplementary File S2/meme200/logo124.png]

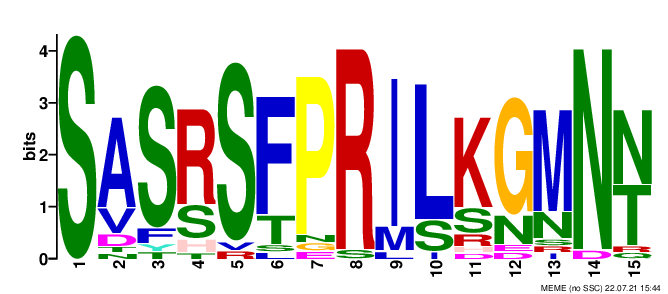

Supplement: Supplementary file 1 [file plants-11-01588-s001.zip › Supplementary File S2/meme200/logo125.png]

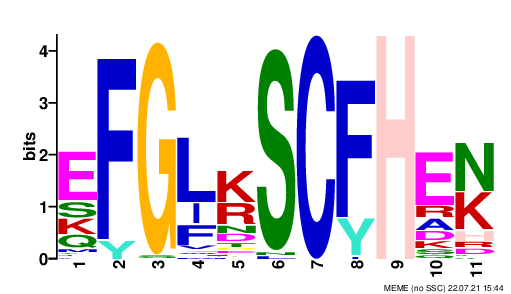

Supplement: Supplementary file 1 [file plants-11-01588-s001.zip › Supplementary File S2/meme200/logo126.png]

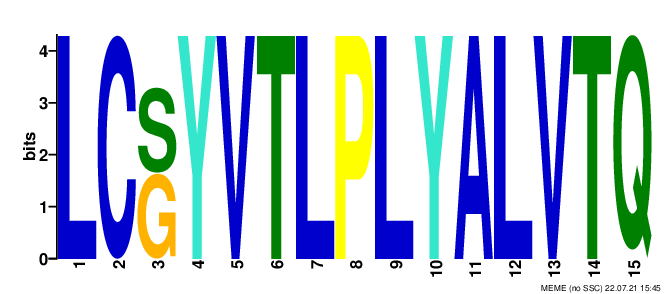

Supplement: Supplementary file 1 [file plants-11-01588-s001.zip › Supplementary File S2/meme200/logo127.png]

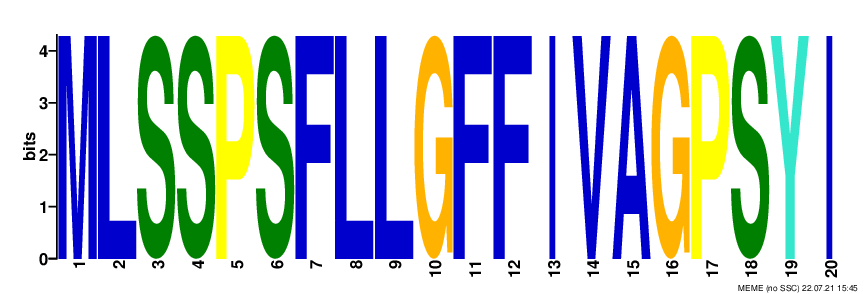

Supplement: Supplementary file 1 [file plants-11-01588-s001.zip › Supplementary File S2/meme200/logo128.png]

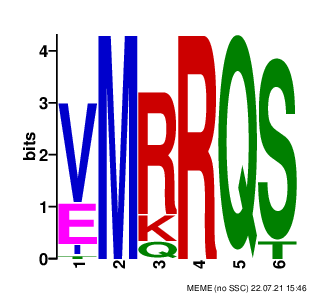

Supplement: Supplementary file 1 [file plants-11-01588-s001.zip › Supplementary File S2/meme200/logo129.png]

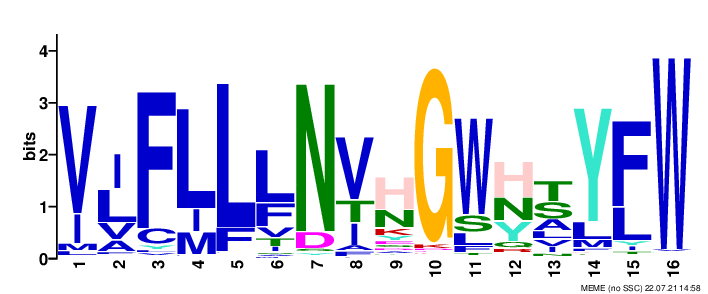

Supplement: Supplementary file 1 [file plants-11-01588-s001.zip › Supplementary File S2/meme200/logo13.png]

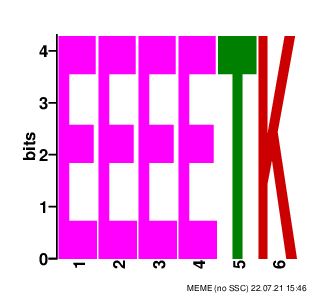

Supplement: Supplementary file 1 [file plants-11-01588-s001.zip › Supplementary File S2/meme200/logo130.png]

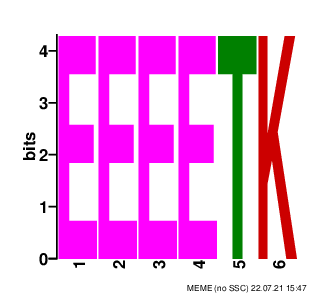

Supplement: Supplementary file 1 [file plants-11-01588-s001.zip › Supplementary File S2/meme200/logo132.png]

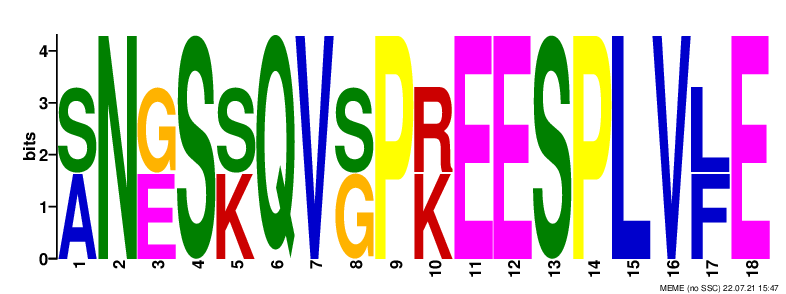

Supplement: Supplementary file 1 [file plants-11-01588-s001.zip › Supplementary File S2/meme200/logo133.png]

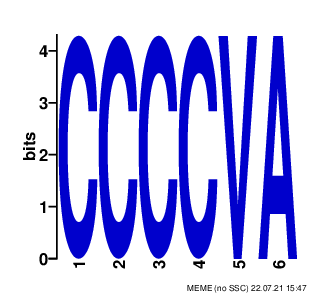

Supplement: Supplementary file 1 [file plants-11-01588-s001.zip › Supplementary File S2/meme200/logo134.png]

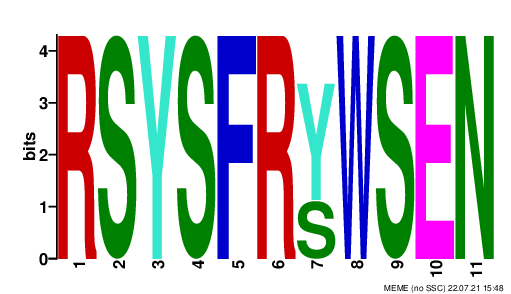

Supplement: Supplementary file 1 [file plants-11-01588-s001.zip › Supplementary File S2/meme200/logo135.png]

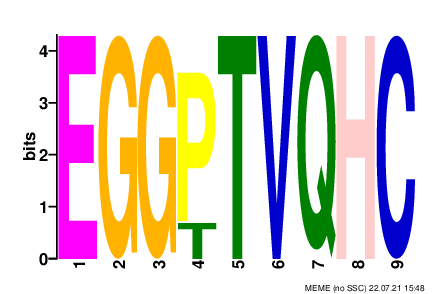

Supplement: Supplementary file 1 [file plants-11-01588-s001.zip › Supplementary File S2/meme200/logo136.png]

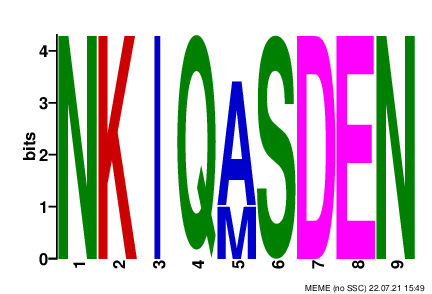

Supplement: Supplementary file 1 [file plants-11-01588-s001.zip › Supplementary File S2/meme200/logo137.png]

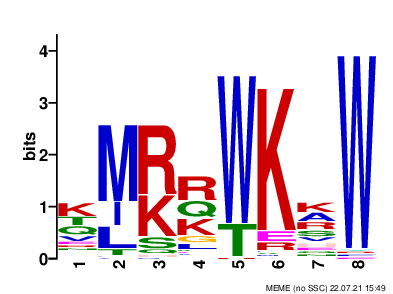

Supplement: Supplementary file 1 [file plants-11-01588-s001.zip › Supplementary File S2/meme200/logo138.png]

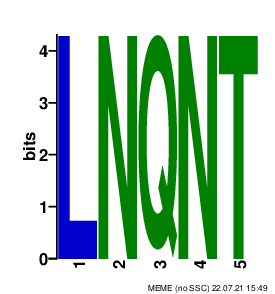

Supplement: Supplementary file 1 [file plants-11-01588-s001.zip › Supplementary File S2/meme200/logo139.png]

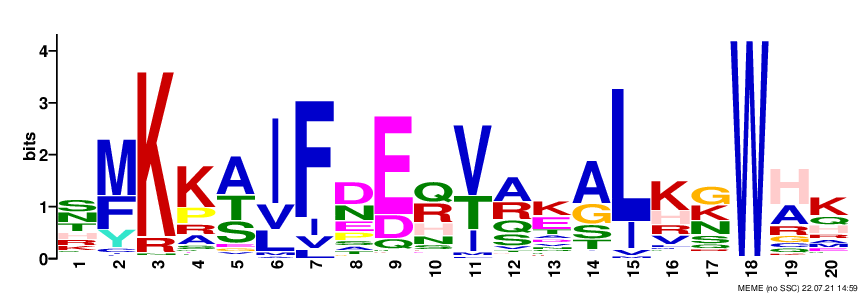

Supplement: Supplementary file 1 [file plants-11-01588-s001.zip › Supplementary File S2/meme200/logo14.png]

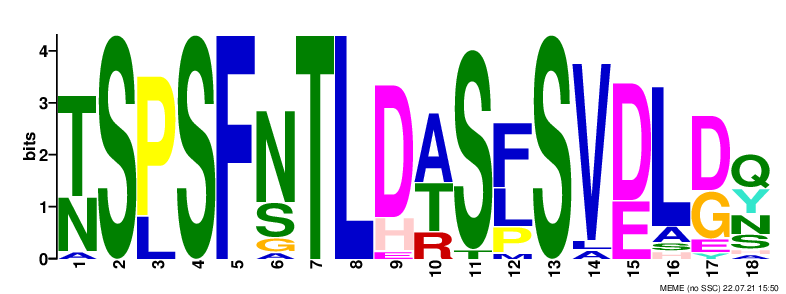

Supplement: Supplementary file 1 [file plants-11-01588-s001.zip › Supplementary File S2/meme200/logo140.png]

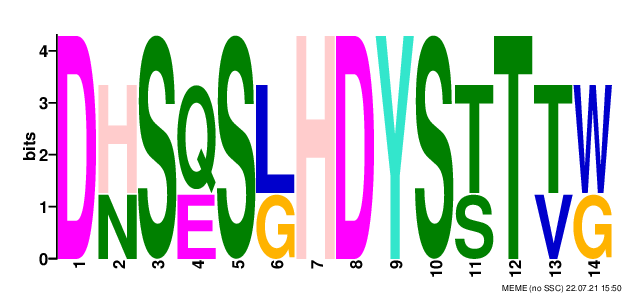

Supplement: Supplementary file 1 [file plants-11-01588-s001.zip › Supplementary File S2/meme200/logo141.png]

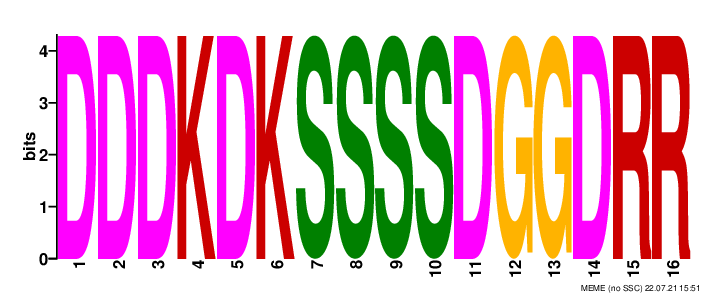

Supplement: Supplementary file 1 [file plants-11-01588-s001.zip › Supplementary File S2/meme200/logo142.png]

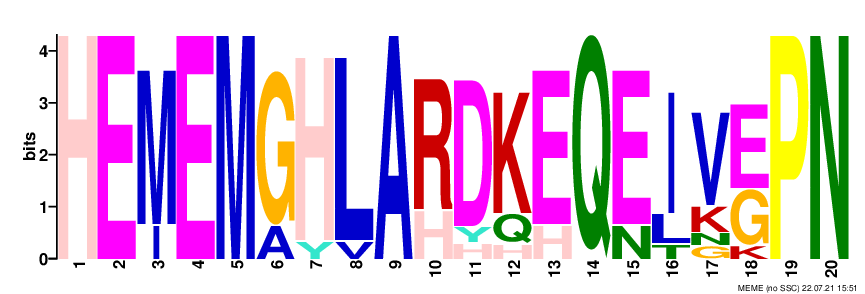

Supplement: Supplementary file 1 [file plants-11-01588-s001.zip › Supplementary File S2/meme200/logo143.png]

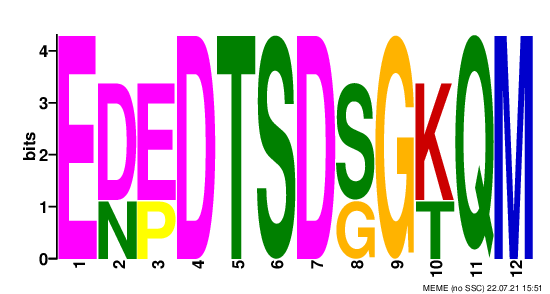

Supplement: Supplementary file 1 [file plants-11-01588-s001.zip › Supplementary File S2/meme200/logo144.png]

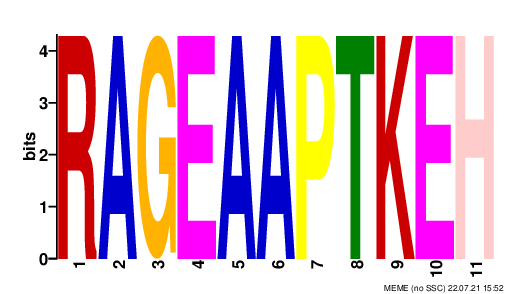

Supplement: Supplementary file 1 [file plants-11-01588-s001.zip › Supplementary File S2/meme200/logo145.png]

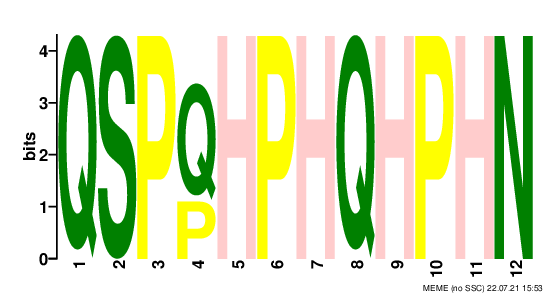

Supplement: Supplementary file 1 [file plants-11-01588-s001.zip › Supplementary File S2/meme200/logo148.png]

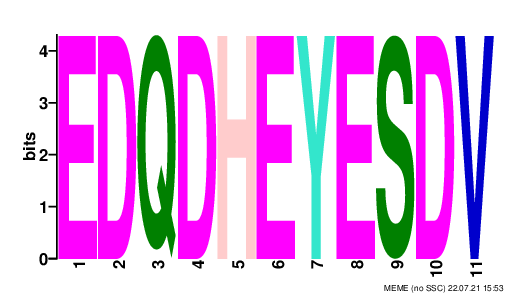

Supplement: Supplementary file 1 [file plants-11-01588-s001.zip › Supplementary File S2/meme200/logo149.png]

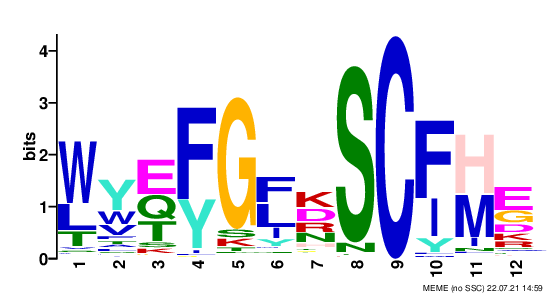

Supplement: Supplementary file 1 [file plants-11-01588-s001.zip › Supplementary File S2/meme200/logo15.png]

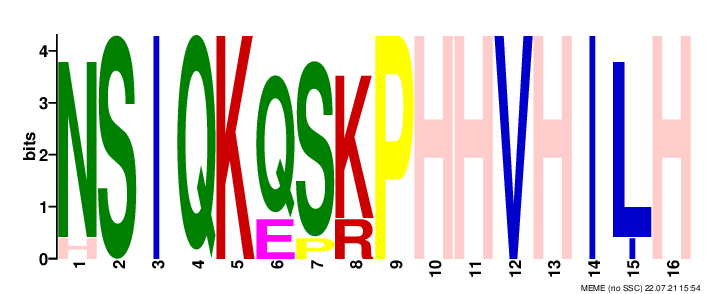

Supplement: Supplementary file 1 [file plants-11-01588-s001.zip › Supplementary File S2/meme200/logo150.png]

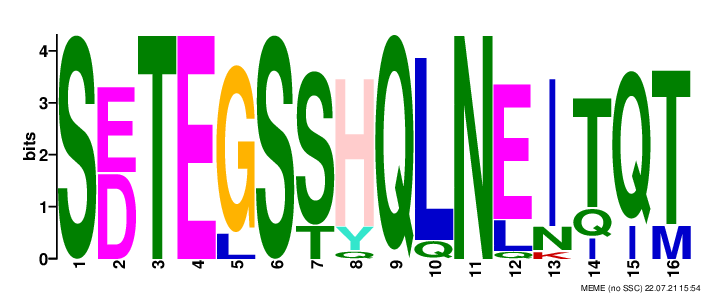

Supplement: Supplementary file 1 [file plants-11-01588-s001.zip › Supplementary File S2/meme200/logo151.png]

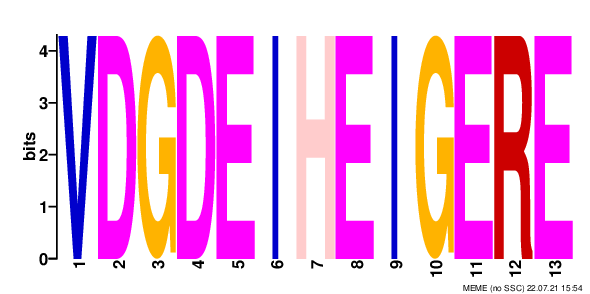

Supplement: Supplementary file 1 [file plants-11-01588-s001.zip › Supplementary File S2/meme200/logo152.png]

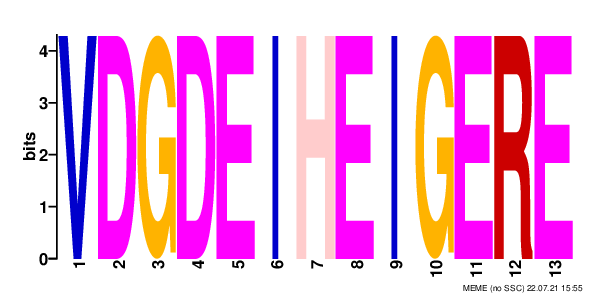

Supplement: Supplementary file 1 [file plants-11-01588-s001.zip › Supplementary File S2/meme200/logo153.png]

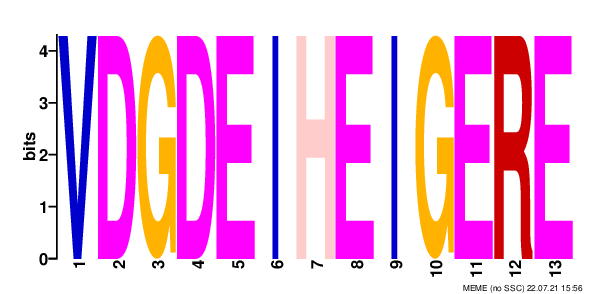

Supplement: Supplementary file 1 [file plants-11-01588-s001.zip › Supplementary File S2/meme200/logo155.png]

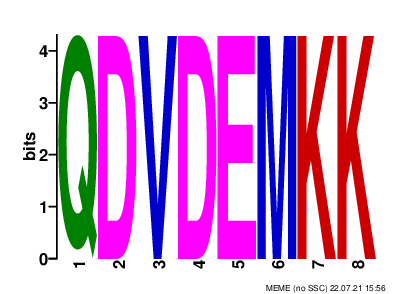

Supplement: Supplementary file 1 [file plants-11-01588-s001.zip › Supplementary File S2/meme200/logo156.png]

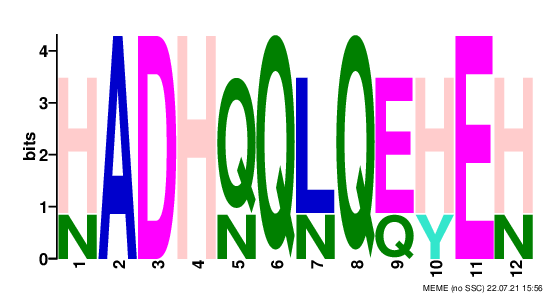

Supplement: Supplementary file 1 [file plants-11-01588-s001.zip › Supplementary File S2/meme200/logo157.png]

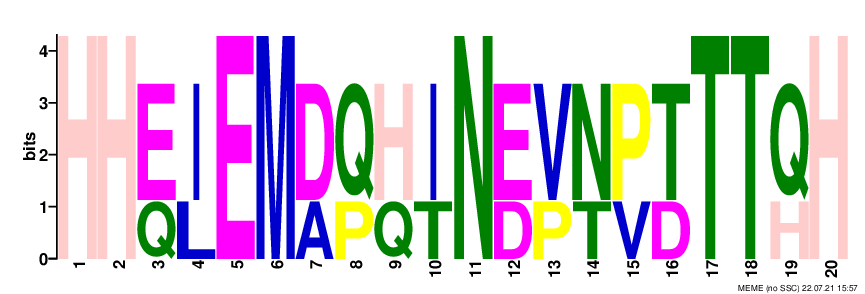

Supplement: Supplementary file 1 [file plants-11-01588-s001.zip › Supplementary File S2/meme200/logo158.png]

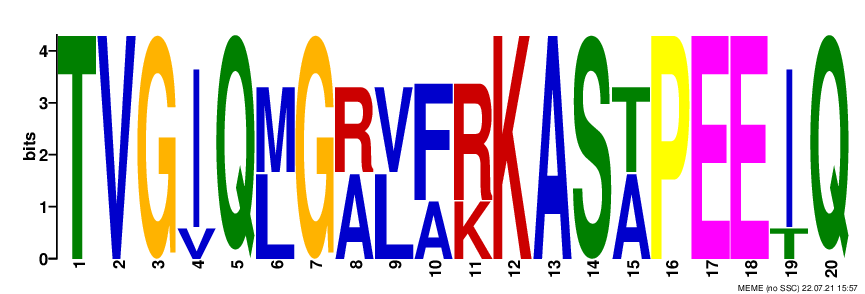

Supplement: Supplementary file 1 [file plants-11-01588-s001.zip › Supplementary File S2/meme200/logo159.png]

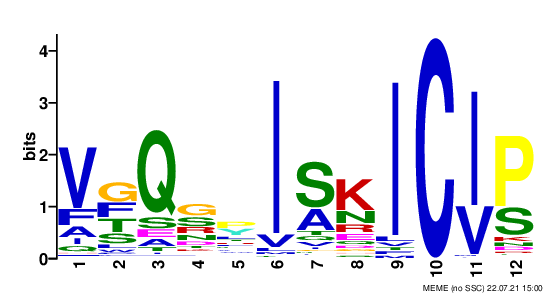

Supplement: Supplementary file 1 [file plants-11-01588-s001.zip › Supplementary File S2/meme200/logo16.png]

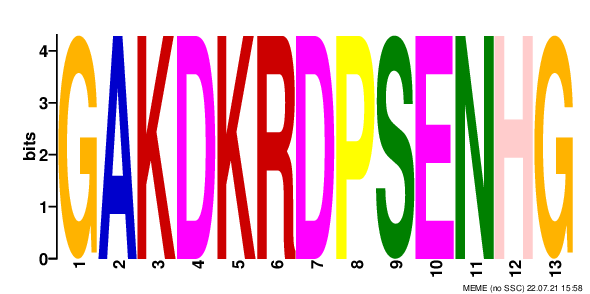

Supplement: Supplementary file 1 [file plants-11-01588-s001.zip › Supplementary File S2/meme200/logo160.png]

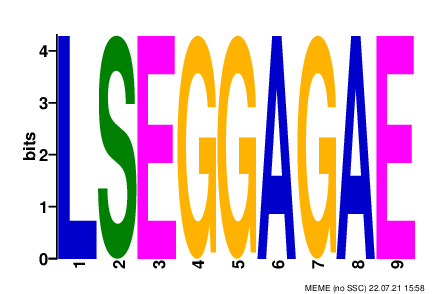

Supplement: Supplementary file 1 [file plants-11-01588-s001.zip › Supplementary File S2/meme200/logo161.png]

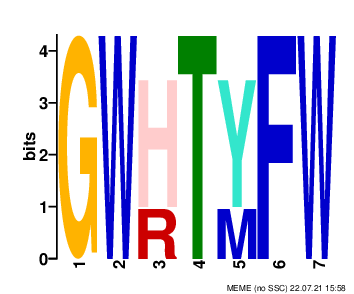

Supplement: Supplementary file 1 [file plants-11-01588-s001.zip › Supplementary File S2/meme200/logo162.png]

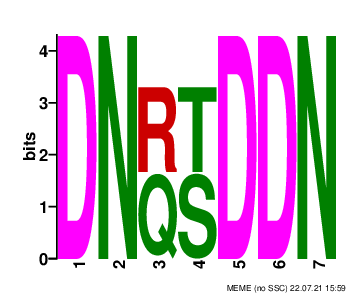

Supplement: Supplementary file 1 [file plants-11-01588-s001.zip › Supplementary File S2/meme200/logo163.png]

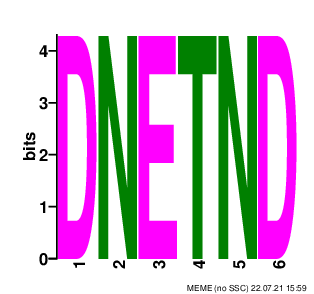

Supplement: Supplementary file 1 [file plants-11-01588-s001.zip › Supplementary File S2/meme200/logo164.png]

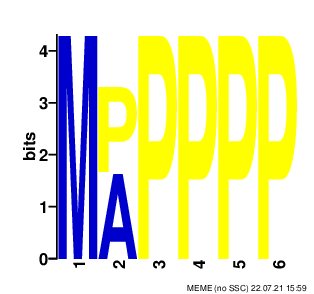

Supplement: Supplementary file 1 [file plants-11-01588-s001.zip › Supplementary File S2/meme200/logo165.png]

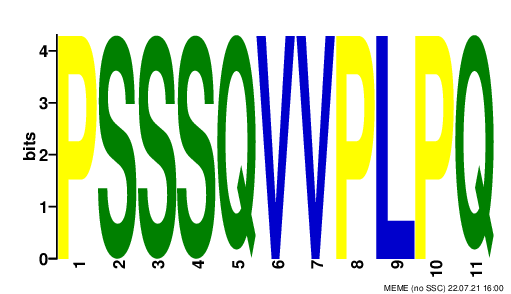

Supplement: Supplementary file 1 [file plants-11-01588-s001.zip › Supplementary File S2/meme200/logo166.png]

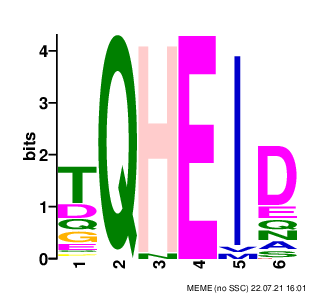

Supplement: Supplementary file 1 [file plants-11-01588-s001.zip › Supplementary File S2/meme200/logo168.png]

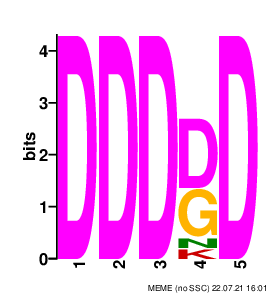

Supplement: Supplementary file 1 [file plants-11-01588-s001.zip › Supplementary File S2/meme200/logo169.png]

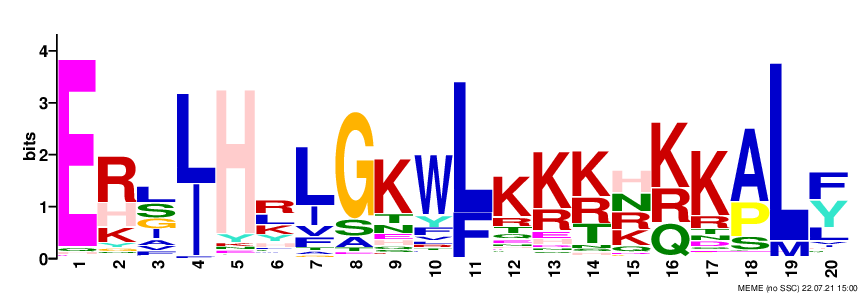

Supplement: Supplementary file 1 [file plants-11-01588-s001.zip › Supplementary File S2/meme200/logo17.png]

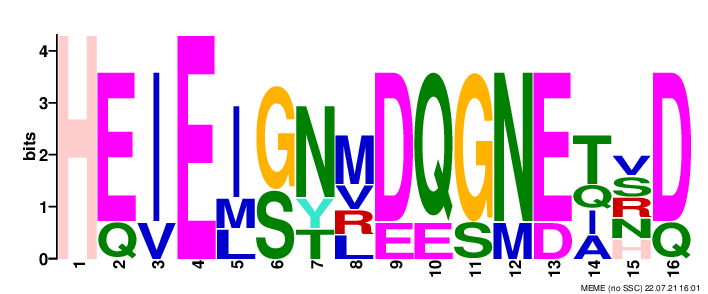

Supplement: Supplementary file 1 [file plants-11-01588-s001.zip › Supplementary File S2/meme200/logo170.png]

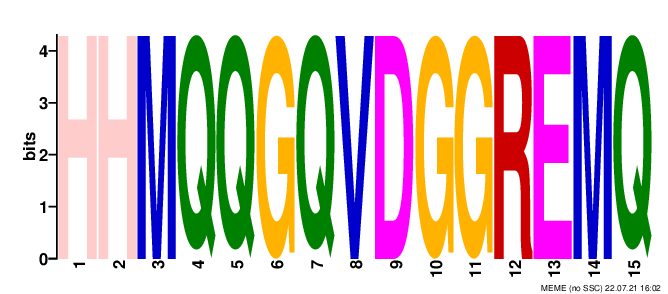

Supplement: Supplementary file 1 [file plants-11-01588-s001.zip › Supplementary File S2/meme200/logo171.png]

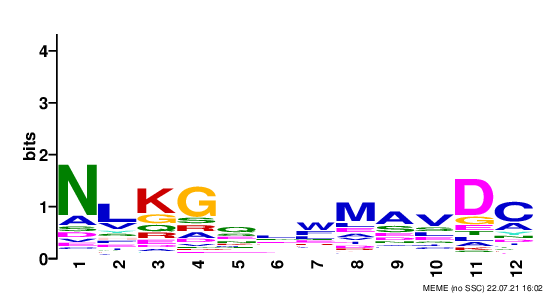

Supplement: Supplementary file 1 [file plants-11-01588-s001.zip › Supplementary File S2/meme200/logo172.png]

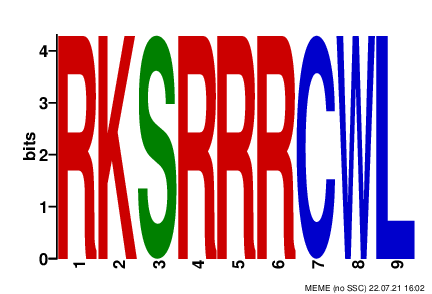

Supplement: Supplementary file 1 [file plants-11-01588-s001.zip › Supplementary File S2/meme200/logo173.png]

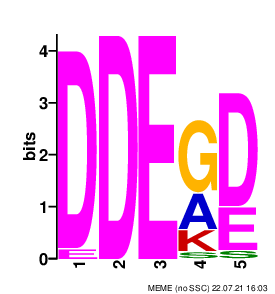

Supplement: Supplementary file 1 [file plants-11-01588-s001.zip › Supplementary File S2/meme200/logo174.png]

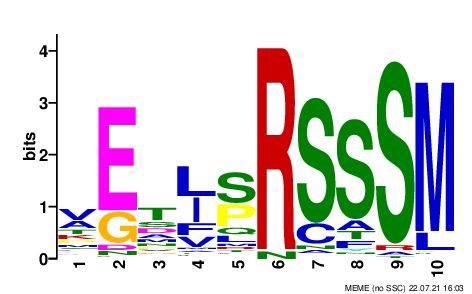

Supplement: Supplementary file 1 [file plants-11-01588-s001.zip › Supplementary File S2/meme200/logo175.png]

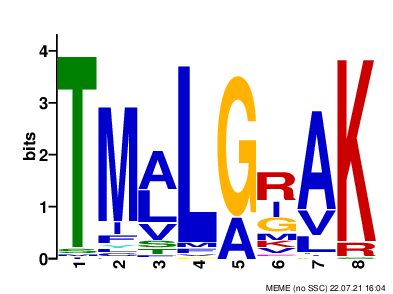

Supplement: Supplementary file 1 [file plants-11-01588-s001.zip › Supplementary File S2/meme200/logo176.png]

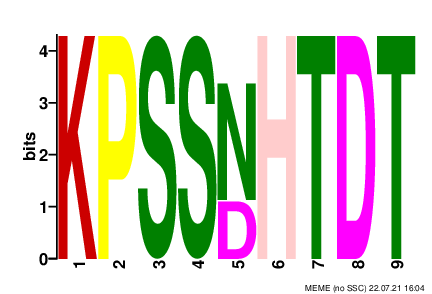

Supplement: Supplementary file 1 [file plants-11-01588-s001.zip › Supplementary File S2/meme200/logo177.png]

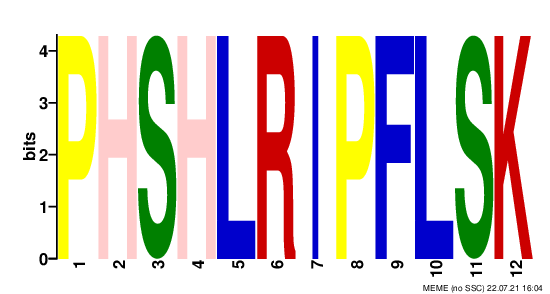

Supplement: Supplementary file 1 [file plants-11-01588-s001.zip › Supplementary File S2/meme200/logo178.png]

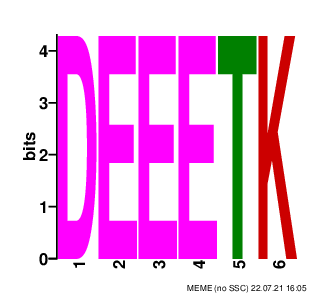

Supplement: Supplementary file 1 [file plants-11-01588-s001.zip › Supplementary File S2/meme200/logo179.png]

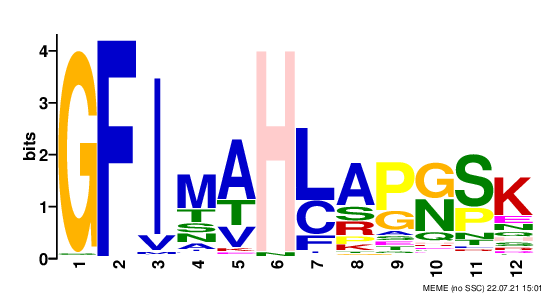

Supplement: Supplementary file 1 [file plants-11-01588-s001.zip › Supplementary File S2/meme200/logo18.png]

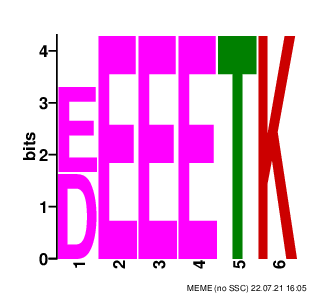

Supplement: Supplementary file 1 [file plants-11-01588-s001.zip › Supplementary File S2/meme200/logo180.png]
